# Identification of Immune-Related lncRNAs for Predicting Prognosis and Immune Landscape Characteristics of Uveal Melanoma

**DOI:** 10.1155/2022/7680657

**Published:** 2022-08-29

**Authors:** Wei Chen, Liying Yan, Bo Long, Li Lin

**Affiliations:** Department of Ophthalmology, Suining Central Hospital, No. 127, West Desheng Road, Chuanshan District, Suining 629000, Sichuan Province, China

## Abstract

Immune-related genes and long noncoding RNAs (lncRNAs) have a significant impact on the prognostic value and immunotherapeutic response of uveal melanoma (UM). Therefore, we tried to develop a prognostic model on the basis of irlncRNAs for predicting prognosis and response on immunotherapy of UM patients. We identified 1,664 immune-related genes and 2,216 immune-related lncRNAs (irlncRNAs) and structured a prognostic model with 3 prognostic irlncRNAs by co-expression analysis, univariable Cox, LASSO, and multivariate Cox regression analyses. The Kaplan–Meier analysis indicated that patients in the high-risk group had a shorter survival time than patients in the low-risk group. The ROC curves demonstrated the high sensitivity and specificity of the signature for survival prediction, and the one-, three-, and five-year AUC values, respectively, were 0.974, 0.929, and 0.941 in the entire set. Cox regression analysis, C-index, DCA, PCA analysis, and nomogram were also applied to assess the validity and accuracy of the risk model. The GO and KEGG enrichment analyses indicated that this signature is significantly related to immune-related pathways and molecules. Finally, we investigated the immunological characteristics and immunotherapy of the model and identified various novel potential compounds in the model for UM. In summary, we constructed a new model on the basis of irlncRNAs that can accurately predict prognosis and response on immunotherapy of UM patients, which may provide valuable clinical applications in antitumor immunotherapy.

## 1. Introduction

Uveal melanoma (UM) is the commonest primary intraocular malignancy in adults, contributing up to 85% of ocular melanomas, and more than half of individuals with UM experience systemic metastatic disease [1, 2]. UM mainly originates from the choroid, iris, and ciliary body and has a prevalence of 5.1 per million in America [3, 4]. Over the past 30 years, although local treatments for UM have been developed, 5-year survival rates have not changed, and no effective complementary therapy is currently available to decrease the risk of metastasis from UM [5, 6]. In addition, because of the high heterogeneity of UM, patients with identical stages receive similar treatment but showed very diverse prognostic outcomes [7]. Hence, the identification of dependable prognostic biomarkers is essential for personalized treatment.

Noticeably, immunotherapy remarkably improved the prognosis of patients with cutaneous melanoma, yet poorly improved UM [4]. Previous retrospective studies have shown a low immune response rate to immunotherapy in UM patients, such as 10-21% for the combination of ibritumomab and nilumab, 3.6% for anti-PD-1 antibodies, and 5% for ibritumomab monotherapy [8–12]. Long noncoding RNAs (LncRNAs) are engaged in tumor cell proliferation, invasive metastasis, apoptosis, drug resistance, immune escape, and so on due to their influence on oncogenes and oncogenes of tumors [13, 14]. Therefore, lncRNAs are considered a highly promising candidate for personalized medicine for UM patients as a biomarker as well as a potential therapeutic target.

In this research, we identified the immune-related lncRNAs (irlncRNAs) and developed a risk assessment model by using univariable Cox, LASSO, and multivariate Cox regression analyses. Also, we evaluated the immunological atlas and found a variety of new potential therapeutic drugs in the model. In summary, we developed a risk assessment model for UM on the basis of irlncRNAs that can predict the prognosis and immunotherapy response in UM patients.

## 2. Methods and Materials

### 2.1. Preparation of Data

RNA-seq data and clinical information for UM were gathered using The Cancer Genome Atlas (TCGA, https://tcga-data.nci.nih.gov/tcga). Annotations based on the Ensembl database (http://asia.ensembl.org) were used to derive mRNA and lncRNA expression patterns. The ImmPort database (http://www.immport.org) was utilized to derive the expression patterns of immune-related genes. In order to identify irlncRNAs, the cor >0.4 and *p*^*∗*^0.001 criteria were applied using the R package limma.

### 2.2. Establishment of Risk Assessment Model

A training subset and a test subset were created from the whole TCGA dataset. The whole set was utilized to identify prognosis of irlncRNAs, LASSO regression analysis was used to filter these prognosis of irlncRNAs, and multivariate Cox regression analysis was used to examine the remaining prognosis of irlncRNAs, resulting in a prognostic risk model. Each patient had a unique risk score, which was determined using the following formula:∑_*i*=1_^*k*^*βisi*. UM patients were assigned to high- and low-risk groups on the basis of their median risk score.

### 2.3. Validation of Prognostic Model

The Kaplan–Meier analysis was performed to compare the survival rates of the high-risk and low-risk groups. The area under the curve (AUC) and time-dependentreceiver-operating characteristic (ROC) curves were applied to assess the model's ability to predict survival when compared to standard clinicopathological features. Cox analyses, both univariate and multivariate, were utilized to confirm that the model was an independent determinant of prognosis. To examine the model's accuracy in comparison to standard clinicopathological features, the concordance index (C-index) and decision curve analysis (DCA) were used. A nomogram integrating prognostic signatures was constructed to predict the one-, three-, and five-year survival rates of patients. The whole gene expression profiles, immune-genes, irlncRNAs, and the irlncRNAs in the model were subjected to a principal component analysis (PCA) study for exploratory display of high-dimensional data.

### 2.4. Exploration of Immunological Atlas

The Gene Ontology (GO) *p* < 0.05 and Kyoto Encyclopedia of Genes and Genomes (KEGG) *p* < 0.05 enrichment analyses for differentially expressed genes were used to investigate possible causes of prognostic variations in various risk categories. In order to obtain a valid assessment of immune infiltration, we employed the current established methodologies, including xCELL, TIMER, quanTIseq, MCP-counter, EPIC, CIBERSORT-ABS, and CIBERSORT. In our study, Wilcoxon signed rank test was used to compare the expression levels of immune checkpoint inhibitors (ICIs)-related molecules between groups. Single-sample gene set enrichment analysis (ssGSEA) was used to determine whether immune function differed between groups. Using the tumor immune dysfunction and exclusion (TIDE) method, we were able to predict the variation in immunotherapeutic responses between groups.

### 2.5. Identification of Potential Drugs

Based on the Genomics of Drug Sensitivity in Cancer (GDSC, https://www.cancerrxgene.org), we calculated the half inhibitory concentration (IC50) of compounds. In addition, we used Wilcoxon signed rank testing to identify potential compounds for UM treatment in the clinic based on the difference in IC50 between different groups.

## 3. Results

### 3.1. Identification of Immune-Related lncRNAs

TCGA was employed to acquire RNA-seq data and clinical information for UM, which included 80 tumor samples. On the basis of the given data, we extracted expression profiles for 1,664 immune-related genes and 16,876 long noncoding RNAs. As a result of the co-expression analysis, 2,216 irlncRNAs were identified (cor >0.4 and *p* 0.001).

### 3.2. Construction of Prognostic Risk Model

**Figure 1:**
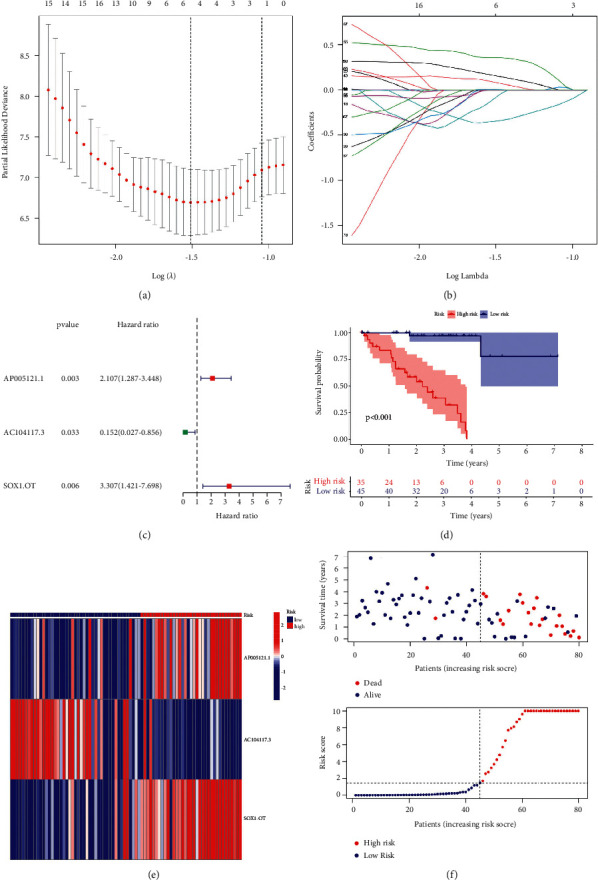
(a) LASSO coefficient profiles. (b) Coefficient profile plot generated against the log sequence. (c) 3 prognostic irlncRNAs identified by multivariate Cox regression analysis. (d) Kaplan–Meier survival curve of the model in the entire set. (e) The expression of the 3 prognostic irlncRNAs, patterns of survival outcome, and distribution of risk score for patients between different groups in the whole set.

Based on a ratio of 1 : 1, the entire TCGA set (80 samples) was randomly allocated to a training set (40 samples) and a testing set (40 samples), and a risk model was built by the entire set. We screened 409 prognostic irlncRNAs using univariate Cox regression analysis from a total of 2,216 irlncRNAs (*p* 0.05; Supplementary Table1). The LASSO regression analysis was utilized to filter out 6 candidate irlncRNAs from a total of 409 prognostic irlncRNAs, as shown in Figures 1(a) and 1(b), with the associated LASSO coefficient profiles and a partial likelihood deviation plot. Finally, a risk assessment model on the basis of multivariate Cox regression analysis was developed, which incorporated 3 irlncRNAs (AP005121.1, AC104117.3, and SOX1-OT; Figure 1(c)). Supplementary Table 2 demonstrates the baseline features of these datasets, with no statistically significant variations in clinical features (*p* > 0.05).

### 3.3. Validation of Risk Assessment Model

**Figure 2:**
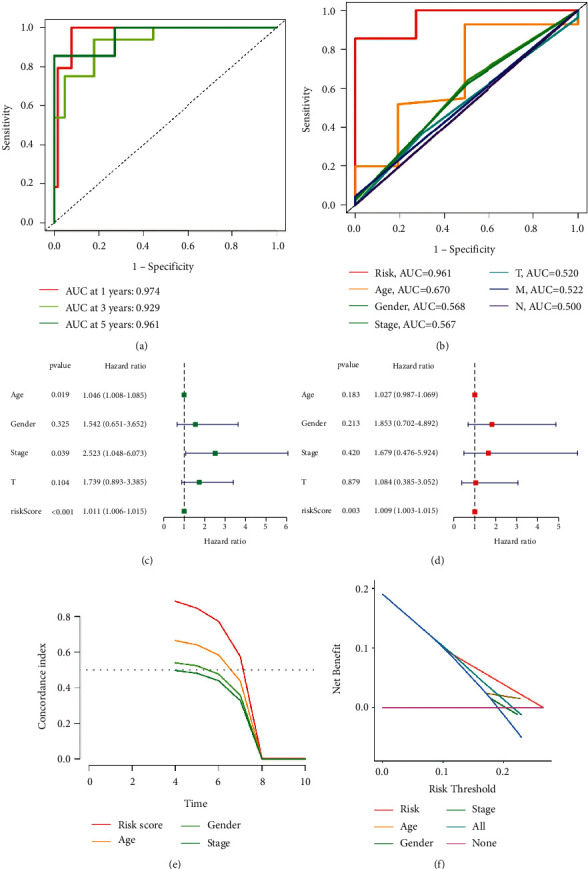
(a) One-, three-, and five-year AUC values, respectively, were 0.974, 0.929, and 0.941 in the entire set. (b) The five year AUC values of the model were higher than the traditional clinicopathological characteristics. (c) Univariate Cox regression analysis demonstrated that risk score was statistically associated with prognosis. (d) Multivariate Cox regression analysis showed that the risk score was an individual prognostic risk factor. (e, f) The C-index and DCA demonstrated that the signature better forecasted the prognosis of UM than other traditional clinicopathological characteristics.

The survival analysis, irlncRNA expression profiles, pattern of survival status, and distribution of risk grades between different groups were described in the entire set (Figures 1(d) and 1(e)), the training set (Supplementary Figure 1(a)), and the testing set (Supplementary Figure 1(b)), indicating that patients in the high-risk group had a shorter survival time than patients in the low-risk group. The ROC curves demonstrated the high sensitivity and specificity of the signature for survival prediction, and the one-, three-, and five-year AUC values, respectively, were 0.974, 0.929, and 0.941 in the entire set (Figure 2(a)), 0.967, 0.886, and 0.964 in the testing set (Supplementary Figure 2(a)), and 0.974, 0.924, and 0.939 in the training set (Supplementary Figure 2(b)). And, the five-year AUC values of the model were higher than the traditional clinicopathological characteristics (Figure 2(b)).

**Figure 3:**
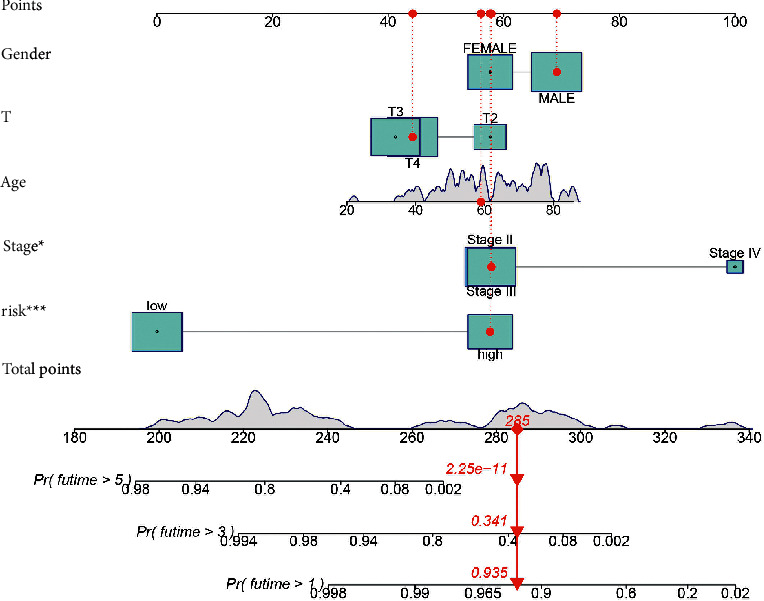
A nomogram including the model and clinicopathological characteristics was reliable and sensitive and can be applied to predict UM patient survival.

**Figure 4:**
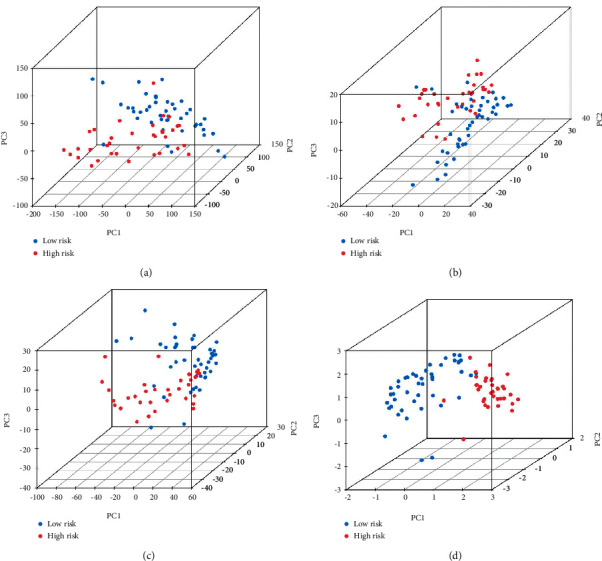
(a–d) PCA indicated that the distributions of entire gene expression profiles, immune-related genes, and irlncRNAs between different groups were relatively scattered, while the distributions of 3 irlncRNAs in the signature between different groups had different distributions.

Risk score was shown to be a significant prognostic risk factor in univariate Cox regression analysis (*p* = 0.001; Figure 2(c)) and an independent prognostic risk factor in multivariate Cox regression analysis (*p* = 0.003; Figure 2(d)). The risk model predicted the prognosis of UM better than other standard clinicopathological features, according to the C-index and DCA (Figures 2(e) and 2(f)). The signature and clinicopathological features nomogram was found to be trustworthy and sensitive, and it may be used to predict UM patient survival (Figure 3). The nomogram's calibration plot predicts the probability of a one-, three-, and five-year prognosis (Supplementary Figure 2(c)). PCA indicated that the distributions of entire gene expression profiles, immune-related genes, and irlncRNAs between different groups were relatively scattered (Figures 4(a)–4(c)), while the distributions of 3 irlncRNAs in the signature between different groups had different distributions (Figure 4(d)).

### 3.4. Exploration of Functional Enrichment

**Figure 5:**
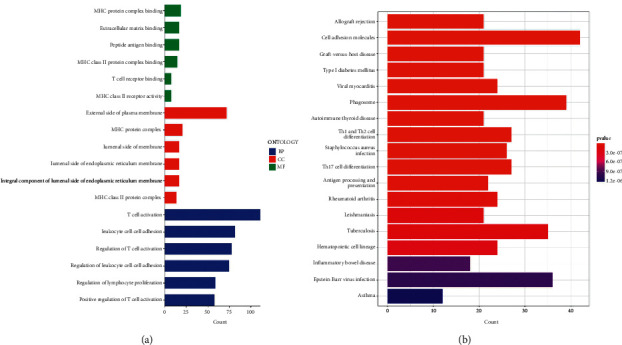
(a, b) GO and KEGG enrichment analyses (*p* < 0.05).

We used GO and KEGG enrichment analyses to study the underlying molecular processes of the irlncRNAs model. Immune cell activation, proliferation, and adhesion, as well as MHC binding, were all shown to be involved in the GO enrichment analysis, *p* < 0.05 (Figure 5(a) and Supplementary Table 3). Immunological system illnesses, immune responses, and immune cell differentiation were all shown to be involved in the KEGG enrichment analysis, *p* < 0.05 (Figure 5(b) and Supplementary Table 4).

### 3.5. Exploration of Immunological Atlas

**Figure 6:**
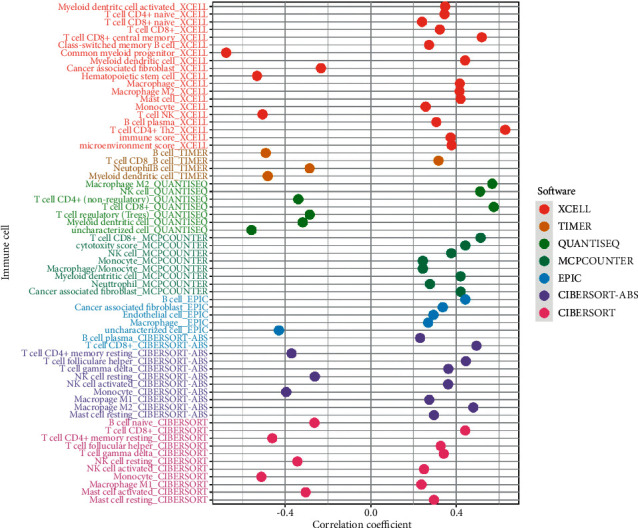
In the high-risk group, CD4+ T cells, CD8+ T cells, NK cells, M1 macrophages, M2 macrophages, myeloid dendritic cells, and fibroblasts were more abundant, whereas mast cells were more abundant in the low-risk group.

**Figure 7:**
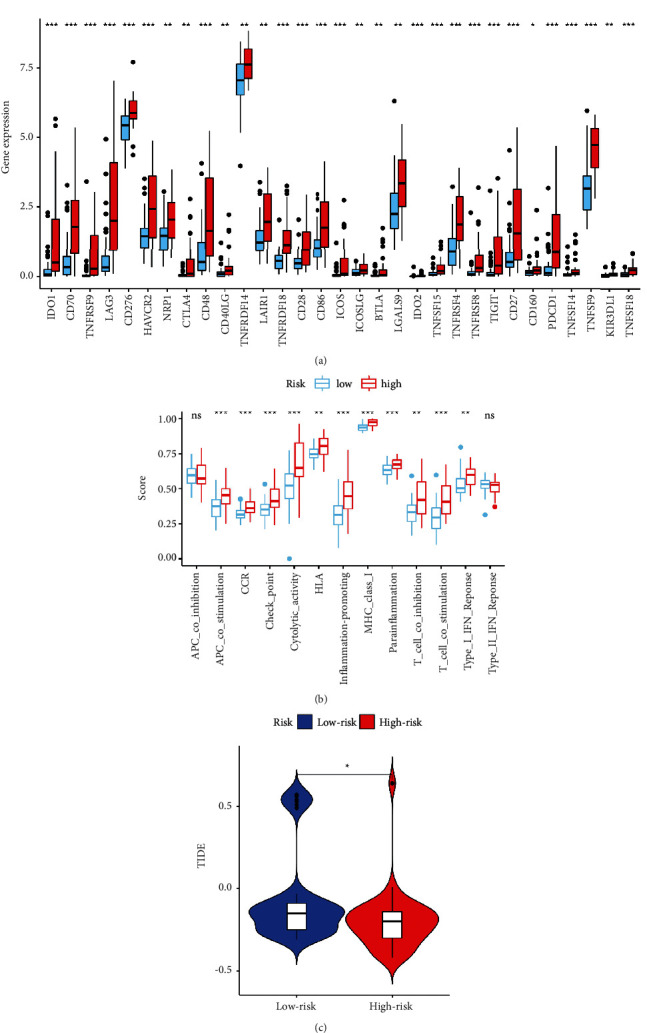
(a) Expression of the majority of ICIs-related molecules was higher in the high-risk group. (b) The majority of immune functions were statistically different between high- and low-risk groups, except for APC co-inhibition and type II IFN response. (c) TIDE scores were higher in the high-risk group.

In terms of immune cell infiltration, the high-risk group had more CD4+ T cells, CD8+ T cells, NK cells, M1 macrophages, M2 macrophages, myeloid dendritic cells, and fibroblasts, whereas the low-risk group had more mast cells (Figure 6). CTLA-4 (*p* 0.01), PDCD1 (*p* 0.001), LAG3 (*p* 0.001), TIGIT (*p* 0.001), and BTLA (*p* 0.01), among other genes, were found to be substantially different between different groups (Figure 7(a)). Apart from APC co-inhibition and type II IFN response, the bulk of immune activities were statistically distinct between different groups (Figure 7(b)). TIDE scores were higher in the high-risk group than in the low-risk group (*p* 0.05), indicating that the high-risk group was more likely to respond to immunotherapy (Figure 7(c)).

### 3.6. Recognized Potential Compounds

**Figure 8:**
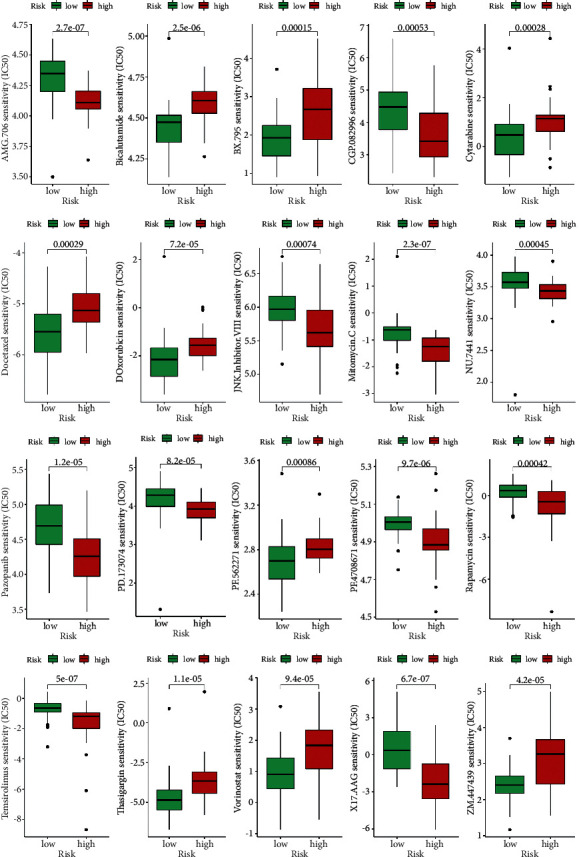
Identification of potential drugs targeting the model (*p* < 0.001).

Along with immunotherapy, we searched for potential compounds that target our signature for treating UM patients. Finally, we discovered that various agents (AMG.706, bicalutamide, BX.795, etc.) were identified for significant differences in the estimated IC50 between high- and low-risk groups (Figure 8 and Supplementary Figure S3).

## 4. Discussion

Currently, research for the UM model on the basis of lncRNAs is still scarce. Chen et al. recognized six autophagy-associated lncRNAs and constructed a signature, which can predict the prognosis of UM patients [15]. Liao et al. established an eight prognostic microenvironment-related lncRNAs signature and identified potential small molecule drugs [16]. In summary, we established for the first time a risk assessment model on the basis of irlncRNAs that can predict the prognosis and immunotherapy response in UM patients.

While immunotherapy has significantly improved the therapeutic regimens to cutaneous melanoma, its efficacy in UM has not been as dramatic. The eye is associated with many positive immunosuppressive mechanisms compared to other parts of the tissue [17–19]. Based on previous studies, lncRNAs and immune-related genes have been frequently used for model construction and subtype identification, and promising results have been observed [20–22]. We were motivated by the function of immune-related genes and lncRNAs in UM and tried to construct a prognostic risk model on the basis of irlncRNAs.

In the research, 2,216 irlncRNAs were identified to investigate the prognostic function of irlncRNAs. Then, 409 irlncRNAs were associated with prognosis, 6 candidate irlncRNAs (ELFN1-AS1, AF131216.4, AP005121.1, AC079089.1, AC104117.3, and SOX1-OT) were filtered out by LASSO, and 3 prognostic irlncRNAs were applied to construct a model. Among these 6 candidate irlncRNAs, ELFN1-AS1 was considered as an oncogene in a variety of cancers, AC079089.1, AC104117.3, and SOX1-OT have been shown to have a function in the progression of various diseases, and other lncRNAs were first identified [23–28]. The Kaplan–Meier analysis, ROC analysis, Cox regression analysis, C-index, DCA, PCA analysis, and nomogram were applied to assess the validity and accuracy of the risk model. The results of GO and KEGG enrichment analyses indicated that the model is significantly related to immune-related pathways and molecules.

We found that CD4+ T cells, CD8+ T cells, NK cells, M1 macrophages, M2 macrophages, myeloid dendritic cells, and fibroblasts were more abundant in the high-risk group. Significantly, high lymphocytic infiltration in um is associated with poor prognosis, in agreement with our results [23–29]. We also found that the expression of most ICIs-related molecules and the scores of most immune functions were higher in the high-risk group than low-risk group. Also, TIDE scores were higher in the high-risk group than in the low-risk group. TIDE is a computational platform for immunotherapy prediction, and its predictive capabilities have been demonstrated in many cancers with great success [30–33]. These results suggest that patients in the high-risk group have a higher response rate to immunotherapy. Therefore, we believe that patients in the high-risk group might be more appropriate to receive immunotherapy. We also discovered that various drugs (AMG.706, Bicalutamide, BX.795, etc.) were identified for significant differences in the IC50 between different groups. Currently, the immune pathogenesis of UM related to immune cells, cytokines, etc., has been further understood, but they are still unclear, and no definite and effective immunotherapeutic drug has been developed so far. The immune cells and cytokines associated with UM are still unclear.Although immunotherapy with anti-CTLA4 and anti-PD-l/PD-Ll reagents has significantly improved the treatment of metastatic cutaneous melanoma, the application in UM has not been satisfactory [34]. In addition, how to better increase Ml-type TAM, promote DC maturation, and how to suppress NKT cells and activate NK cells, improve DC vaccine, etc., also need to be studied. With the increasing research in basic immunology and ophthalmology, NK cell activation, DC vaccine, and T cell relay therapy have been improved. The immunopathogenesis of UM and the related disciplines such as basic immunology and ophthalmology are developing rapidly. We believed that the research on the immunopathogenesis and immunotherapy of UM will make new breakthroughs in the future.

### 4.1. Limitations

Naturally, this study has some drawbacks and limitations. On the one hand, the UM samples extracted from the TCGA consisted of only 80 tumor samples and no normal samples, which was small and did not allow for differential expression analysis. On the other hand, the model developed in this study lacks validation by cellular experiments, animal experiments, or clinical samples. In subsequent studies, we will further expand the tumor samples, collect as many normal samples as possible, and conduct relevant experiments to follow-up our experimental findings.

## 5. Conclusions

Taken together, we constructed a new model on the basis of irlncRNAs that can precisely predict prognosis and response on immunotherapy of UM patients, which may provide worthwhile clinical applications in antitumor immunotherapy.

## Data Availability

The original contributions presented in the study are publicly available. These data can be found at https://portal.gdc.cancer.gov (TCGA-UM).

## Supplementary Materials

Supplementary Materials Figure S1: (a) Kaplan–Meier survival curve, the expression of the 3 prognostic irlncRNAs, patterns of survival outcome, and distribution of risk score for patients between different groups in the training set. (b) Kaplan–Meier survival curve, the expression of the 3 prognostic irlncRNAs, patterns of survival outcome, and distribution of risk score for patients between different groups in the testing set. Figure S2: (a, b): the ROC curves demonstrated the high sensitivity and specificity of the signature for survival prediction, and the one-, three-, and five-year AUC values, respectively, were 0.967, 0.886, and 0.964 in the testing set and 0.974, 0.924, and 0.939 in the training set. (c) The calibration plot of the nomogram predicting the probability of the one-, three-, and five-year prognosis. Figure S3: identification of potential drugs targeting the model (*P* < 0.05). Table S1: identified 409 prognostic irlncRNAs. Table S2: the baseline features of these datasets, demonstrating no statistically significant variations in clinical features (*p* > 0.05). Table S3: original data of GO. Table S4: original data of KEGG. .
