# Supplementary material for: Identification of Immune-Related lncRNAs for Predicting Prognosis and Immune Landscape Characteristics of Uveal Melanoma
Source: J Oncol. 2022 Aug 29;2022:7680657. doi: 10.1155/2022/7680657 (PMC9668462; doi:10.1155/2022/7680657)
Supplement: Supplementary Materials — Figure S1: (a) Kaplan–Meier survival curve, the expression of the 3 prognostic irlncRNAs, patterns of survival outcome, and distribution of risk score for patients between different groups in the training set. (b) Kaplan–Meier survival curve, the expression of the 3 prognostic irlncRNAs, patterns of survival outcome, and distribution of risk score for patients between different groups in the testing set. Figure S2: (a, b): the ROC curves demonstrated the high sensitivity and specificity of the signature for survival prediction, and the one-, three-, and five-year AUC values, respectively, were 0.967, 0.886, and 0.964 in the testing set and 0.974, 0.924, and 0.939 in the training set. (c) The calibration plot of the nomogram predicting the probability of the one-, three-, and five-year prognosis. Figure S3: identification of potential drugs targeting the model (P < 0.05). Table S1: identified 409 prognostic irlncRNAs. Table S2: the baseline features of these datasets, demonstrating no statistically significant variations in clinical features (p > 0.05). Table S3: original data of GO. Table S4: original data of KEGG. . [file 7680657.f1.zip › 7680657.f1/Supplementary Table1.pdf]

id	HR	HR.95L	HR.95H	pvalue
MIR4458HC	1.89822	1.042512	3.456306	0.036071
LINC0090C	3.973997	1.383628	11.41394	0.010372
AC064807.	2.937122	1.183049	7.291913	0.020218
BX293535.	0.379108	0.163627	0.878359	0.023666
AC010463.	0.280577	0.094941	0.829178	0.021517
AC083798.	0.380013	0.16099	0.897011	0.027245
ZBTB11-AS	0.38639	0.157432	0.948329	0.037915
AC027307.	0.162814	0.047199	0.561638	0.004065
AC064807.	2.559874	1.003514	6.530007	0.049148
AC135050.	5.063511	1.245924	20.57841	0.023371
RFX3-AS1	2.097332	1.190072	3.696247	0.010412
AC147067.	2.360516	1.169919	4.762753	0.016478
LINC02611	1.725554	1.045464	2.848052	0.032853
AC004816.	0.256016	0.088818	0.737958	0.011651
LINC01762	0.185914	0.044528	0.776242	0.021037
SNHG15	4.327839	1.35344	13.83895	0.013501
AC009812.	1.856999	1.208782	2.852828	0.00472
AC005962.	0.106173	0.01893	0.595514	0.0108
AP000238.	1.925881	1.286947	2.882028	0.00144
MAN1B1-DT	0.232926	0.086316	0.628561	0.004019
LINC01752	2.881706	1.067528	7.778932	0.036713
FGF14-AS2	0.245776	0.105279	0.573769	0.001178
PVT1	3.59411	1.588824	8.130306	0.002129
SH3PXD2A-	1.751339	1.052642	2.913799	0.030968
AC036214.	2.940505	1.595353	5.419847	0.000546
SEPSECS-A	4.013813	1.083205	14.87317	0.037566
AC009812.	2.976987	1.1793	7.515008	0.020941
AC010273.	0.279498	0.084034	0.929614	0.037618
AC009065.	6.715061	2.4644	18.29737	0.000196
AC103563.	0.45247	0.262405	0.780204	0.004333
AC131235.	0.124792	0.027048	0.575765	0.007639
AL354836.	3.644694	1.447356	9.177973	0.006058
AL391840.	0.200655	0.042908	0.938356	0.04127
MAILR	2.743651	1.092161	6.89241	0.03175
AC040977.	0.221467	0.091861	0.533936	0.000786
AC007637.	4.336512	1.829225	10.2805	0.000865
AC135048.	0.234563	0.084549	0.650738	0.005349
AL021392.	6.163883	2.159139	17.59658	0.000679
AP000892.	0.375426	0.160379	0.878818	0.023968
SSBP3-AS1	3.543168	1.588532	7.902919	0.001997
EMSLR	2.439869	1.100917	5.407273	0.028038
LINC0260C	0.353236	0.135182	0.92302	0.033719
LINC0127C	3.945138	1.6647	9.349503	0.001823
AC104137.	2.973508	1.281546	6.899281	0.011161
AL137067.	2.202825	1.087712	4.461144	0.028273
LINC02367	0.393709	0.200887	0.771613	0.006624
Z69706.1	0.359116	0.149436	0.863008	0.022061
AC124798.	2.654921	1.547618	4.554487	0.000391
AC011363.	2.256471	1.116901	4.55874	0.023324
AL355596.	0.657316	0.442976	0.975366	0.037175
AC010731.	1.825606	1.127639	2.955588	0.014339
AC087276.	2.422894	1.003361	5.850747	0.049134
AL157871.	1.454612	1.101685	1.920598	0.008218

MIR155HG	1.905768	1.21955	2.978107	0.004634
PAX8-AS1	2.103496	1.142011	3.874478	0.01703
LINC01521	0.361842	0.135911	0.963343	0.041879
AC100791.	2.06045	1.257498	3.376113	0.004113
NOP53-AS1	0.33965	0.137263	0.840449	0.019492
AL596244.	0.315398	0.122483	0.812164	0.016799
AL133520.	3.513816	1.03873	11.88655	0.043272
AC079848.	0.352662	0.152948	0.813153	0.014476
AL691432.	0.452596	0.214254	0.956075	0.037738
AC040160.	0.007723	0.000317	0.187882	0.00282
Z93930.2	5.436121	1.501687	19.67881	0.009897
AC068025.	0.127608	0.026042	0.625306	0.011117
KRTAP5-AS	0.120164	0.029084	0.496461	0.003418
NCKAP5-AS	0.367554	0.177964	0.759119	0.006836
AP003469.	2.78664	1.141729	6.801407	0.024379
LAMC1-AS1	3.184692	1.246576	8.136093	0.015498
AC104794.	0.240556	0.078413	0.737974	0.01273
AC156455.	1.661176	1.03784	2.658893	0.034454
AC084018.	1.700781	1.028243	2.813201	0.038599
AL121574.	0.355969	0.140286	0.903254	0.029695
AL606469.	0.119364	0.024091	0.591423	0.009236
SYN3-AS1	0.30358	0.116172	0.793313	0.014999
AC145124.	0.307965	0.111187	0.852999	0.023461
RNF207-AS	0.572979	0.330616	0.993011	0.047149
BAIAP2-DT	0.164044	0.045005	0.597945	0.006158
LINC0208	1.809319	1.09697	2.984252	0.020208
C8orf44	2.026024	1.150727	3.567115	0.014429
AC022007.	0.24782	0.074458	0.824817	0.022973
AC245297.	3.571005	1.382963	9.220838	0.008542
AC026356.	4.396631	1.404572	13.76247	0.010975
AC108075.	2.250349	1.223399	4.139344	0.009097
AC009509.	2.841461	1.062859	7.596404	0.037391
AC008555.	0.279033	0.117777	0.661073	0.003726
AC021242.	0.16325	0.031248	0.852873	0.031664
GAPLINC	2.339198	1.139822	4.800615	0.020517
SNHG7	0.4409	0.252455	0.770013	0.003994
ELFN1-AS1	1.759393	1.28659	2.405943	0.000403
AC100803.	2.5549	1.273826	5.124338	0.008253
AC008555.	0.052433	0.005428	0.506526	0.010842
SOCS2-AS1	2.310434	1.166411	4.576519	0.016334
AL589986.	3.559399	1.258986	10.06312	0.016652
AC079015.	1.88965	1.110403	3.215748	0.018975
ARIH2OS	0.377128	0.175675	0.809592	0.012354
LINC0049	2.728606	1.217594	6.114758	0.014762
ALDH1L1-A	0.064939	0.004633	0.910144	0.042371
GSEC	1.915588	1.00226	3.661204	0.049207
AL139384.	4.53905	1.18782	17.3452	0.026994
SNHG18	0.47068	0.299781	0.739006	0.00106
AC022784.	0.369849	0.150381	0.909614	0.030289
AC132872.	0.370357	0.15352	0.893463	0.027058
AC136475.	2.488692	1.410535	4.39095	0.001648
AC137894.	0.493522	0.245862	0.990653	0.046991
AL591845.	4.639962	1.622888	13.26601	0.004192
AF131216.	0.018303	0.001566	0.21392	0.001426

EPB41L4A-	0.408003	0.190338	0.874585	0.021199
BAALC-AS1	0.227832	0.063176	0.821637	0.023812
LINC0102	0.436966	0.196565	0.971383	0.042235
AL049840.	2.760483	1.020965	7.463788	0.045409
AC107464.	0.405244	0.193283	0.849647	0.016788
AC103974.	0.233994	0.099901	0.548074	0.000824
TONSL-AS1	2.670799	1.316816	5.416979	0.006474
SLC25A21-	0.304987	0.126284	0.73657	0.0083
AC092368.	0.070377	0.01226	0.403984	0.002915
SCGB1B2P	0.266018	0.105805	0.668827	0.004877
AC069185.	0.063281	0.010527	0.380398	0.00256
AC022364.	0.175543	0.040072	0.768996	0.020973
TBX2-AS1	0.36281	0.166562	0.790284	0.010695
ADAMTSL4-	3.054446	1.086146	8.589676	0.034293
AL049838.	0.584939	0.352194	0.971493	0.038294
AC009902.	2.695929	1.290604	5.631501	0.008322
AC087521.	0.279637	0.082873	0.943573	0.040018
AC016737.	0.275703	0.096824	0.785052	0.015811
MAP3K14-A	4.966403	1.057087	23.33315	0.042327
GAS1RR	0.189353	0.041249	0.869214	0.032336
AL138921.	2.66367	1.210854	5.859614	0.014867
MICB-DT	2.114169	1.230862	3.631365	0.006677
AL162377.	0.219866	0.07036	0.687057	0.009171
LINC0040	2.376006	1.459058	3.869213	0.000504
AC009495.	1.785574	1.038668	3.069581	0.035975
LINC02761	0.384619	0.201413	0.734468	0.003792
LINC01971	5.345702	2.236117	12.77953	0.000163
DGCR11	2.734234	1.071846	6.974916	0.035276
AC084855.	0.168792	0.030455	0.935503	0.041723
PELATON	2.201812	1.175531	4.12407	0.0137
AL022341.	0.160879	0.041334	0.626165	0.00841
PRRT3-AS1	0.338519	0.173967	0.658715	0.001427
MIR22HG	1.714431	1.033774	2.843247	0.036739
LINC0166	1.990992	1.170672	3.386131	0.011037
LINC0062	2.346662	1.114729	4.940054	0.024708
AP005121.	2.325137	1.528898	3.536049	7.99E-05
ACVR2B-AS	0.168632	0.045646	0.622981	0.007591
AP003390.	5.745149	1.948461	16.9399	0.00153
TIPARP-AS	0.231402	0.070867	0.755596	0.015345
AC009065.	7.758614	2.140661	28.12033	0.001818
ZNF30-AS1	0.107363	0.018381	0.627102	0.013205
AC091544.	2.37634	1.094717	5.158404	0.028611
NAMA	2.871305	1.362389	6.051422	0.005555
AC008840.	3.221592	1.122166	9.248773	0.029692
CHL1-AS2	0.186461	0.040365	0.861339	0.031465
AC087501.	6.504151	1.666921	25.37852	0.007027
LINC0119	0.291702	0.105795	0.804291	0.017273
AC079089.	4.789472	2.221023	10.32814	6.46E-05
AC005840.	4.024645	1.851079	8.750446	0.000442
POLR2J4	6.554007	1.799585	23.8694	0.004359
AC104117.	0.155156	0.042787	0.562626	0.004582
YTHDF3-AS	3.013091	1.324248	6.855757	0.008551
LINC0100	0.078928	0.018396	0.338632	0.000633
AL162457.	1.67165	1.116988	2.501738	0.012497

AL354892.	0.235119	0.105938	0.521823	0.000372
AC068888.	3.449714	1.1919	9.9845	0.022388
AF186192.	0.098907	0.013002	0.752409	0.025434
AC021321.	2.479862	1.056619	5.820183	0.036934
AC104825.	0.411734	0.19627	0.863733	0.0189
LINC0035	18.02579	3.334111	97.45596	0.000784
AC022148.	0.25436	0.080532	0.803395	0.019647
MAP4K3-DT	2.168378	1.266277	3.71314	0.0048
AL109976.	3.616217	1.197174	10.92325	0.022665
GAS6-AS1	1.540883	1.079239	2.199996	0.017328
AC004943.	0.219593	0.060559	0.796263	0.021077
AC018529.	2.055722	1.290683	3.274229	0.00241
PINK1-AS	0.207517	0.048639	0.885362	0.033632
AC010761.	2.269803	1.054563	4.885443	0.0361
AC105942.	0.448842	0.220793	0.912435	0.026889
AC116025.	2.243494	1.090393	4.616011	0.02816
AC015818.	2.759436	1.446717	5.263289	0.002064
AC135178.	0.296766	0.090552	0.972595	0.044873
LINC0184	2.908665	1.416418	5.973049	0.003635
TMPO-AS1	5.007298	1.063697	23.57158	0.041541
BX649632.	0.196994	0.039511	0.982168	0.047489
AL157702.	1.719701	1.111679	2.660275	0.014868
AC100810.	0.220243	0.066389	0.730655	0.013404
AL391988.	2.300823	1.199629	4.412851	0.012151
AL162388.	0.011499	0.000171	0.771033	0.03742
AL589843.	0.208831	0.071142	0.613003	0.004362
LINC0207	2.602962	1.056403	6.413665	0.037597
AP000808.	0.158449	0.035716	0.702938	0.015364
AC011498.	0.193338	0.044986	0.83092	0.027179
WWC2-AS2	0.233457	0.064568	0.844101	0.026527
MGC16275	0.246533	0.097592	0.622783	0.003061
TFAP2A-AS	0.496274	0.265856	0.926394	0.027804
AL136368.	0.296254	0.121921	0.719863	0.00724
AC093799.	2.007892	1.171417	3.44167	0.011231
AC000067.	2.706351	1.280977	5.717773	0.009085
AC009878.	2.100879	1.038538	4.249908	0.038908
TBC1D8-AS	0.213113	0.053546	0.848182	0.028264
ENTPD3-AS	0.073971	0.013757	0.397746	0.002412
AC005332.	1.809087	1.098876	2.978311	0.019772
AL590235.	1.914644	1.02181	3.587613	0.042631
AC092117.	0.248444	0.079105	0.780286	0.017086
AL121957.	0.295032	0.095248	0.913867	0.034336
AL137077.	0.251064	0.092338	0.682635	0.006767
OTUD6B-AS	1.873916	1.078964	3.254568	0.02576
SNHG6	4.341603	1.335705	14.11203	0.014637
CEBPA-DT	0.151643	0.03764	0.610944	0.007977
AC017104.	1.715408	1.081171	2.721703	0.021944
AC009812.	2.519306	1.308558	4.850303	0.005699
AC015909.	0.400634	0.176586	0.90895	0.028644
LINC0063	0.235728	0.066162	0.839877	0.025804
AL021707.	0.107748	0.020285	0.572329	0.008925
SNHG32	0.575332	0.368699	0.897769	0.014892
AC008875.	1.923144	1.087981	3.399398	0.024443
AL133342.	4.021202	1.112132	14.5397	0.033835

AC023983.	3.837756	1.348761	10.91992	0.011711
AC131011.	2.528736	1.271634	5.028573	0.008166
AC016590.	0.180006	0.041385	0.78295	0.022243
AL606760.	0.054064	0.006862	0.425969	0.005601
PKNOX2-AS	0.248458	0.061764	0.999473	0.049913
LINC0026E	2.771667	1.124705	6.830356	0.026736
AC026369.	3.048414	1.350939	6.878792	0.007266
AC012213.	2.170332	1.108514	4.249239	0.02379
AC069200.	3.979429	1.343909	11.78343	0.012644
AC011978.	2.549698	1.386921	4.687333	0.002588
AP001021.	2.172187	1.125379	4.192717	0.020777
AC226118.	2.412347	1.049764	5.54355	0.038045
AC124016.	0.322825	0.119603	0.87135	0.025629
AP005233.	1.563254	1.104346	2.21286	0.011744
LINC0096E	2.013578	1.188291	3.41204	0.009293
AC073073.	3.436814	1.30232	9.069728	0.01265
AC005523.	0.192864	0.043302	0.858993	0.03082
AL139289.	0.490992	0.245798	0.980776	0.04391
ZNF571-AS	0.258807	0.094937	0.705535	0.008251
AC138207.	0.170484	0.053589	0.542369	0.002735
MIR5689HC	0.172479	0.037612	0.790937	0.023709
AC018529.	1.613596	1.174192	2.217433	0.003177
AC133552.	0.388403	0.157537	0.957599	0.039969
AC007786.	0.140181	0.022796	0.862041	0.033993
AC139887.	5.335746	1.368465	20.80447	0.015874
AL121603.	14.19344	2.130997	94.53493	0.006107
LINC00622	1.887969	1.01035	3.527914	0.046344
AC009121.	0.150258	0.028837	0.782948	0.024418
AC006157.	0.16282	0.03139	0.844543	0.030685
AC016876.	0.213994	0.075687	0.605037	0.003643
SPINT1-AS	0.38308	0.172687	0.849805	0.01826
AL031289.	3.573829	1.19775	10.66353	0.022402
AP002387.	0.459583	0.215982	0.977934	0.043604
AL157904.	0.304433	0.097725	0.948365	0.040227
AC025265.	2.136252	1.258775	3.625408	0.004912
AC114341.	0.533625	0.285502	0.997388	0.04905
AC090912.	2.440671	1.153903	5.162371	0.01957
AC012442.	0.095771	0.024316	0.377202	0.000797
AC040970.	2.319417	1.237396	4.347594	0.008679
AP000911.	0.258955	0.073002	0.918575	0.036489
LINC0261C	0.482689	0.264136	0.882079	0.017891
AL022322.	1.83632	1.066885	3.160672	0.028261
TAF1A-AS1	0.251451	0.085633	0.738349	0.012008
AC105020.	5.173517	1.405492	19.04335	0.013439
SOX1-OT	3.408121	1.788499	6.494435	0.000194
AP001007.	0.55438	0.382998	0.802451	0.00177
KDM7A-DT	2.634906	1.318385	5.266085	0.0061
LINC02298	5.52845	1.533304	19.93327	0.00897
AC005070.	5.269944	1.634591	16.99037	0.005391
AC092279.	3.69684	1.057855	12.91919	0.040554
AL391244.	0.307769	0.123007	0.770052	0.011789
DCST1-AS1	2.762264	1.059318	7.202843	0.037727
SNHG29	0.526614	0.306781	0.903975	0.02001
AC005759.	0.035676	0.003254	0.391196	0.00637

AP000894.	0.465116	0.251391	0.860543	0.014752
LINC01857	2.53258	1.322771	4.848883	0.005046
RAP2C-AS1	0.198315	0.045715	0.860314	0.030701
SH3RF3-AS	3.547068	1.557796	8.076599	0.002563
AC009495.	1.988757	1.053067	3.755844	0.03406
AC010503.	0.303488	0.113354	0.812547	0.01764
LINC01954	2.616949	1.138924	6.013064	0.023425
AATBC	1.450133	1.002239	2.098188	0.048629
AC092053.	0.257547	0.074398	0.891559	0.032263
PSPC1-AS2	2.131527	1.058925	4.290587	0.033975
VLDLR-AS1	3.88656	1.190003	12.69353	0.024574
AC242842.	2.532821	1.148394	5.586221	0.021289
SNHG11	6.255974	1.027197	38.10097	0.046693
RAMP2-AS1	0.193135	0.054769	0.681063	0.010548
AL354696.	3.045541	1.154295	8.035484	0.024459
KIF1C-AS1	3.792492	1.079042	13.32941	0.037655
AC010969.	2.328087	1.007232	5.381074	0.048062
HRAT92	2.068354	1.114127	3.839858	0.021317
LINC00665	0.322183	0.110877	0.936195	0.037424
DOCK9-DT	0.420778	0.187504	0.944267	0.035815
AC124045.	0.121955	0.027571	0.539446	0.005545
AC092818.	0.012141	0.000292	0.504965	0.020385
AC012073.	4.351444	1.22383	15.47198	0.023084
RASGRP3-A	2.741445	1.235291	6.08401	0.013157
L3MBTL4-A	2.297036	1.370941	3.848726	0.001588
LHFPL3-AS	2.715444	1.342337	5.493131	0.005453
AC069148.	3.11209	1.273635	7.604302	0.012753
AC090198.	1.951948	1.113242	3.422528	0.019576
AL049874.	0.12527	0.024256	0.646952	0.013144
AC100814.	1.825392	1.104767	3.016071	0.018832
AC127070.	2.812895	1.005817	7.866614	0.048722
AC016747.	2.806325	1.370098	5.748097	0.004792
AC005740.	0.245019	0.061725	0.972608	0.04556
AC010834.	3.122316	1.062783	9.172952	0.038386
MACORIS	1.93738	1.151453	3.259741	0.012732
AC108053.	2.058138	1.190457	3.558241	0.009763
AL157395.	6.505566	1.445399	29.28077	0.01469
HHATL-AS1	0.210598	0.045723	0.970002	0.045603
LINC00689	0.412224	0.195679	0.868404	0.019747
AP003352.	2.114845	1.017409	4.396038	0.044835
AF117829.	2.621155	1.028757	6.678402	0.043448
PLBD1-AS1	4.456926	1.536427	12.92882	0.005954
STK32A-AS	3.071327	1.366177	6.904709	0.00663
AC009063.	0.083648	0.00866	0.807967	0.032013
AP002954.	3.332949	1.175381	9.451016	0.023584
AC097359.	0.225704	0.075646	0.673432	0.007612
AC104129.	2.001964	1.348337	2.972444	0.000577
LINC02021	0.283287	0.0899	0.892671	0.031252
AC046168.	0.678127	0.476799	0.964467	0.030674
PSMB8-AS1	1.477635	1.047784	2.083832	0.026008
LINC01637	4.261428	1.743043	10.41843	0.001482
AC026803.	0.211698	0.052342	0.856213	0.029428
AC112484.	0.242231	0.061228	0.958312	0.043316
AC012236.	3.798147	1.191939	12.1029	0.024014

AL117336.	2.717262	1.119237	6.596916	0.027183
PRR34-AS1	4.261593	1.585793	11.45242	0.004051
LINC0169[illegible]	1.81E-14	1.62E-27	0.202225	0.038995
LINC0115[illegible]	0.26769	0.081712	0.876953	0.029493
LINC0232[illegible]	2.012665	1.082232	3.743025	0.027132
PP7080	1.681487	1.008815	2.802692	0.046192
AC012645.	0.179617	0.044048	0.732443	0.016659
LINC0051[illegible]	0.554842	0.380622	0.808807	0.002188
ZNF561-A[illegible]	0.124727	0.025971	0.59901	0.00932
PRKAR1B-[illegible]	3.123331	1.406652	6.935048	0.005137
AC018904.	2.561538	1.457474	4.501951	0.001078
AC090204.	0.673047	0.466589	0.970857	0.034159
AL590062.	0.451881	0.205863	0.991903	0.047678
SBNO1-AS1	0.4235	0.188334	0.952308	0.037695
AP002907.	2.157414	1.063454	4.376716	0.033137
AC025442.	3.696656	1.215677	11.24087	0.021214
AP003721.	0.225602	0.053994	0.942636	0.041257
AC002550.	4.306716	1.260294	14.71704	0.019861
AC034229.	4.477355	1.512829	13.25114	0.006774
AC008558.	0.253684	0.074495	0.863887	0.028236
SNHG8	0.349493	0.165392	0.738518	0.005887
LINC0127[illegible]	0.043224	0.005942	0.314437	0.001918
MYCNUT	1.74267	1.116734	2.719446	0.014436
ZNF793-A[illegible]	0.521635	0.301948	0.901158	0.019644
HDHD5-AS1	0.258034	0.082752	0.804593	0.019561
AP001628.	1.705699	1.096453	2.653474	0.017866
LINC0190[illegible]	2.394498	1.223783	4.685163	0.010783
CFAP58-D[illegible]	0.404158	0.199029	0.820704	0.012187
AC009902.	4.274394	1.787431	10.22162	0.001092
AC099661.	0.147976	0.030501	0.717913	0.017728
ZNF582-A[illegible]	0.313615	0.136477	0.720668	0.006302
AC017028.	1.881374	1.107586	3.195749	0.019389
LINC02861	4.402285	1.691899	11.45465	0.002384
AC012313.	0.206465	0.051069	0.834712	0.026867
CD2BP2-D[illegible]	2.454418	1.005438	5.991587	0.048624
AC008105.	3.125681	1.379173	7.083866	0.006332
CENATAC-[illegible]	0.362768	0.1592	0.826638	0.01582
AL731571.	3.664998	1.283266	10.46721	0.015276
KTN1-AS1	0.214432	0.047568	0.966645	0.045057
ZNF667-A[illegible]	0.292395	0.132721	0.644166	0.002279
AP003120.	2.664514	1.171099	6.062371	0.019464
AC020916.	2.874813	1.185553	6.971046	0.019461
LINC0108[illegible]	2.19741	1.096106	4.405239	0.026517
AP000223.	0.139632	0.043753	0.445613	0.000884
AP003071.	0.173782	0.036934	0.81769	0.026781
AC103706.	2.981339	1.510459	5.884558	0.00164
AC026748.	0.109227	0.029883	0.399241	0.000813
CARD8-AS1	2.42312	1.154424	5.086098	0.019306
DLGAP1-A[illegible]	2.162986	1.111724	4.208337	0.023096
AL138966.	5.929072	1.003027	35.0478	0.049612
AC126468.	2.55739	1.48629	4.400384	0.000696
AL139384.	0.324186	0.108608	0.967673	0.043502
AC093620.	3.936669	1.449216	10.69362	0.007196
PCAT6	0.429444	0.220188	0.837569	0.013137

AC117422.	0.026115	0.001178	0.578852	0.021123
SOS1-IT1	1.981541	1.158838	3.388312	0.01247
AC015909.	0.563207	0.325454	0.974646	0.040195
AL135905.	0.490192	0.253998	0.946024	0.033555
AL451069.	2.120541	1.295658	3.470588	0.002786
AC016738.	3.008247	1.093363	8.2768	0.03294
AL512408.	0.069417	0.009378	0.513833	0.009004
AC112722.	1.695492	1.007481	2.853348	0.046809
PDCD4-AS1	0.287769	0.127395	0.650035	0.002736
AC008906.	2.780215	1.075599	7.186319	0.034826
BANCR	1.269499	1.012817	1.591233	0.038409
HCP5	1.304105	1.03241	1.647301	0.02591
AC012313.	0.293304	0.121014	0.710887	0.006619
WASHC5-AS	3.09519	1.322196	7.245672	0.009226
AC034198.	0.190049	0.056364	0.640812	0.007416
AL365434.	0.260837	0.108494	0.627093	0.002676
AL031651.	3.611862	1.425207	9.153439	0.006794
AC092535.	2.438938	1.478554	4.023134	0.000481
AL078590.	1.707001	1.044023	2.790985	0.033031
ADIRF-AS1	2.494427	1.062438	5.856499	0.035813
AL136162.	0.352021	0.143735	0.862132	0.022338
AC011388.	0.425943	0.201529	0.900251	0.025406
AC005899.	2.970649	1.045105	8.443894	0.041079
AP003419.	6.077016	1.751525	21.08456	0.004469
AL138885.	0.570777	0.379332	0.858842	0.007147
AC117500.	0.341194	0.144248	0.807033	0.014363
AL391422.	0.391326	0.190165	0.805278	0.01083
PRKAR2A-A	0.062861	0.008155	0.484523	0.007922
AC006435.	2.044116	1.183265	3.531254	0.010369
AL022162.	0.510226	0.27064	0.961909	0.037525
AC110285.	0.553547	0.310957	0.985391	0.044433
AC022916.	0.248165	0.074558	0.826018	0.023117
