# Supplementary material for: Identification of Immune-Related lncRNAs for Predicting Prognosis and Immune Landscape Characteristics of Uveal Melanoma
Source: J Oncol. 2022 Aug 29;2022:7680657. doi: 10.1155/2022/7680657 (PMC9668462; doi:10.1155/2022/7680657)
Supplement: Supplementary Materials — Figure S1: (a) Kaplan–Meier survival curve, the expression of the 3 prognostic irlncRNAs, patterns of survival outcome, and distribution of risk score for patients between different groups in the training set. (b) Kaplan–Meier survival curve, the expression of the 3 prognostic irlncRNAs, patterns of survival outcome, and distribution of risk score for patients between different groups in the testing set. Figure S2: (a, b): the ROC curves demonstrated the high sensitivity and specificity of the signature for survival prediction, and the one-, three-, and five-year AUC values, respectively, were 0.967, 0.886, and 0.964 in the testing set and 0.974, 0.924, and 0.939 in the training set. (c) The calibration plot of the nomogram predicting the probability of the one-, three-, and five-year prognosis. Figure S3: identification of potential drugs targeting the model (P < 0.05). Table S1: identified 409 prognostic irlncRNAs. Table S2: the baseline features of these datasets, demonstrating no statistically significant variations in clinical features (p > 0.05). Table S3: original data of GO. Table S4: original data of KEGG. . [file 7680657.f1.zip › 7680657.f1/Supplementary Table2.pdf]

Covariate	Type	Total	Test	Train	Pvalue
Age	<=65	80(100%)	40(100%)	40(100%)	1
Gender	FEMALE	35(43.75%	19(47.5%)	16(40%)	0.6522
Gender	MALE	45(56.25%	21(52.5%)	24(60%)	
Stage	stage II	39(48.75%	23(57.5%)	16(40%)	0.2752
Stage	stage III	37(46.25%	15(37.5%)	22(55%)	
Stage	stage IV	4(5%)	2(5%)	2(5%)	
T	T2	14(17.5%)	9(22.5%)	5(12.5%)	0.4463
T	T3	32(40%)	16(40%)	16(40%)	
T	T4	34(42.5%)	15(37.5%)	19(47.5%)	
M	M0	73(91.25%	37(92.5%)	36(90%)	1
M	M1	3(3.75%)	1(2.5%)	2(5%)	
M	unknow	4(5%)	2(5%)	2(5%)	
N	N0	76(95%)	38(95%)	38(95%)	1
N	unknow	4(5%)	2(5%)	2(5%)	
