# Supplementary material for: Identification of Immune-Related lncRNAs for Predicting Prognosis and Immune Landscape Characteristics of Uveal Melanoma
Source: J Oncol. 2022 Aug 29;2022:7680657. doi: 10.1155/2022/7680657 (PMC9668462; doi:10.1155/2022/7680657)
Supplement: Supplementary Materials — Figure S1: (a) Kaplan–Meier survival curve, the expression of the 3 prognostic irlncRNAs, patterns of survival outcome, and distribution of risk score for patients between different groups in the training set. (b) Kaplan–Meier survival curve, the expression of the 3 prognostic irlncRNAs, patterns of survival outcome, and distribution of risk score for patients between different groups in the testing set. Figure S2: (a, b): the ROC curves demonstrated the high sensitivity and specificity of the signature for survival prediction, and the one-, three-, and five-year AUC values, respectively, were 0.967, 0.886, and 0.964 in the testing set and 0.974, 0.924, and 0.939 in the training set. (c) The calibration plot of the nomogram predicting the probability of the one-, three-, and five-year prognosis. Figure S3: identification of potential drugs targeting the model (P < 0.05). Table S1: identified 409 prognostic irlncRNAs. Table S2: the baseline features of these datasets, demonstrating no statistically significant variations in clinical features (p > 0.05). Table S3: original data of GO. Table S4: original data of KEGG. . [file 7680657.f1.zip › 7680657.f1/Supplementary Table3.pdf]

ONTOLOGY	ID	Descripti	GeneRati	BgRatio	pvalue	p.adjust	qvalue	geneID	Count
BP	GO:00421	T cell a	111/1266	487/1872	6.59E-31	3.59E-27	3.07E-27	GPR183/T	111
BP	GO:00508	regulati	78/1266	329/1872	4.09E-23	1.12E-19	9.53E-20	CD70/CTN	78
BP	GO:00071	leukocyt	82/1266	371/1872	3.95E-22	7.18E-19	6.14E-19	CD70/HLA-	82
BP	GO:19030	regulati	75/1266	336/1872	1.33E-20	1.82E-17	1.55E-17	CD70/HLA-	75
BP	GO:00508	positive	58/1266	216/1872	2.79E-20	3.04E-17	2.60E-17	CD70/HLA-	58
BP	GO:00506	regulati	59/1266	225/1872	4.69E-20	3.95E-17	3.38E-17	GPR183/C	59
BP	GO:00024	leukocyt	87/1266	440/1872	5.07E-20	3.95E-17	3.38E-17	TCIRG1/C	87
BP	GO:00329	regulati	59/1266	227/1872	7.52E-20	5.13E-17	4.38E-17	GPR183/C	59
BP	GO:00224	regulati	87/1266	448/1872	1.71E-19	1.04E-16	8.88E-17	CD70/HLA-	87
BP	GO:19030	positive	60/1266	239/1872	2.34E-19	1.28E-16	1.09E-16	CD70/HLA-	60
BP	GO:00706	regulati	60/1266	245/1872	8.66E-19	4.30E-16	3.67E-16	GPR183/C	60
BP	GO:00224	positive	65/1266	284/1872	1.32E-18	5.98E-16	5.11E-16	CD70/HLA-	65
BP	GO:00480	antigen	29/1266	62/18723	4.04E-18	1.70E-15	1.45E-15	HLA-A/TA	29
BP	GO:00508	positive	81/1266	420/1872	4.91E-18	1.91E-15	1.63E-15	GPR183/L	81
BP	GO:00026	positive	79/1266	409/1872	1.17E-17	4.25E-15	3.63E-15	GPR183/L	79
BP	GO:00018	positive	85/1266	467/1872	2.94E-17	1.00E-14	8.57E-15	LRRK2/CD	85
BP	GO:19031	mononucl	80/1266	426/1872	4.02E-17	1.29E-14	1.10E-14	GPR183/T	80
BP	GO:00457	positive	81/1266	437/1872	5.63E-17	1.71E-14	1.46E-14	CD70/HLA-	81
BP	GO:00512	positive	72/1266	362/1872	6.58E-17	1.89E-14	1.61E-14	GPR183/C	72
BP	GO:00300	lymphocy	73/1266	374/1872	1.16E-16	3.16E-14	2.70E-14	GPR183/T	73
BP	GO:00466	lymphocy	62/1266	288/1872	1.95E-16	5.07E-14	4.33E-14	GPR183/C	62
BP	GO:00329	mononucl	62/1266	291/1872	3.29E-16	8.16E-14	6.98E-14	GPR183/C	62
BP	GO:00024	lymphocy	69/1266	350/1872	4.77E-16	1.13E-13	9.67E-14	TCIRG1/C	69
BP	GO:00706	leukocyt	65/1266	318/1872	5.43E-16	1.23E-13	1.05E-13	GPR183/T	65
BP	GO:00421	regulati	45/1266	171/1872	1.13E-15	2.46E-13	2.11E-13	CD70/CTN	45
BP	GO:00024	adaptive	68/1266	356/1872	4.09E-15	8.58E-13	7.33E-13	TCIRG1/C	68
BP	GO:00420	T cell p	48/1266	199/1872	5.09E-15	1.03E-12	8.80E-13	CD70/CTN	48
BP	GO:00198	antigen	34/1266	106/1872	5.95E-15	1.16E-12	9.90E-13	HLA-A/WA	34
BP	GO:00706	positive	40/1266	150/1872	2.71E-14	5.09E-12	4.35E-12	GPR183/C	40
BP	GO:00506	positive	38/1266	137/1872	3.03E-14	5.50E-12	4.70E-12	GPR183/C	38
BP	GO:00024	antigen	20/1266	38/18723	3.58E-14	6.30E-12	5.39E-12	HLA-A/TA	20
BP	GO:00329	positive	38/1266	138/1872	3.91E-14	6.66E-12	5.70E-12	GPR183/C	38
BP	GO:00302	T cell d	54/1266	257/1872	5.05E-14	8.35E-12	7.14E-12	GPR183/T	54
BP	GO:00025	peptide	14/1266	18/18723	9.23E-14	1.48E-11	1.27E-11	HLA-DMA/	14
BP	GO:00023	MHC clas	13/1266	16/18723	2.69E-13	4.08E-11	3.49E-11	HLA-DMA/	13
BP	GO:00025	peptide	13/1266	16/18723	2.69E-13	4.08E-11	3.49E-11	HLA-DMA/	13
BP	GO:00023	MHC prot	14/1266	19/18723	3.29E-13	4.85E-11	4.15E-11	HLA-DMA/	14
BP	GO:00421	positive	31/1266	101/1872	3.58E-13	5.13E-11	4.39E-11	CD70/HLA-	31
BP	GO:00198	antigen	21/1266	47/18723	5.27E-13	7.37E-11	6.30E-11	HLA-A/TA	21
BP	GO:00509	leukocyt	65/1266	369/1872	8.32E-13	1.14E-10	9.70E-11	GPR183/A	65
BP	GO:00026	regulati	61/1266	339/1872	1.65E-12	2.20E-10	1.88E-10	BST2/HLA-	61
BP	GO:00026	negative	70/1266	434/1872	8.19E-12	1.06E-09	9.09E-10	BST2/CTN	70
BP	GO:00198	antigen	16/1266	30/18723	1.02E-11	1.30E-09	1.11E-09	HLA-DMA/	16
BP	GO:00024	antigen	13/1266	19/18723	1.08E-11	1.31E-09	1.12E-09	HLA-A/TA	13
BP	GO:00023	leukocyt	52/1266	275/1872	1.08E-11	1.31E-09	1.12E-09	GPR183/L	52
BP	GO:00022	cell act	52/1266	279/1872	1.89E-11	2.25E-09	1.92E-09	GPR183/L	52
BP	GO:00421	B cell a	58/1266	334/1872	2.61E-11	3.03E-09	2.59E-09	GPR183/T	58
BP	GO:00025	antigen	17/1266	36/18723	2.92E-11	3.32E-09	2.83E-09	HLA-DMA/	17
BP	GO:00313	positive	22/1266	63/18723	5.50E-11	6.12E-09	5.24E-09	HLA-A/CY	22
BP	GO:00024	antigen	16/1266	34/18723	1.20E-10	1.31E-08	1.12E-08	HLA-DMA/	16
BP	GO:00019	leukocyt	31/1266	124/1872	1.30E-10	1.39E-08	1.19E-08	HLA-A/CY	31
BP	GO:00027	regulati	44/1266	226/1872	1.57E-10	1.64E-08	1.40E-08	BST2/HLA-	44
BP	GO:00198	antigen	14/1266	26/18723	1.74E-10	1.79E-08	1.53E-08	HLA-A/TA	14

BP	GO:00026[illegible]	negative	39/1266	187/1872[illegible]	2.08E-10	2.10E-08	1.80E-08	LST1/LYN/		39
BP	GO:00313[illegible]	regulati[illegible]	27/1266	99/18723	2.42E-10	2.40E-08	2.05E-08	HLA-A/CY[illegible]		27
BP	GO:00019[illegible]	regulati[illegible]	24/1266	82/18723	4.89E-10	4.76E-08	4.07E-08	HLA-A/CY[illegible]		24
BP	GO:00024[illegible]	T cell m[illegible]	28/1266	109/1872[illegible]	5.15E-10	4.93E-08	4.21E-08	CD70/HLA-		28
BP	GO:00508[illegible]	negative	41/1266	210/1872[illegible]	6.02E-10	5.66E-08	4.84E-08	LST1/LYN/		41
BP	GO:00024[illegible]	antigen [illegible]	14/1266	28/18723	6.36E-10	5.88E-08	5.03E-08	HLA-A/TA[illegible]		14
BP	GO:00975[illegible]	myeloid [illegible]	42/1266	220/1872[illegible]	7.66E-10	6.96E-08	5.95E-08	ANO6/ITG[illegible]		42
BP	GO:00305[illegible]	leukocyt[illegible]	43/1266	230/1872[illegible]	9.50E-10	8.50E-08	7.27E-08	GPR183/A[illegible]		43
BP	GO:00603[illegible]	cell che[illegible]	52/1266	310/1872[illegible]	9.99E-10	8.79E-08	7.52E-08	GPR183/A[illegible]		52
BP	GO:00198[illegible]	antigen [illegible]	11/1266	17/18723	1.10E-09	9.50E-08	8.12E-08	HLA-A/TA[illegible]		11
BP	GO:19021[illegible]	regulati[illegible]	48/1266	279/1872[illegible]	1.87E-09	1.59E-07	1.36E-07	CTNNB1/C[illegible]		48
BP	GO:00019[illegible]	positive	19/1266	56/18723	1.97E-09	1.66E-07	1.42E-07	HLA-A/CY[illegible]		19
BP	GO:00022[illegible]	response	57/1266	363/1872[illegible]	2.03E-09	1.68E-07	1.44E-07	SERPINE1/		57
BP	GO:00022[illegible]	activati[illegible]	58/1266	375/1872[illegible]	2.62E-09	2.13E-07	1.82E-07	HLA-A/LY[illegible]		58
BP	GO:00019[illegible]	cell kil[illegible]	37/1266	188/1872[illegible]	3.27E-09	2.62E-07	2.24E-07	HLA-A/CY[illegible]		37
BP	GO:00301[illegible]	B cell d[illegible]	31/1266	141/1872[illegible]	3.88E-09	3.07E-07	2.62E-07	GPR183/T[illegible]		31
BP	GO:19021[illegible]	positive	33/1266	157/1872[illegible]	4.12E-09	3.17E-07	2.71E-07	CCR1/PIK[illegible]		33
BP	GO:19037[illegible]	positive	33/1266	157/1872[illegible]	4.12E-09	3.17E-07	2.71E-07	CCR1/PIK[illegible]		33
BP	GO:00026[illegible]	positive	42/1266	235/1872[illegible]	6.03E-09	4.57E-07	3.90E-07	HLA-A/CY[illegible]		42
BP	GO:00197[illegible]	B cell m[illegible]	39/1266	210/1872[illegible]	6.92E-09	5.17E-07	4.42E-07	TCIRG1/C[illegible]		39
BP	GO:00022[illegible]	lymphocy[illegible]	37/1266	194/1872[illegible]	7.98E-09	5.88E-07	5.03E-07	GPR183/H[illegible]		37
BP	GO:00019[illegible]	T cell m[illegible]	17/1266	49/18723	9.49E-09	6.90E-07	5.90E-07	HLA-A/CY[illegible]		17
BP	GO:00022[illegible]	myeloid [illegible]	40/1266	223/1872[illegible]	1.24E-08	8.91E-07	7.62E-07	LRRK2/LY[illegible]		40
BP	GO:00466[illegible]	alpha-be[illegible]	32/1266	156/1872[illegible]	1.32E-08	9.33E-07	7.97E-07	GPR183/T[illegible]		32
BP	GO:00019[illegible]	positive	13/1266	29/18723	1.42E-08	9.93E-07	8.49E-07	HLA-A/CY[illegible]		13
BP	GO:00512[illegible]	negative	32/1266	157/1872[illegible]	1.55E-08	1.07E-06	9.14E-07	LST1/LYN/		32
BP	GO:00308[illegible]	regulati[illegible]	19/1266	64/18723	2.41E-08	1.64E-06	1.40E-06	GPR183/L[illegible]		19
BP	GO:00324[illegible]	response	52/1266	343/1872[illegible]	3.43E-08	2.31E-06	1.98E-06	SERPINE1/		52
BP	GO:00160[illegible]	immunogl[illegible]	37/1266	207/1872[illegible]	4.77E-08	3.17E-06	2.71E-06	TCIRG1/H[illegible]		37
BP	GO:00456[illegible]	regulati[illegible]	33/1266	174/1872[illegible]	5.84E-08	3.82E-06	3.26E-06	PIK3R6/N[illegible]		33
BP	GO:00343[illegible]	response	29/1266	141/1872[illegible]	5.87E-08	3.82E-06	3.26E-06	GBP4/CD5[illegible]		29
BP	GO:00192[illegible]	cytokine-	64/1266	472/1872[illegible]	7.36E-08	4.72E-06	4.04E-06	CXCR6/CD[illegible]		64
BP	GO:00300[illegible]	actin fi[illegible]	27/1266	127/1872[illegible]	8.13E-08	5.16E-06	4.41E-06	PLN/WAS/[illegible]		27
BP	GO:00456[illegible]	positive	24/1266	104/1872[illegible]	8.24E-08	5.16E-06	4.41E-06	PIK3R6/I[illegible]		24
BP	GO:00716[illegible]	mononucl[illegible]	35/1266	196/1872[illegible]	1.12E-07	6.94E-06	5.93E-06	GPR183/A[illegible]		35
BP	GO:00019[illegible]	regulati[illegible]	14/1266	39/18723	1.18E-07	7.24E-06	6.19E-06	HLA-A/CY[illegible]		14
BP	GO:00072[illegible]	integrin-	24/1266	107/1872[illegible]	1.46E-07	8.86E-06	7.58E-06	ITGA1/LA[illegible]		24
BP	GO:00027[illegible]	regulati[illegible]	21/1266	85/18723	1.53E-07	9.14E-06	7.82E-06	HLA-A/WA[illegible]		21
BP	GO:00975[illegible]	granuloc[illegible]	29/1266	148/1872[illegible]	1.77E-07	1.05E-05	8.97E-06	ITGA1/RA[illegible]		29
BP	GO:00716[illegible]	granuloc[illegible]	26/1266	125/1872[illegible]	2.22E-07	1.30E-05	1.11E-05	ITGA1/RA[illegible]		26
BP	GO:00975[illegible]	calcium [illegible]	28/1266	142/1872[illegible]	2.50E-07	1.45E-05	1.24E-05	PLN/RASA[illegible]		28
BP	GO:00305[illegible]	neutroph[illegible]	23/1266	103/1872[illegible]	2.88E-07	1.65E-05	1.41E-05	ITGA1/CX[illegible]		23
BP	GO:00604[illegible]	calcium [illegible]	30/1266	160/1872[illegible]	2.99E-07	1.70E-05	1.45E-05	PLN/RASA[illegible]		30
BP	GO:00466[illegible]	positive	18/1266	67/18723	3.02E-07	1.70E-05	1.45E-05	HLA-A/FO[illegible]		18
BP	GO:00068[illegible]	superoxi[illegible]	19/1266	74/18723	3.09E-07	1.72E-05	1.47E-05	SLC1A1/N[illegible]		19
BP	GO:19037[illegible]	regulati[illegible]	52/1266	367/1872[illegible]	3.12E-07	1.72E-05	1.47E-05	CTNNB1/C[illegible]		52
BP	GO:00069[illegible]	phagocyt[illegible]	46/1266	308/1872[illegible]	3.27E-07	1.78E-05	1.53E-05	ANO6/P2R[illegible]		46
BP	GO:00508[illegible]	regulati[illegible]	34/1266	198/1872[illegible]	4.41E-07	2.38E-05	2.04E-05	GPR183/L[illegible]		34
BP	GO:00199[illegible]	second-m[illegible]	46/1266	312/1872[illegible]	4.77E-07	2.55E-05	2.18E-05	PLN/LRRK[illegible]		46
BP	GO:00455[illegible]	positive	21/1266	91/18723	5.25E-07	2.78E-05	2.38E-05	PIK3R6/F[illegible]		21
BP	GO:00027[illegible]	positive	16/1266	56/18723	5.47E-07	2.87E-05	2.45E-05	HLA-A/CY[illegible]		16
BP	GO:00604[illegible]	cytosoli[illegible]	32/1266	182/1872[illegible]	5.55E-07	2.88E-05	2.46E-05	PLN/RASA[illegible]		32
BP	GO:00027[illegible]	negative	17/1266	63/18723	5.98E-07	3.07E-05	2.63E-05	BST2/HLA-		17
BP	GO:00512[illegible]	release [illegible]	24/1266	115/1872[illegible]	6.03E-07	3.07E-05	2.63E-05	PLN/RASA[illegible]		24

BP	GO:000238	immunoglo	18/1266	70/18723	6.15E-07	3.11E-05	2.66E-05	HLA-DMA/I	18
BP	GO:005128	negative	24/1266	116/18723	7.12E-07	3.54E-05	3.02E-05	PLN/RASA3	24
BP	GO:001972	calcium-m	34/1266	202/18723	7.13E-07	3.54E-05	3.02E-05	PLN/LRRK2	34
BP	GO:000283	regulatio	47/1266	327/18723	7.58E-07	3.72E-05	3.18E-05	HLA-A/MMP	47
BP	GO:005077	negative	33/1266	194/18723	8.09E-07	3.94E-05	3.37E-05	HLA-A/MMP	33
BP	GO:000270	regulatio	30/1266	168/18723	8.86E-07	4.28E-05	3.66E-05	HLA-A/WAS	30
BP	GO:000231	mature B	11/1266	28/18723	9.51E-07	4.55E-05	3.89E-05	GPR183/DO	11
BP	GO:005128	regulatio	24/1266	118/18723	9.87E-07	4.68E-05	4.00E-05	PLN/RASA3	24
BP	GO:000242	immune re	43/1266	291/18723	1.05E-06	4.90E-05	4.19E-05	HLA-A/LYN	43
BP	GO:000275	immune re	43/1266	291/18723	1.05E-06	4.90E-05	4.19E-05	HLA-A/LYN	43
BP	GO:004663	regulatio	22/1266	104/18723	1.38E-06	6.39E-05	5.46E-05	HLA-A/FOX	22
BP	GO:000687	cellular	58/1266	448/18723	1.39E-06	6.39E-05	5.46E-05	PLN/TCIRG	58
BP	GO:000276	immune re	45/1266	315/18723	1.53E-06	6.93E-05	5.93E-05	HLA-A/LYN	45
BP	GO:005507	calcium i	59/1266	460/18723	1.54E-06	6.93E-05	5.93E-05	PLN/TCIRG	59
BP	GO:000268	regulatio	34/1266	210/18723	1.78E-06	7.94E-05	6.79E-05	ANO6/SERP	34
BP	GO:000696	cellular	15/1266	54/18723	1.83E-06	8.00E-05	6.84E-05	TCIRG1/LS	15
BP	GO:005086	negative	24/1266	122/18723	1.85E-06	8.00E-05	6.84E-05	FOXP3/LAG	24
BP	GO:005120	sequester	24/1266	122/18723	1.85E-06	8.00E-05	6.84E-05	PLN/RASA3	24
BP	GO:199026	neutrophi	24/1266	122/18723	1.85E-06	8.00E-05	6.84E-05	ITGA1/PEC	24
BP	GO:000228	T cell ac	23/1266	114/18723	1.91E-06	8.21E-05	7.02E-05	GPR183/FC	23
BP	GO:003019	collagen	16/1266	61/18723	1.93E-06	8.23E-05	7.04E-05	LOXL2/COL	16
BP	GO:000681	calcium i	55/1266	422/18723	2.12E-06	8.95E-05	7.65E-05	ANO6/PLN/	55
BP	GO:004557	regulatio	11/1266	30/18723	2.13E-06	8.95E-05	7.65E-05	NFAM1/INP	11
BP	GO:000076	syncytium	15/1266	55/18723	2.36E-06	9.76E-05	8.35E-05	CXCL9/NFA	15
BP	GO:014025	cell-cell	15/1266	55/18723	2.36E-06	9.76E-05	8.35E-05	CXCL9/NFA	15
BP	GO:003262	interleuk	16/1266	62/18723	2.44E-06	9.94E-05	8.50E-05	FOXP3/LAG	16
BP	GO:003266	regulatio	16/1266	62/18723	2.44E-06	9.94E-05	8.50E-05	FOXP3/LAG	16
BP	GO:000716	negative	43/1266	303/18723	3.10E-06	0.000125	0.000107	SERPINE1/	43
BP	GO:000270	positive	25/1266	134/18723	3.12E-06	0.000125	0.000107	HLA-A/CYP	25
BP	GO:007134	cellular	23/1266	118/18723	3.55E-06	0.000141	0.000121	GBP4/CD58	23
BP	GO:004505	T cell se	14/1266	50/18723	3.63E-06	0.000144	0.000123	FOXP3/SLA	14
BP	GO:003164	regulatio	26/1266	144/18723	3.77E-06	0.000148	0.000126	LPIN1/LRP	26
BP	GO:000694	syncytium	15/1266	57/18723	3.85E-06	0.00015	0.000128	CXCL9/NFA	15
BP	GO:004664	positive	9/1266	21/18723	4.01E-06	0.000155	0.000133	HLA-A/CD3	9
BP	GO:004255	superoxid	13/1266	44/18723	4.18E-06	0.00016	0.000137	NCF4/SYK/	13
BP	GO:005076	regulatio	20/1266	95/18723	4.45E-06	0.000169	0.000144	ANO6/IL15	20
BP	GO:003272	positive	17/1266	72/18723	4.48E-06	0.000169	0.000144	HLA-A/CYP	17
BP	GO:003362	cell adhe	17/1266	72/18723	4.48E-06	0.000169	0.000144	SERPINE1/	17
BP	GO:004663	alpha-bet	12/1266	38/18723	4.51E-06	0.000169	0.000144	HLA-A/CD3	12
BP	GO:000244	productio	43/1266	308/18723	4.75E-06	0.000176	0.000151	BST2/HLA-	43
BP	GO:004558	regulatio	26/1266	146/18723	4.90E-06	0.000181	0.000154	PIK3R6/FC	26
BP	GO:000720	positive	44/1266	319/18723	5.09E-06	0.000186	0.000159	PLN/CXCR6	44
BP	GO:004329	leukocyte	17/1266	73/18723	5.48E-06	0.000199	0.00017	LYN/HLA-E	17
BP	GO:006014	regulatio	10/1266	27/18723	5.62E-06	0.000203	0.000173	CXCL9/NFA	10
BP	GO:000234	response	12/1266	39/18723	6.12E-06	0.00022	0.000188	HLA-A/IGH	12
BP	GO:003019	extracell	42/1266	301/18723	6.19E-06	0.000221	0.000189	LOXL2/COL	42
BP	GO:000233	mature B	11/1266	33/18723	6.26E-06	0.000222	0.000189	GPR183/DO	11
BP	GO:005148	regulatio	47/1266	353/18723	6.48E-06	0.000228	0.000195	PLN/CXCR6	47
BP	GO:004306	extracell	42/1266	302/18723	6.73E-06	0.000235	0.000201	LOXL2/COL	42
BP	GO:005085	T cell re	23/1266	123/18723	7.36E-06	0.000256	0.000219	HLA-A/INP	23
BP	GO:004522	external	42/1266	304/18723	7.95E-06	0.000274	0.000235	LOXL2/COL	42
BP	GO:190303	negative	25/1266	141/18723	8.02E-06	0.000275	0.000235	FOXP3/LAG	25
BP	GO:005067	negative	18/1266	83/18723	8.55E-06	0.000292	0.000249	LST1/LYN/	18
BP	GO:004210	B cell pr	20/1266	99/18723	8.61E-06	0.000292	0.000249	GPR183/CD	20

BP	GO:00725(	cellular	59/1266	486/1872:	8.76E-06	0.000295	0.000252	PLN/TCIR(	59
BP	GO:19013	regulatio	46/1266	348/1872:	9.80E-06	0.000328	0.00028	SERPINE1/	46
BP	GO:00329	negative	18/1266	84/18723	1.02E-05	0.00034	0.00029	LST1/LYN/	18
BP	GO:00450	regulatio	33/1266	218/1872:	1.10E-05	0.000363	0.00031	HLA-A/MMI	33
BP	GO:00027(	immune re	57/1266	468/1872:	1.12E-05	0.00037	0.000316	HLA-A/LYN	57
BP	GO:00026	positive	24/1266	135/1872:	1.15E-05	0.000377	0.000322	ANO6/SERI	24
BP	GO:00466	regulatio	11/1266	35/18723	1.19E-05	0.000386	0.00033	HLA-A/CD:	11
BP	GO:00321(	positive	53/1266	427/1872:	1.33E-05	0.00043	0.000368	ANO6/LRRI	53
BP	GO:00457(	regulatio	45/1266	342/1872:	1.37E-05	0.00044	0.000376	SERPINE1/	45
BP	GO:00508	antigen r	35/1266	240/1872:	1.38E-05	0.00044	0.000376	HLA-A/LYN	35
BP	GO:00308	positive	12/1266	42/18723	1.43E-05	0.000454	0.000388	GPR183/NI	12
BP	GO:00466:	alpha-bet	21/1266	112/1872:	1.74E-05	0.000547	0.000468	GPR183/F(	21
BP	GO:00433(	positive	11/1266	37/18723	2.15E-05	0.000672	0.000574	FOXP3/SL/	11
BP	GO:00519	regulatio	36/1266	255/1872:	2.15E-05	0.000672	0.000574	PLN/CAMK2	36
BP	GO:00702	actin-mec	19/1266	97/18723	2.27E-05	0.000705	0.000603	PLN/JUP/(	19
BP	GO:00860(	cardiac n	16/1266	73/18723	2.33E-05	0.000717	0.000613	PLN/JUP/(	16
BP	GO:00312	T cell co	12/1266	44/18723	2.41E-05	0.000738	0.000631	MAP3K8/LY	12
BP	GO:00025	myeloid l	31/1266	208/1872:	2.73E-05	0.000833	0.000712	GPR183/T(	31
BP	GO:00706(	negative	18/1266	90/18723	2.76E-05	0.000837	0.000716	LST1/LYN/	18
BP	GO:00109	regulatio	50/1266	406/1872:	2.86E-05	0.000862	0.000736	ANO6/PLN/	50
BP	GO:00024	myeloid l	19/1266	99/18723	3.07E-05	0.000919	0.000785	LYN/DNASI	19
BP	GO:00359	endoderma	12/1266	45/18723	3.08E-05	0.000919	0.000785	COL5A1/CT	12
BP	GO:00022	myeloid c	18/1266	91/18723	3.23E-05	0.000958	0.000819	LYN/DNASI	18
BP	GO:00330	T cell di	16/1266	75/18723	3.32E-05	0.000974	0.000833	CTNNB1/F(	16
BP	GO:00433(	CD4-posit	17/1266	83/18723	3.32E-05	0.000974	0.000833	GPR183/F(	17
BP	GO:00712	cellular	32/1266	221/1872:	3.66E-05	0.001069	0.000914	SERPINE1/	32
BP	GO:00422(	natural k	15/1266	68/18723	3.84E-05	0.001113	0.000952	HLA-A/PII	15
BP	GO:00312	lymphocyt	12/1266	46/18723	3.92E-05	0.001124	0.000961	MAP3K8/LY	12
BP	GO:00433(	regulatio	12/1266	46/18723	3.92E-05	0.001124	0.000961	LYN/HLA-I	12
BP	GO:00069:	muscle co	44/1266	347/1872:	4.23E-05	0.001199	0.001025	PLN/CLIC2	44
BP	GO:00326:	interleuk	10/1266	33/18723	4.24E-05	0.001199	0.001025	FOXP3/HAV	10
BP	GO:00326	regulatio	10/1266	33/18723	4.24E-05	0.001199	0.001025	FOXP3/HAV	10
BP	GO:00346	response	35/1266	253/1872:	4.28E-05	0.001205	0.00103	CD58/CD7(	35
BP	GO:00026	negative	20/1266	110/1872:	4.33E-05	0.001212	0.001036	BST2/HLA-	20
BP	GO:00357	CD4-posit	19/1266	102/1872:	4.73E-05	0.00131	0.00112	GPR183/T(	19
BP	GO:00017(	endoderm	13/1266	54/18723	4.73E-05	0.00131	0.00112	COL5A1/CT	13
BP	GO:00069	humoral i	41/1266	317/1872:	4.85E-05	0.001337	0.001143	GPR183/HI	41
BP	GO:00316	regulatio	12/1266	47/18723	4.94E-05	0.001353	0.001157	LPIN1/CTN	12
BP	GO:00712	cellular	34/1266	246/1872:	5.58E-05	0.001519	0.001299	SERPINE1/	34
BP	GO:00224(	negative	29/1266	196/1872:	5.61E-05	0.001519	0.001299	FOXP3/LA(	29
BP	GO:00326(	interfero	20/1266	112/1872:	5.65E-05	0.001519	0.001299	HLA-A/CYI	20
BP	GO:00326	regulatio	20/1266	112/1872:	5.65E-05	0.001519	0.001299	HLA-A/CYI	20
BP	GO:00450(	thymic T	8/1266	22/18723	5.80E-05	0.001552	0.001327	CD3E/PTPI	8
BP	GO:00380	Fc-gamma	9/1266	28/18723	6.12E-05	0.001621	0.001386	LYN/HCK/M	9
BP	GO:00433(	negative	6/1266	12/18723	6.12E-05	0.001621	0.001386	HLA-F/CD8	6
BP	GO:00028:	positive	26/1266	168/1872:	6.22E-05	0.001634	0.001397	MMP12/LYN	26
BP	GO:00486(	positive	19/1266	104/1872:	6.23E-05	0.001634	0.001397	P2RY6/MDM	19
BP	GO:00027(	positive	20/1266	113/1872:	6.44E-05	0.001681	0.001437	HLA-A/CYI	20
BP	GO:00026	regulatio	21/1266	122/1872:	6.51E-05	0.001689	0.001444	ANO6/SERI	21
BP	GO:00022	natural k	15/1266	71/18723	6.53E-05	0.001689	0.001444	HLA-A/PII	15
BP	GO:00028	regulatio	13/1266	56/18723	7.11E-05	0.001827	0.001561	LYN/DNASI	13
BP	GO:00069	striated	27/1266	179/1872:	7.13E-05	0.001827	0.001561	PLN/CLIC2	27
BP	GO:00712	cellular	30/1266	209/1872:	7.45E-05	0.0019	0.001624	SERPINE1/	30
BP	GO:00702	lymphocyt	15/1266	72/18723	7.74E-05	0.001964	0.001679	IDO1/LYN/	15

BP	GO:00716	regulatic	20/1266	115/1872	8.31E-05	0.002087	0.001784	ANO6/SERI	20
BP	GO:00900	regulatic	9/1266	29/18723	8.34E-05	0.002087	0.001784	ANO6/SERI	9
BP	GO:00601	positive	8/1266	23/18723	8.37E-05	0.002087	0.001784	CXCL9/NF/	8
BP	GO:00030	heart pro	34/1266	251/1872	8.38E-05	0.002087	0.001784	PLN/CLIC	34
BP	GO:00600	heart cor	33/1266	241/1872	8.57E-05	0.002126	0.001817	PLN/CLIC	33
BP	GO:00024	mast cell	12/1266	50/18723	9.50E-05	0.002334	0.001995	LYN/CD84/	12
BP	GO:00466	positive	12/1266	50/18723	9.50E-05	0.002334	0.001995	FOXP3/SYI	12
BP	GO:00421	neutrophi	10/1266	36/18723	9.67E-05	0.002365	0.002022	DNASE1L3/	10
BP	GO:00023	B cell ac	16/1266	82/18723	0.000104	0.002515	0.00215	GPR183/D(	16
BP	GO:00022	neutrophi	7/1266	18/18723	0.000104	0.002515	0.00215	DNASE1L3/	7
BP	GO:00028	regulatic	27/1266	183/1872	0.000105	0.002515	0.00215	HLA-A/WAS	27
BP	GO:00509	regulatic	31/1266	223/1872	0.000105	0.002515	0.00215	GPR183/AN	31
BP	GO:00507	positive	14/1266	66/18723	0.000108	0.002594	0.002217	ANO6/IL1	14
BP	GO:00027	regulatic	25/1266	164/1872	0.00011	0.002609	0.00223	BST2/HLA-	25
BP	GO:00486	smooth mu	27/1266	184/1872	0.000115	0.002722	0.002327	CTNNB1/P	27
BP	GO:00516	maintenar	30/1266	214/1872	0.000115	0.002722	0.002327	PLN/RASA	30
BP	GO:00019	negative	8/1266	24/18723	0.000118	0.002779	0.002375	HLA-A/HL/	8
BP	GO:00030	muscle sy	52/1266	452/1872	0.000121	0.002821	0.002412	PLN/CLIC	52
BP	GO:00315	cell-subs	44/1266	363/1872	0.000122	0.002843	0.00243	ITGA1/LAN	44
BP	GO:00422	regulatic	11/1266	44/18723	0.000125	0.00289	0.002471	HLA-A/PII	11
BP	GO:00022	T cell di	15/1266	75/18723	0.000126	0.002917	0.002494	GPR183/F(	15
BP	GO:00600	cardiac m	22/1266	137/1872	0.000128	0.002952	0.002523	PLN/CLIC	22
BP	GO:00023	immunogl	30/1266	216/1872	0.000137	0.003129	0.002675	IGLV2-14/	30
BP	GO:00080	regulatic	29/1266	206/1872	0.000138	0.00315	0.002693	PLN/CLIC	29
BP	GO:00028	regulatic	25/1266	168/1872	0.000162	0.00368	0.003146	HLA-A/WAS	25
BP	GO:00327	positive	8/1266	25/18723	0.000164	0.003687	0.003151	FOXP3/HAV	8
BP	GO:00336	integrin	8/1266	25/18723	0.000164	0.003687	0.003151	RASIP1/C(	8
BP	GO:00026	positive	17/1266	94/18723	0.00017	0.003807	0.003255	ANO6/SERI	17
BP	GO:00713	cellular	31/1266	229/1872	0.000171	0.003823	0.003268	CD58/CD7(	31
BP	GO:00074	endoderm	15/1266	77/18723	0.000172	0.003829	0.003273	COL5A1/C	15
BP	GO:00027	negative	12/1266	53/18723	0.000173	0.003833	0.003277	HLA-A/HL/	12
BP	GO:00450	positive	6/1266	14/18723	0.000177	0.003905	0.003338	CD3E/PTPI	6
BP	GO:00022	alpha-bet	14/1266	69/18723	0.000179	0.003922	0.003352	GPR183/F(	14
BP	GO:00022	alpha-bet	14/1266	69/18723	0.000179	0.003922	0.003352	GPR183/F(	14
BP	GO:00450	positive	21/1266	131/1872	0.000185	0.004042	0.003455	MMP12/LYN	21
BP	GO:00488	homeostas	35/1266	272/1872	0.000186	0.004042	0.003455	GPR183/T(	35
BP	GO:00433	positive	9/1266	32/18723	0.000194	0.004207	0.003597	FOXP3/IL1	9
BP	GO:00509	positive	22/1266	141/1872	0.000197	0.004242	0.003626	ANO6/SERI	22
BP	GO:00486	regulatic	26/1266	180/1872	0.000197	0.004242	0.003626	CTNNB1/P	26
BP	GO:00512	maintenar	40/1266	327/1872	0.000201	0.004294	0.003671	PLN/RASA	40
BP	GO:00702	regulatic	12/1266	54/18723	0.000209	0.004448	0.003802	IDO1/LYN/	12
BP	GO:00025	monocyte	14/1266	70/18723	0.00021	0.004458	0.003811	ANO6/SERI	14
BP	GO:00017	leukocyte	16/1266	87/18723	0.000214	0.004507	0.003853	GPR183/T(	16
BP	GO:00022	lymphocyt	13/1266	62/18723	0.000214	0.004507	0.003853	TCIRG1/LY	13
BP	GO:00457	positive	26/1266	181/1872	0.000216	0.004517	0.003861	SERPINE1/	26
BP	GO:19040	positive	26/1266	181/1872	0.000216	0.004517	0.003861	SERPINE1/	26
BP	GO:00027	regulatic	17/1266	96/18723	0.000221	0.004599	0.003931	BST2/HLA-	17
BP	GO:00025	regulatic	7/1266	20/18723	0.000225	0.004672	0.003994	WAS/THBS1	7
BP	GO:00512	regulatic	15/1266	79/18723	0.000231	0.004782	0.004088	PLN/CLIC	15
BP	GO:00017	microglia	11/1266	47/18723	0.000235	0.004827	0.004127	LRRK2/PTI	11
BP	GO:00068	exocytosi	42/1266	352/1872	0.000242	0.00497	0.004248	RPH3AL/LI	42
BP	GO:00700	chemokine	16/1266	88/18723	0.000245	0.005002	0.004276	CXCR6/CCI	16
BP	GO:00024	Fc recept	9/1266	33/18723	0.000251	0.005116	0.004373	LYN/HCK/M	9
BP	GO:00022	T cell ac	5/1266	10/18723	0.000265	0.005377	0.004596	HAVCR2/I	5

BP	GO:00308	regulati	6/1266	15/18723	0.000278	0.005614	0.004799	INPP5D/HC	6
BP	GO:00028	positive	18/1266	107/1872	0.000284	0.005691	0.004865	HLA-A/CYP	18
BP	GO:19035	positive	18/1266	107/1872	0.000284	0.005691	0.004865	LRRK2/CD8	18
BP	GO:00023	cytokine	17/1266	98/18723	0.000285	0.005691	0.004865	BST2/HLA-	17
BP	GO:00027	regulati	11/1266	48/18723	0.000286	0.005691	0.004865	HLA-A/PIK	11
BP	GO:00024	immune re	8/1266	27/18723	0.000297	0.005855	0.005005	LYN/HCK/M	8
BP	GO:00327	negative	8/1266	27/18723	0.000297	0.005855	0.005005	FOXP3/LAG	8
BP	GO:00380	Fc-gamma	8/1266	27/18723	0.000297	0.005855	0.005005	LYN/HCK/M	8
BP	GO:00705	calcium i	38/1266	312/1872	0.000312	0.006126	0.005237	ANO6/PLN/	38
BP	GO:00336	positive	7/1266	21/18723	0.000318	0.006197	0.005298	CD3E/SYK/	7
BP	GO:00900	positive	7/1266	21/18723	0.000318	0.006197	0.005298	ANO6/SERP	7
BP	GO:00327	positive	9/1266	34/18723	0.000322	0.006197	0.005298	CD3E/PDE4	9
BP	GO:00508	negative	9/1266	34/18723	0.000322	0.006197	0.005298	LYN/INPP5	9
BP	GO:00702	regulati	9/1266	34/18723	0.000322	0.006197	0.005298	IDO1/TSC2	9
BP	GO:00716	positive	13/1266	65/18723	0.00035	0.006725	0.005749	ANO6/SERP	13
BP	GO:00725	reactive	31/1266	239/1872	0.000367	0.007032	0.006011	LRRK2/SLC	31
BP	GO:00094	response	51/1266	462/1872	0.000379	0.007225	0.006176	RPH3AL/PA	51
BP	GO:00313	negative	8/1266	28/18723	0.000392	0.007446	0.006366	HLA-A/HLA	8
BP	GO:00140	response	22/1266	148/1872	0.000396	0.007502	0.006413	SPARC/P2R	22
BP	GO:00420	T-helper	13/1266	66/18723	0.000409	0.007701	0.006583	GPR183/FO	13
BP	GO:00726	T cell mi	13/1266	66/18723	0.000409	0.007701	0.006583	GPR183/CX	13
BP	GO:00450	regulate	30/1266	230/1872	0.000412	0.007723	0.006602	LRRK2/RAP	30
BP	GO:00702	T cell ap	11/1266	50/18723	0.000417	0.007795	0.006663	IDO1/TSC2	11
BP	GO:00336	cell-cell	6/1266	16/18723	0.000419	0.007805	0.006672	CD3E/CXCL	6
BP	GO:00027	positive	12/1266	58/18723	0.000422	0.007838	0.0067	CCR1/PRKC	12
BP	GO:00027	regulati	19/1266	120/1872	0.000432	0.007995	0.006835	CTNNB1/CC	19
BP	GO:00023	defense r	5/1266	11/18723	0.000459	0.008402	0.007183	SPI1/PRF1	5
BP	GO:00323	serine tr	5/1266	11/18723	0.000459	0.008402	0.007183	SLC38A2/S	5
BP	GO:00450	negative	5/1266	11/18723	0.000459	0.008402	0.007183	CD3E/PTPR	5
BP	GO:00105	regulati	17/1266	102/1872	0.000464	0.008457	0.007229	PLN/CLIC2	17
BP	GO:00321	negative	47/1266	420/1872	0.000468	0.008504	0.007269	SERPINE1/	47
BP	GO:00362	granulocy	10/1266	43/18723	0.000473	0.008561	0.007318	DNASE1L3/	10
BP	GO:00507	regulati	44/1266	386/1872	0.000474	0.008561	0.007318	LRRK2/SER	44
BP	GO:00421	negative	13/1266	67/18723	0.000477	0.008588	0.007341	FOXP3/HAV	13
BP	GO:00017	formation	19/1266	121/1872	0.000481	0.008632	0.007379	COL5A1/CT	19
BP	GO:00903	positive	12/1266	59/18723	0.000498	0.008909	0.007616	ANO6/ARFG	12
BP	GO:00466	response	20/1266	131/1872	0.000503	0.008952	0.007652	SPARC/P2R	20
BP	GO:00028	positive	18/1266	112/1872	0.000504	0.008952	0.007652	HLA-A/CYP	18
BP	GO:00017	myeloid c	8/1266	29/18723	0.000509	0.009019	0.00771	HAVCR2/BA	8
BP	GO:00860	cardiac m	14/1266	76/18723	0.000511	0.009023	0.007713	JUP/ATP1B	14
BP	GO:00192	sensory p	17/1266	103/1872	0.000521	0.009106	0.007784	ATPSCKMT/	17
BP	GO:00327	positive	17/1266	103/1872	0.000521	0.009106	0.007784	LRRK2/CD8	17
BP	GO:00456	positive	17/1266	103/1872	0.000521	0.009106	0.007784	CCR1/PRKC	17
BP	GO:00519	positive	19/1266	122/1872	0.000534	0.009313	0.007962	CAMK2A/CC	19
BP	GO:00022	CD4-posit	13/1266	68/18723	0.000554	0.009621	0.008225	GPR183/FO	13
BP	GO:19035	regulati	32/1266	256/1872	0.000574	0.009938	0.008495	PLN/CLIC2	32
BP	GO:00313	positive	34/1266	278/1872	0.000585	0.010095	0.00863	LRRK2/SER	34
BP	GO:00525	regulati	50/1266	461/1872	0.000643	0.011063	0.009457	HIP1/PCOL	50
BP	GO:00330	muscle ce	31/1266	248/1872	0.000694	0.011902	0.010174	CTNNB1/P2	31
BP	GO:00421	macrophag	17/1266	106/1872	0.000731	0.012474	0.010663	LRRK2/CD8	17
BP	GO:00451	cellular	13/1266	70/18723	0.000739	0.012474	0.010663	ITGA1/VCA	13
BP	GO:00106	programme	5/1266	12/18723	0.000743	0.012474	0.010663	DNASE1L3/	5
BP	GO:00330	negative	5/1266	12/18723	0.000743	0.012474	0.010663	CD84/MILR	5
BP	GO:00433	negative	5/1266	12/18723	0.000743	0.012474	0.010663	CD3E/PTPR	5

BP	GO:00704	thrombin-	5/1266	12/18723	0.000743	0.012474	0.010663	F2R/PLEK/	5
BP	GO:19025	regulati	5/1266	12/18723	0.000743	0.012474	0.010663	SPI1/SYK/	5
BP	GO:19908	response	16/1266	97/18723	0.000756	0.012619	0.010787	CXCR6/CCI	16
BP	GO:19908	cellular	16/1266	97/18723	0.000756	0.012619	0.010787	CXCR6/CCI	16
BP	GO:00140	positive	14/1266	79/18723	0.000766	0.012735	0.010886	FN1/HCLS1	14
BP	GO:00717	tumor ne	25/1266	186/1872	0.000776	0.012833	0.01097	LRRK2/CD8	25
BP	GO:19035	regulati	25/1266	186/1872	0.000776	0.012833	0.01097	LRRK2/CD8	25
BP	GO:00996	cardiac m	9/1266	38/18723	0.000782	0.012892	0.011021	ATP1B1/GJ	9
BP	GO:00455	mast cell	12/1266	62/18723	0.000797	0.013093	0.011193	LYN/CD84/	12
BP	GO:00315	actin cyt	17/1266	107/1872	0.000815	0.013353	0.011415	ASB2/HCK/	17
BP	GO:00017	B cell h	8/1266	31/18723	0.000829	0.013545	0.011579	LYN/DOCK1	8
BP	GO:00860	cardiac m	11/1266	54/18723	0.000834	0.01358	0.011609	JUP/GJA5/	11
BP	GO:00726	lymphocyt	18/1266	117/1872	0.000857	0.01391	0.011891	GPR183/CX	18
BP	GO:00025	chronic i	6/1266	18/18723	0.000864	0.01391	0.011891	VCAM1/FOX	6
BP	GO:00459	negative	6/1266	18/18723	0.000864	0.01391	0.011891	HLA-A/HLA	6
BP	GO:00718	podosome	6/1266	18/18723	0.000864	0.01391	0.011891	ASAP1/ASI	6
BP	GO:00028	negative	17/1266	108/1872	0.000908	0.014526	0.012417	HLA-A/MMI	17
BP	GO:00434	positive	51/1266	480/1872	0.000908	0.014526	0.012417	GPR183/LI	51
BP	GO:00420	wound hea	46/1266	422/1872	0.000935	0.014908	0.012744	ANO6/ARF(	46
BP	GO:20005	positive	9/1266	39/18723	0.000957	0.015216	0.013007	FOXP3/IL1	9
BP	GO:00432	positive	33/1266	275/1872	0.000971	0.015398	0.013163	ANO6/CAMI	33
BP	GO:19030	positive	13/1266	72/18723	0.000974	0.015398	0.013163	ANO6/ARF(	13
BP	GO:00970	dendriti	10/1266	47/18723	0.000998	0.015733	0.01345	HLA-B/LYN	10
BP	GO:00100	response	8/1266	32/18723	0.001041	0.016365	0.013989	MDM2/SLC1	8
BP	GO:00071	cell-mat	29/1266	233/1872	0.001086	0.017027	0.014556	ITGA1/SEI	29
BP	GO:00508	regulati	42/1266	379/1872	0.001107	0.017296	0.014785	ANO6/PAM/	42
BP	GO:00508	regulati	13/1266	73/18723	0.001113	0.017345	0.014827	LRRK2/LM(	13
BP	GO:00329	transform	5/1266	13/18723	0.001141	0.017677	0.015111	NRROS/FOX	5
BP	GO:00424	gamma-del	5/1266	13/18723	0.001141	0.017677	0.015111	SYK/PTPR(	5
BP	GO:00105	positive	11/1266	56/18723	0.001146	0.017709	0.015138	P2RY6/S1I	11
BP	GO:00326	tumor ne	24/1266	181/1872	0.001185	0.017957	0.01535	LRRK2/CD8	24
BP	GO:00326	regulati	24/1266	181/1872	0.001185	0.017957	0.01535	LRRK2/CD8	24
BP	GO:00158	neutral a	10/1266	48/18723	0.001185	0.017957	0.01535	SLC1A1/SI	10
BP	GO:00336	regulati	10/1266	48/18723	0.001185	0.017957	0.01535	SERPINE1/	10
BP	GO:00433	mast cell	10/1266	48/18723	0.001185	0.017957	0.01535	LYN/CD84/	10
BP	GO:00027	negative	6/1266	19/18723	0.001192	0.017957	0.01535	HLA-A/HLA	6
BP	GO:00331	response	6/1266	19/18723	0.001192	0.017957	0.01535	CD38/MGS1	6
BP	GO:19017	regulati	6/1266	19/18723	0.001192	0.017957	0.01535	CXCL9/NFA	6
BP	GO:20011	regulati	6/1266	19/18723	0.001192	0.017957	0.01535	HLA-A/HLA	6
BP	GO:00454	bone res	12/1266	65/18723	0.001232	0.018467	0.015786	TCIRG1/CT	12
BP	GO:00706	regulati	12/1266	65/18723	0.001232	0.018467	0.015786	LRRK2/SEI	12
BP	GO:00510	regulati	47/1266	440/1872	0.001267	0.018933	0.016185	CAMK2A/F(	47
BP	GO:00363	dendriti	8/1266	33/18723	0.001294	0.019281	0.016482	GPR183/AN	8
BP	GO:00507	positive	25/1266	193/1872	0.001325	0.019698	0.016838	PECAM1/LY	25
BP	GO:00313	negative	31/1266	258/1872	0.001333	0.019754	0.016887	HLA-A/MMI	31
BP	GO:00024	dendriti	7/1266	26/18723	0.001336	0.019754	0.016887	GPR183/AN	7
BP	GO:00515	response	15/1266	93/18723	0.001381	0.020364	0.017408	SPARC/AQI	15
BP	GO:00022	mast cell	10/1266	49/18723	0.0014	0.020592	0.017603	LYN/CD84/	10
BP	GO:00313	positive	29/1266	237/1872	0.001417	0.020777	0.017761	RASIP1/FN	29
BP	GO:00336	positive	49/1266	467/1872	0.001478	0.021618	0.01848	LRRK2/SL(	49
BP	GO:00096	response	27/1266	216/1872	0.001486	0.021681	0.018534	CTNNB1/JU	27
BP	GO:00068	amino aci	20/1266	143/1872	0.001538	0.022374	0.019126	SLC38A8/S	20
BP	GO:00355	purinergi	8/1266	34/18723	0.001593	0.023108	0.019754	ANO6/GPR3	8
BP	GO:00466	gamma-del	6/1266	20/18723	0.001606	0.023235	0.019862	JAML/SYK/	6

BP	GO:00105]	regulatic	12/1266	67/18723	0.00162	0.023314	0.01993	P2RY6/S1I	12
BP	GO:19033]	regulatic	12/1266	67/18723	0.00162	0.023314	0.01993	LRRK2/SEI	12
BP	GO:00380(	Fc recept	10/1266	50/18723	0.001646	0.023633	0.020202	LYN/HCK/N	10
BP	GO:00487	tissue re	23/1266	175/1872	0.00167	0.023634	0.020203	TCIRG1/CT	23
BP	GO:00017	immunolog	5/1266	14/18723	0.001676	0.023634	0.020203	HAVCR2/PI	5
BP	GO:00024	antigen p	5/1266	14/18723	0.001676	0.023634	0.020203	HLA-F/HLA	5
BP	GO:00455	positive	5/1266	14/18723	0.001676	0.023634	0.020203	INPP5D/SY	5
BP	GO:00075	myoblast	9/1266	42/18723	0.001677	0.023634	0.020203	CXCL9/NFA	9
BP	GO:00305	collagen	9/1266	42/18723	0.001677	0.023634	0.020203	MMP12/FAI	9
BP	GO:00330(	regulatic	9/1266	42/18723	0.001677	0.023634	0.020203	LYN/CD84/	9
BP	GO:00360	CD8-posit	7/1266	27/18723	0.0017	0.023838	0.020377	HLA-A/HLA	7
BP	GO:00996	ventricul	7/1266	27/18723	0.0017	0.023838	0.020377	GJA5/KCNI	7
BP	GO:00431	positive	24/1266	186/1872	0.001726	0.024072	0.020578	BST2/CTNN	24
BP	GO:00326	interleuk	22/1266	165/1872	0.00173	0.024072	0.020578	HLA-B/INI	22
BP	GO:00326	regulatic	22/1266	165/1872	0.00173	0.024072	0.020578	HLA-B/INI	22
BP	GO:00030]	vascular	31/1266	263/1872	0.001812	0.025147	0.021497	ITGA1/SLC	31
BP	GO:00216	nerve dev	13/1266	77/18723	0.001845	0.025524	0.021819	TCIRG1/GA	13
BP	GO:00466	regulatic	12/1266	68/18723	0.001848	0.025524	0.021819	FOXP3/SYI	12
BP	GO:00433	regulatic	10/1266	51/18723	0.001926	0.026529	0.022678	FOXP3/IL1	10
BP	GO:00973(	response	30/1266	253/1872	0.00194	0.026657	0.022788	CTNNB1/VC	30
BP	GO:00096]	response	40/1266	367/1872	0.001949	0.026716	0.022838	BST2/EXOS	40
BP	GO:00020(	chondrocy	16/1266	106/1872	0.001988	0.027107	0.023173	LOXL2/CTN	16
BP	GO:00718	leukocyte	16/1266	106/1872	0.001988	0.027107	0.023173	IDO1/LYN/	16
BP	GO:00420	T-helper	9/1266	43/18723	0.001995	0.027144	0.023204	IL18BP/HA	9
BP	GO:19030	regulatic	22/1266	167/1872	0.00202	0.027406	0.023428	ANO6/ARFC	22
BP	GO:00516(	defense r	31/1266	265/1872	0.002041	0.027561	0.02356	BST2/EXOS	31
BP	GO:01405	defense r	31/1266	265/1872	0.002041	0.027561	0.02356	BST2/EXOS	31
BP	GO:00316(	lipopolys	11/1266	60/18723	0.002059	0.027735	0.023709	MTDH/LYN/	11
BP	GO:00601	positive	12/1266	69/18723	0.002102	0.028248	0.024147	P2RY6/S1I	12
BP	GO:00995]	actin fil	6/1266	21/18723	0.002121	0.028425	0.024299	MYO1G/MYC	6
BP	GO:00025(	tolerance	7/1266	28/18723	0.002136	0.028488	0.024352	HLA-B/LYN	7
BP	GO:00455	regulatic	7/1266	28/18723	0.002136	0.028488	0.024352	FOXP3/LAC	7
BP	GO:00432	regulatic	45/1266	428/1872	0.002168	0.028841	0.024654	RASIP1/AI	45
BP	GO:00300	myeloid c	41/1266	381/1872	0.002184	0.028991	0.024783	GPR183/TC	41
BP	GO:00102	vascular	14/1266	88/18723	0.002247	0.02975	0.025431	SLC1A1/SI	14
BP	GO:00420	type 2 in	8/1266	36/18723	0.002354	0.03082	0.026347	BATF/IL18	8
BP	GO:00033	amino aci	15/1266	98/18723	0.002357	0.03082	0.026347	SLC38A8/S	15
BP	GO:00451	regulatic	9/1266	44/18723	0.002361	0.03082	0.026347	PRKCA/INI	9
BP	GO:01500	neuroinfl	9/1266	44/18723	0.002361	0.03082	0.026347	LRRK2/ADC	9
BP	GO:00341]	heterotyp	11/1266	61/18723	0.002362	0.03082	0.026347	CD58/VCAN	11
BP	GO:00430	regulatic	11/1266	61/18723	0.002362	0.03082	0.026347	LRRK2/CD8	11
BP	GO:00519	regulatic	46/1266	443/1872	0.002489	0.032399	0.027696	LPIN1/MAI	46
BP	GO:00158	organic a	34/1266	303/1872	0.00256	0.033244	0.028419	SLC38A8/S	34
BP	GO:00109	negative	7/1266	29/18723	0.002653	0.034301	0.029321	LRRK2/SEI	7
BP	GO:19033]	negative	7/1266	29/18723	0.002653	0.034301	0.029321	LRRK2/SEI	7
BP	GO:00068	chloride	16/1266	109/1872	0.00266	0.034302	0.029323	ANO6/GABI	16
BP	GO:00510	negative	22/1266	171/1872	0.002724	0.035049	0.029961	CYP51A1/V	22
BP	GO:00023(	T cell cy	8/1266	37/18723	0.002828	0.035891	0.030681	HLA-A/HLA	8
BP	GO:00027	regulatic	8/1266	37/18723	0.002828	0.035891	0.030681	HLA-A/HLA	8
BP	GO:00316	positive	8/1266	37/18723	0.002828	0.035891	0.030681	ATPSCKMT/	8
BP	GO:00430	T cell hc	8/1266	37/18723	0.002828	0.035891	0.030681	TCIRG1/FC	8
BP	GO:00457	respirato	8/1266	37/18723	0.002828	0.035891	0.030681	HCK/NCF4/	8
BP	GO:00075(	female pr	24/1266	193/1872	0.002829	0.035891	0.030681	IDO1/PAM/	24
BP	GO:00030]	renal sys	16/1266	110/1872	0.002922	0.036902	0.031545	RRM2B/WNI	16

BP	GO:00487	appendage	22/1266	172/18723	0.00293	0.036902	0.031545	PAM/KREMI	22
BP	GO:00601	limb deve	22/1266	172/18723	0.00293	0.036902	0.031545	PAM/KREMI	22
BP	GO:20001	regulatic	13/1266	81/18723	0.002936	0.036902	0.031545	IDO1/LYN/	13
BP	GO:00508	regulatic	11/1266	63/18723	0.003075	0.038555	0.032959	LYN/NFAM1	11
BP	GO:00028	negative	4/1266	10/18723	0.00314	0.039197	0.033507	CD84/SPI1	4
BP	GO:20004	regulatic	4/1266	10/18723	0.00314	0.039197	0.033507	C4A/CD30	4
BP	GO:00508	defense r	15/1266	101/18723	0.003177	0.039475	0.033745	GBP4/HLA-	15
BP	GO:00988	cellular	15/1266	101/18723	0.003177	0.039475	0.033745	APOM/CLIC	15
BP	GO:00433	regulatic	7/1266	30/18723	0.003262	0.040378	0.034517	LYN/CD84/	7
BP	GO:00019	renal sys	5/1266	16/18723	0.003264	0.040378	0.034517	GJA5/F2R/	5
BP	GO:00182	peptidyl-	40/1266	378/18723	0.003305	0.040792	0.03487	MATK/PECA	40
BP	GO:00508	synapse c	44/1266	426/18723	0.003365	0.041434	0.03542	LRRK2/CTN	44
BP	GO:00519	regulatic	8/1266	38/18723	0.003373	0.041446	0.03543	ATPSCKMT/	8
BP	GO:00456	negative	6/1266	23/18723	0.003512	0.04286	0.036638	NMRK2/SOS	6
BP	GO:00726	eosinophi	6/1266	23/18723	0.003512	0.04286	0.036638	CCL4/PTGI	6
BP	GO:19033	negative	6/1266	23/18723	0.003512	0.04286	0.036638	HLA-F/CD8	6
BP	GO:00082	regulatic	23/1266	186/18723	0.003671	0.044702	0.038213	RARRES2/I	23
BP	GO:00507	regulatic	30/1266	264/18723	0.003681	0.044718	0.038226	PECAM1/LY	30
BP	GO:00149	positive	9/1266	47/18723	0.003782	0.045704	0.03907	P2RY6/MDM	9
BP	GO:00484	autonomic	9/1266	47/18723	0.003782	0.045704	0.03907	CTNNB1/VC	9
BP	GO:00973	cellular	14/1266	93/18723	0.003787	0.045704	0.03907	CTNNB1/P2	14
BP	GO:00140	phosphati	19/1266	144/18723	0.003873	0.046644	0.039873	FN1/HCLS1	19
BP	GO:00447	multi-mul	26/1266	220/18723	0.00391	0.04698	0.04016	IDO1/PAM/	26
BP	GO:00027	positive	11/1266	65/18723	0.003951	0.047037	0.040209	HLA-A/HLA	11
BP	GO:00020	chondrocy	7/1266	31/18723	0.003972	0.047037	0.040209	SULF2/SOX	7
BP	GO:00330	regulatic	7/1266	31/18723	0.003972	0.047037	0.040209	LYN/CD84/	7
BP	GO:00450	regulator	7/1266	31/18723	0.003972	0.047037	0.040209	FOXP3/LAG	7
BP	GO:00070	actin fil	45/1266	442/18723	0.003985	0.047037	0.040209	HIP1/ARFC	45
BP	GO:00424	dopamine	8/1266	39/18723	0.003997	0.047037	0.040209	SLC1A1/CC	8
BP	GO:00519	regulatic	8/1266	39/18723	0.003997	0.047037	0.040209	ATPSCKMT/	8
BP	GO:20002	regulatic	8/1266	39/18723	0.003997	0.047037	0.040209	HCK/HCLS1	8
BP	GO:00015	action pc	18/1266	134/18723	0.00401	0.047037	0.040209	JUP/ATP1I	18
BP	GO:00425	myelinati	18/1266	134/18723	0.00401	0.047037	0.040209	LPIN1/CTN	18
BP	GO:00610	regulatic	18/1266	134/18723	0.00401	0.047037	0.040209	ANO6/ARFC	18
BP	GO:00347	regulatic	49/1266	491/18723	0.00406	0.047525	0.040626	ANO6/PLN/	49
BP	GO:00456	regulatic	25/1266	210/18723	0.004188	0.048876	0.041781	CTNNB1/CC	25
BP	GO:00197	polyol me	16/1266	114/18723	0.004193	0.048876	0.041781	INPP4B/P2	16
BP	GO:00329	collagen	15/1266	104/18723	0.004218	0.049064	0.041942	COL5A1/MM	15
BP	GO:00525	regulatic	44/1266	432/18723	0.004344	0.05029	0.04299	HIP1/SERI	44
BP	GO:00162	gap junct	5/1266	17/18723	0.00437	0.05029	0.04299	APLNR/GJA	5
BP	GO:00455	positive	5/1266	17/18723	0.00437	0.05029	0.04299	FOXP3/LII	5
BP	GO:00901	epithelia	5/1266	17/18723	0.00437	0.05029	0.04299	JUP/CAMSA	5
BP	GO:20006	regulatic	5/1266	17/18723	0.00437	0.05029	0.04299	CTNNB1/WW	5
BP	GO:00481	positive	9/1266	48/18723	0.004381	0.050317	0.043013	FN1/AQP1/	9
BP	GO:00075	hemostasi	26/1266	222/18723	0.004412	0.050559	0.04322	ANO6/SERI	26
BP	GO:00480	phosphati	22/1266	178/18723	0.004455	0.050946	0.04355	PLCD1/CA8	22
BP	GO:00018	tissue hc	30/1266	268/18723	0.004579	0.052259	0.044673	TCIRG1/TI	30
BP	GO:19026	proton tr	20/1266	157/18723	0.004635	0.052284	0.044695	TCIRG1/AT	20
BP	GO:00030	regulatic	4/1266	11/18723	0.004672	0.052284	0.044695	GJA5/F2R/	4
BP	GO:00060	fructose	4/1266	11/18723	0.004672	0.052284	0.044695	FBP2/HK3/	4
BP	GO:00336	regulatic	4/1266	11/18723	0.004672	0.052284	0.044695	CD3E/CXCI	4
BP	GO:00421	neurotrar	4/1266	11/18723	0.004672	0.052284	0.044695	BCHE/COMT	4
BP	GO:00433	neutrophi	4/1266	11/18723	0.004672	0.052284	0.044695	SPI1/SYK/	4
BP	GO:00466	regulatic	4/1266	11/18723	0.004672	0.052284	0.044695	SYK/PTPRC	4

BP	GO:00724	mesenchym	4/1266	11/18723	0.004672	0.052284	0.044695	CTNNB1/S(	4
BP	GO:00072	ensheathm	18/1266	136/1872	0.004697	0.052284	0.044695	LPIN1/CTN	18
BP	GO:00083(	axon ensh	18/1266	136/1872	0.004697	0.052284	0.044695	LPIN1/CTN	18
BP	GO:00069	response	45/1266	446/1872	0.004699	0.052284	0.044695	LRRK2/CTN	45
BP	GO:00108(	positive	8/1266	40/18723	0.004706	0.052284	0.044695	P2RY6/S1F	8
BP	GO:01500	regulatio	8/1266	40/18723	0.004706	0.052284	0.044695	LRRK2/PTF	8
BP	GO:00357(	endotheli	7/1266	32/18723	0.004792	0.053134	0.045421	SMOC2/P2F	7
BP	GO:19035	negative	19/1266	147/1872	0.004862	0.053803	0.045993	CYP51A1/F	19
BP	GO:00181(	peptidyl-	39/1266	375/1872	0.004943	0.054579	0.046656	MATK/PECA	39
BP	GO:19907	cellular	16/1266	116/1872	0.004981	0.05489	0.046922	APOM/CLIC	16
BP	GO:20005	regulatio	11/1266	67/18723	0.005015	0.055159	0.047152	FOXP3/IL1	11
BP	GO:00302	macrophag	9/1266	49/18723	0.005052	0.05534	0.047307	PRKCA/NRF	9
BP	GO:00860(	membrane	9/1266	49/18723	0.005052	0.05534	0.047307	ATP1B1/GJ	9
BP	GO:00970	regulatio	10/1266	58/18723	0.005124	0.05602	0.047888	ANO6/ABCF	10
BP	GO:00069	phagocyto	17/1266	127/1872	0.005264	0.057426	0.04909	ANO6/MEGF	17
BP	GO:19031(	regulatio	20/1266	159/1872	0.005344	0.058189	0.049742	PLN/CLIC2	20
BP	GO:00027(	positive	16/1266	117/1872	0.005418	0.058754	0.050225	HLA-A/HLA	16
BP	GO:00149(	myotube (	16/1266	117/1872	0.005418	0.058754	0.050225	TMEM119/(	16
BP	GO:00030	glomerula	6/1266	25/18723	0.005487	0.05914	0.050556	SULF2/GJA	6
BP	GO:00985	detection	6/1266	25/18723	0.005487	0.05914	0.050556	HLA-A/HLA	6
BP	GO:01501(	transport	13/1266	87/18723	0.005507	0.05914	0.050556	SLC1A1/SI	13
BP	GO:00508	regulatio	8/1266	41/18723	0.005507	0.05914	0.050556	SLA2/GBP1	8
BP	GO:20000(	regulatio	8/1266	41/18723	0.005507	0.05914	0.050556	CTNNB1/CI	8
BP	GO:00703	ERK1 and	35/1266	330/1872	0.005534	0.059311	0.050701	GPR183/C(	35
BP	GO:19050	carboxyli	19/1266	149/1872	0.005631	0.060226	0.051483	SLC38A8/S	19
BP	GO:00026	regulatio	5/1266	18/18723	0.005718	0.060803	0.051977	HLA-B/FO	5
BP	GO:00308	negative	5/1266	18/18723	0.005718	0.060803	0.051977	LYN/INPP5	5
BP	GO:00316	positive	5/1266	18/18723	0.005718	0.060803	0.051977	EGR2/ITGA	5
BP	GO:00459	negative	7/1266	33/18723	0.005734	0.060857	0.052023	HLA-F/CD8	7
BP	GO:00480	inositol	22/1266	182/1872	0.0058	0.061437	0.052519	PLCD1/CA8	22
BP	GO:00093(	amine met	16/1266	118/1872	0.005885	0.062214	0.053183	IDO1/VCAM	16
BP	GO:00986	import ir	26/1266	227/1872	0.005907	0.062323	0.053276	SLC1A1/WN	26
BP	GO:00714	cellular	34/1266	320/1872	0.006023	0.063429	0.054222	LRRK2/DAF	34
BP	GO:19038	organic a	19/1266	150/1872	0.00605	0.063593	0.054362	SLC38A8/S	19
BP	GO:00603	bone deve	24/1266	205/1872	0.006093	0.063915	0.054637	SULF2/TMF	24
BP	GO:00512	protein p	32/1266	297/1872	0.006171	0.064604	0.055226	ARFGEF1/F	32
BP	GO:00072	Notch sig	21/1266	172/1872	0.006182	0.064604	0.055226	PLN/KCNA5	21
BP	GO:00703	regulatio	33/1266	309/1872	0.00625	0.065191	0.055728	GPR183/C(	33
BP	GO:00075	blood coa	25/1266	217/1872	0.006369	0.066303	0.056678	ANO6/SERF	25
BP	GO:19002	regulatio	8/1266	42/18723	0.006409	0.066591	0.056925	P2RY6/S1F	8
BP	GO:19040(	positive	19/1266	151/1872	0.006495	0.067362	0.057584	ANO6/ATPS	19
BP	GO:00468	regulatio	9/1266	51/18723	0.00663	0.068105	0.058219	PRKCA/INF	9
BP	GO:00024	T cell me	4/1266	12/18723	0.006637	0.068105	0.058219	HLA-A/MR1	4
BP	GO:00026(	regulatio	4/1266	12/18723	0.006637	0.068105	0.058219	HLA-B/FO	4
BP	GO:00148	gastro-in	4/1266	12/18723	0.006637	0.068105	0.058219	SULF2/PTA	4
BP	GO:00329(	regulatio	4/1266	12/18723	0.006637	0.068105	0.058219	FOXP3/THF	4
BP	GO:00614	connecti	28/1266	252/1872	0.006647	0.068105	0.058219	LOXL2/COI	28
BP	GO:00424	odontoger	17/1266	130/1872	0.006654	0.068105	0.058219	PAM/TCIR(	17
BP	GO:00332(	tumor nec	14/1266	99/18723	0.006668	0.068116	0.058228	CD70/SYK/	14
BP	GO:00024	immune re	6/1266	26/18723	0.006732	0.068259	0.058351	HLA-A/HAV	6
BP	GO:00027	negative	6/1266	26/18723	0.006732	0.068259	0.058351	HLA-F/FO	6
BP	GO:00459	positive	6/1266	26/18723	0.006732	0.068259	0.058351	HLA-F/LAC	6
BP	GO:00972(	renal fil	6/1266	26/18723	0.006732	0.068259	0.058351	SULF2/GJA	6
BP	GO:00308	granulocy	7/1266	34/18723	0.006808	0.068773	0.05879	INPP5D/H(	7

BP	GO:00860(	regulatio	7/1266	34/18723	0.006808	0.068773	0.05879	PLN/JUP/(	7
BP	GO:00508	positive	19/1266	152/1872	0.006967	0.070247	0.06005	GPR183/IN	19
BP	GO:00075	response	21/1266	174/1872	0.007043	0.07089	0.0606	VCAM1/SPA	21
BP	GO:00148	muscle ce	15/1266	110/1872	0.007134	0.071481	0.061105	SERPINE1/	15
BP	GO:00189	phenol-co	15/1266	110/1872	0.007134	0.071481	0.061105	SLC1A1/AS	15
BP	GO:00347(	positive	25/1266	219/1872	0.007142	0.071481	0.061105	ANO6/ATPS	25
BP	GO:00481	regulatio	12/1266	80/18723	0.007268	0.071892	0.061456	CTNNB1/FN	12
BP	GO:00430	regulatio	36/1266	348/1872	0.007305	0.071892	0.061456	LRRK2/RAS	36
BP	GO:00109	regulatio	44/1266	445/1872	0.007323	0.071892	0.061456	LRRK2/MAI	44
BP	GO:00028	positive	5/1266	19/18723	0.007335	0.071892	0.061456	SPI1/HLA-	5
BP	GO:00300	vesicle t	5/1266	19/18723	0.007335	0.071892	0.061456	MYO1G/MY(	5
BP	GO:00456	positive	5/1266	19/18723	0.007335	0.071892	0.061456	MACROH2A2	5
BP	GO:00457	positive	5/1266	19/18723	0.007335	0.071892	0.061456	PRKCA/SYK	5
BP	GO:00702	negative	5/1266	19/18723	0.007335	0.071892	0.061456	TSC22D3/S	5
BP	GO:00720	renal ves	5/1266	19/18723	0.007335	0.071892	0.061456	CTNNB1/FN	5
BP	GO:19039	regulatio	5/1266	19/18723	0.007335	0.071892	0.061456	LRRK2/PTI	5
BP	GO:00341(	homotypic	13/1266	90/18723	0.007341	0.071892	0.061456	JUP/LYN/I	13
BP	GO:00468	bone remo	13/1266	90/18723	0.007341	0.071892	0.061456	TCIRG1/CT	13
BP	GO:00716(	transform	8/1266	43/18723	0.007418	0.072515	0.061989	NRROS/FOX	8
BP	GO:00022	activatio	9/1266	52/18723	0.007548	0.073266	0.062631	LYN/STIN(	9
BP	GO:00215	cranial r	9/1266	52/18723	0.007548	0.073266	0.062631	TCIRG1/CT	9
BP	GO:00342(	lipid tra	9/1266	52/18723	0.007548	0.073266	0.062631	ANO6/ABCE	9
BP	GO:00432	apoptotic	9/1266	52/18723	0.007548	0.073266	0.062631	MEGF10/C4	9
BP	GO:00140(	regulatio	15/1266	111/1872	0.007748	0.075075	0.064177	FN1/HCLS1	15
BP	GO:00327	positive	11/1266	71/18723	0.007817	0.075476	0.06452	CD84/HAV(	11
BP	GO:00458	negative	11/1266	71/18723	0.007817	0.075476	0.06452	HLA-A/MMI	11
BP	GO:00720(	renal sys	32/1266	302/1872	0.007843	0.075593	0.06462	LRRK2/LAN	32
BP	GO:00065	cellular	14/1266	101/1872	0.007946	0.076443	0.065346	IDO1/SLC1	14
BP	GO:00481	fibroblas	12/1266	81/18723	0.008022	0.076627	0.065504	CTNNB1/FN	12
BP	GO:00712(	cellular	12/1266	81/18723	0.008022	0.076627	0.065504	PIEZO2/HI	12
BP	GO:00328	regulatio	7/1266	35/18723	0.008024	0.076627	0.065504	HLA-F/IL1	7
BP	GO:00988(	regulatio	7/1266	35/18723	0.008024	0.076627	0.065504	GJA5/EDNI	7
BP	GO:00199	cyclic-nu	13/1266	91/18723	0.008049	0.076627	0.065504	PDE3A/API	13
BP	GO:00601	regulatio	13/1266	91/18723	0.008049	0.076627	0.065504	P2RY6/S1I	13
BP	GO:00197	antimicro	16/1266	122/1872	0.008091	0.076891	0.065729	HLA-A/RAI	16
BP	GO:00456(	positive	6/1266	27/18723	0.00817	0.077507	0.066256	KDF1/MACI	6
BP	GO:00322	regulatio	26/1266	233/1872	0.008233	0.077969	0.066651	ARFGEF1/I	26
BP	GO:00508	coagulati	25/1266	222/1872	0.008444	0.079828	0.06824	ANO6/SERI	25
BP	GO:00083	glial cel	9/1266	53/18723	0.00856	0.08079	0.069063	GPR183/CT	9
BP	GO:00506	epithelia	43/1266	437/1872	0.008627	0.081245	0.069451	LOXL2/CTN	43
BP	GO:00326	regulatio	14/1266	102/1872	0.008653	0.081245	0.069451	CD58/SERI	14
BP	GO:19021(	negative	14/1266	102/1872	0.008653	0.081245	0.069451	CTNNB1/LY	14
BP	GO:00082	regulatio	17/1266	134/1872	0.008956	0.083943	0.071758	CAMK2A/PI	17
BP	GO:19020	positive	4/1266	13/18723	0.009079	0.084952	0.07262	THBS1/BMI	4
BP	GO:00441(	cellular	15/1266	113/1872	0.009102	0.085023	0.072681	IDO1/SLC1	15
BP	GO:00347(	positive	20/1266	167/1872	0.009125	0.085093	0.072741	ANO6/ATPS	20
BP	GO:00434	response	41/1266	414/1872	0.009152	0.085103	0.07275	PLN/LPIN1	41
BP	GO:20001	regulatio	26/1266	235/1872	0.009158	0.085103	0.07275	HIP1/SLC1	26
BP	GO:00031	morphogen	5/1266	20/18723	0.009244	0.085326	0.07294	CTNNB1/IT	5
BP	GO:00430	myeloid c	5/1266	20/18723	0.009244	0.085326	0.07294	BATF/SPI1	5
BP	GO:00611	endotheli	5/1266	20/18723	0.009244	0.085326	0.07294	CTNNB1/IT	5
BP	GO:00720	renal ves	5/1266	20/18723	0.009244	0.085326	0.07294	CTNNB1/FN	5
BP	GO:00020	regulatio	14/1266	103/1872	0.00941	0.086491	0.073936	PLN/SLC1A	14
BP	GO:00326	interleuk	14/1266	103/1872	0.00941	0.086491	0.073936	CD58/SERI	14

BP	GO:00972:	cellular	16/1266	124/1872:	0.009418	0.086491	0.073936	APOM/CLI(		16
BP	GO:00026:	regulatic	11/1266	73/18723	0.009612	0.087645	0.074922	FOXP3/CG/		11
BP	GO:00327:	positive	13/1266	93/18723	0.009626	0.087645	0.074922	SYK/F2R/I		13
BP	GO:00028:	negative	9/1266	54/18723	0.009672	0.087645	0.074922	HLA-F/FO)		9
BP	GO:00065(	catechol:	9/1266	54/18723	0.009672	0.087645	0.074922	SLC1A1/SI		9
BP	GO:00097]	catechol-	9/1266	54/18723	0.009672	0.087645	0.074922	SLC1A1/SI		9
BP	GO:00105:	positive	9/1266	54/18723	0.009672	0.087645	0.074922	P2RY6/CX(		9
BP	GO:00436	inositol	9/1266	54/18723	0.009672	0.087645	0.074922	INPP4B/P:		9
BP	GO:00603:	endochon(	9/1266	54/18723	0.009672	0.087645	0.074922	TMEM119/:		9
BP	GO:00108]	T cell cl	6/1266	28/18723	0.009815	0.088501	0.075654	GPR183/C)		6
BP	GO:00860]	membrane	6/1266	28/18723	0.009815	0.088501	0.075654	ATP1B1/K(		6
BP	GO:00996:	regulatic	6/1266	28/18723	0.009815	0.088501	0.075654	GJA5/KCNI		6
BP	GO:00080:	cell rec(	25/1266	225/1872:	0.009934	0.089422	0.076442	PECAM1/CI		25
BP	GO:00300	actin fi]	22/1266	191/1872:	0.010072	0.090513	0.077374	ARFGEF1/I		22
BP	GO:00720	kidney e]	17/1266	136/1872:	0.010324	0.092033	0.078673	LAMA5/IR)		17
BP	GO:00990:	plasma me	17/1266	136/1872:	0.010324	0.092033	0.078673	ANO6/MEGI		17
BP	GO:19033(	regulatic	17/1266	136/1872:	0.010324	0.092033	0.078673	LRRK2/RAI		17
BP	GO:00468:	phosphati	10/1266	64/18723	0.010325	0.092033	0.078673	INPP4B/PI		10
BP	GO:00482	lymphocy	10/1266	64/18723	0.010325	0.092033	0.078673	GPR183/C)		10
BP	GO:00303]	osteocla:	13/1266	94/18723	0.0105	0.09344	0.079876	GPR183/T(		13
BP	GO:00987	cell-cel]	29/1266	273/1872:	0.010626	0.094407	0.080703	VCAM1/L1(		29
BP	GO:00454	myoblast	12/1266	84/18723	0.010653	0.094493	0.080776	NMRK2/CX(		12
BP	GO:19040(	regulatic	36/1266	357/1872:	0.010716	0.094898	0.081123	ANO6/PLN/		36
BP	GO:00715(	cellular	27/1266	250/1872:	0.010887	0.096253	0.082281	SOX5/PDE:		27
BP	GO:00069	regulatic	13/1266	95/18723	0.011435	0.100921	0.086271	PLN/CLIC2		13
BP	GO:00337(	response	5/1266	21/18723	0.01147	0.100921	0.086271	CYC1/QDPI		5
BP	GO:00433	CD4-posi	5/1266	21/18723	0.01147	0.100921	0.086271	FOXP3/SL/		5
BP	GO:00343:	cell jun(	41/1266	420/1872:	0.011507	0.101084	0.086411	CTNNB1/LI		41
BP	GO:00510:	negative	45/1266	470/1872:	0.01167	0.102139	0.087313	PLN/LRRK2		45
BP	GO:00023(	T cell li	6/1266	29/18723	0.011684	0.102139	0.087313	FOXP3/SL/		6
BP	GO:00343]	diol met:	6/1266	29/18723	0.011684	0.102139	0.087313	QDPR/SPHI		6
BP	GO:00426	muscle c	38/1266	384/1872:	0.011903	0.103434	0.088419	CTNNB1/FI		38
BP	GO:19037(	negative	14/1266	106/1872:	0.011995	0.103434	0.088419	CTNNB1/L)		14
BP	GO:00025]	T cell t	4/1266	14/18723	0.01204	0.103434	0.088419	HLA-B/FO)		4
BP	GO:00031(	heart va]	4/1266	14/18723	0.01204	0.103434	0.088419	GJA5/SOX!		4
BP	GO:00108:	negative	4/1266	14/18723	0.01204	0.103434	0.088419	KDF1/CD1(		4
BP	GO:00311]	negative	4/1266	14/18723	0.01204	0.103434	0.088419	CLIP3/MAI		4
BP	GO:00355(	G protei	4/1266	14/18723	0.01204	0.103434	0.088419	GPR34/P2I		4
BP	GO:00433	CD8-posi	4/1266	14/18723	0.01204	0.103434	0.088419	IRF1/NCK/		4
BP	GO:00454	response	4/1266	14/18723	0.01204	0.103434	0.088419	MDM2/FOXI		4
BP	GO:00721(	nephron	4/1266	14/18723	0.01204	0.103434	0.088419	CTNNB1/W\		4
BP	GO:00907]	immunolo	4/1266	14/18723	0.01204	0.103434	0.088419	ST3GAL1/]		4
BP	GO:00464:	icosanoi(	9/1266	56/18723	0.012218	0.104796	0.089584	MGST2/SYI		9
BP	GO:00488(	stem cel]	23/1266	206/1872:	0.01233	0.105593	0.090265	LAMA5/CT		23
BP	GO:00100:	response	37/1266	373/1872:	0.012497	0.106852	0.091342	PAM/PLN/		37
BP	GO:00703(	positive	16/1266	128/1872:	0.012588	0.107462	0.091863	GADD45G/I		16
BP	GO:00095!	detectio	7/1266	38/18723	0.012629	0.107478	0.091877	HLA-A/HL/		7
BP	GO:00482	macropha	7/1266	38/18723	0.012629	0.107478	0.091877	RARRES2/I		7
BP	GO:00453:	phosphol	8/1266	47/18723	0.012679	0.107562	0.091949	ANO6/ABCI		8
BP	GO:00456	regulatic	8/1266	47/18723	0.012679	0.107562	0.091949	LYN/INPP:		8
BP	GO:00488(	stem cel]	12/1266	86/18723	0.012747	0.107976	0.092302	LAMA5/RAI		12
BP	GO:00458(	positive	38/1266	386/1872:	0.012863	0.108785	0.092994	LRRK2/SL(		38
BP	GO:00016:	urogenit:	34/1266	338/1872:	0.013312	0.112414	0.096096	LRRK2/LA\		34
BP	GO:00149(	smooth m	13/1266	97/18723	0.013496	0.11379	0.097273	SERPINE1/		13

BP	GO:00163[illegible]	dendrite	26/1266	243/1872[illegible]	0.013742	0.115545	0.098773	LRRK2/MA[illegible]	26
BP	GO:001967	NAD metal	6/1266	30/18723	0.013789	0.115545	0.098773	NMRK2/HK[illegible]	6
BP	GO:00508[illegible]	negative	6/1266	30/18723	0.013789	0.115545	0.098773	SLA2/GBP1	6
BP	GO:00702[illegible]	negative	6/1266	30/18723	0.013789	0.115545	0.098773	TSC22D3/[illegible]	6
BP	GO:000751	muscle o	33/1266	327/1872[illegible]	0.013951	0.11653	0.099615	SAP30/LA[illegible]	33
BP	GO:00020[illegible]	positive	5/1266	22/18723	0.014035	0.11653	0.099615	CTNNB1/P[illegible]	5
BP	GO:00023[illegible]	lymphoid	5/1266	22/18723	0.014035	0.11653	0.099615	BATF/SPI1	5
BP	GO:003281	positive	5/1266	22/18723	0.014035	0.11653	0.099615	HLA-F/IL1	5
BP	GO:00508[illegible]	negative	5/1266	22/18723	0.014035	0.11653	0.099615	SLA2/GBP1	5
BP	GO:19001[illegible]	regulati	5/1266	22/18723	0.014035	0.11653	0.099615	PHLDA2/H[illegible]	5
BP	GO:00109[illegible]	positive	22/1266	197/1872[illegible]	0.014122	0.11682	0.099863	HIP1/PCO[illegible]	22
BP	GO:003241	lysosome	11/1266	77/18723	0.014134	0.11682	0.099863	MAP6/LYN/	11
BP	GO:005511	regulati	11/1266	77/18723	0.014134	0.11682	0.099863	PLN/CLIC[illegible]	11
BP	GO:20012[illegible]	positive	8/1266	48/18723	0.014338	0.118328	0.101152	PTPRC/TH[illegible]	8
BP	GO:00987[illegible]	detoxifi	18/1266	152/1872[illegible]	0.014388	0.118381	0.101197	APOM/CLI[illegible]	18
BP	GO:19013[illegible]	negative	18/1266	152/1872[illegible]	0.014388	0.118381	0.101197	SERPINE1/	18
BP	GO:00432[illegible]	regulati	23/1266	209/1872[illegible]	0.014472	0.118895	0.101636	HIP1/SLC1	23
BP	GO:00860[illegible]	regulati	7/1266	39/18723	0.014518	0.11891	0.101649	JUP/KCNE[illegible]	7
BP	GO:190311	regulati	7/1266	39/18723	0.014518	0.11891	0.101649	PLN/JUP/[illegible]	7
BP	GO:00715[illegible]	response	27/1266	256/1872[illegible]	0.014553	0.119021	0.101744	SOX5/PDE[illegible]	27
BP	GO:00326[illegible]	regulati	13/1266	98/18723	0.014628	0.11928	0.101965	CD84/HAV[illegible]	13
BP	GO:00613[illegible]	cardiac	13/1266	98/18723	0.014628	0.11928	0.101965	PLN/JUP/[illegible]	13
BP	GO:00516[illegible]	establis	38/1266	390/1872[illegible]	0.014972	0.121895	0.104201	UBXN2B/T[illegible]	38
BP	GO:00720[illegible]	nephron	14/1266	109/1872[illegible]	0.0151	0.122759	0.104939	LAMA5/IR[illegible]	14
BP	GO:00018[illegible]	kidney d	30/1266	293/1872[illegible]	0.015343	0.124235	0.106201	LRRK2/LA[illegible]	30
BP	GO:00508[illegible]	B cell r	16/1266	131/1872[illegible]	0.015471	0.124235	0.106201	LYN/NFAM1	16
BP	GO:001571	organic	35/1266	354/1872[illegible]	0.015504	0.124235	0.106201	SLC1A1/S[illegible]	35
BP	GO:00099[illegible]	cell-cell	10/1266	68/18723	0.015554	0.124235	0.106201	CD209/HA[illegible]	10
BP	GO:00024[illegible]	dendriti	4/1266	15/18723	0.015555	0.124235	0.106201	THBS1/CD[illegible]	4
BP	GO:00108[illegible]	negative	4/1266	15/18723	0.015555	0.124235	0.106201	TMEM119/[illegible]	4
BP	GO:00323[illegible]	alanine	4/1266	15/18723	0.015555	0.124235	0.106201	SLC38A2/[illegible]	4
BP	GO:00450[illegible]	T-helper	4/1266	15/18723	0.015555	0.124235	0.106201	BATF/IL1[illegible]	4
BP	GO:00600[illegible]	regulati	4/1266	15/18723	0.015555	0.124235	0.106201	ANO6/CD3[illegible]	4
BP	GO:00606[illegible]	regulati	4/1266	15/18723	0.015555	0.124235	0.106201	LRRK2/GA[illegible]	4
BP	GO:00860[illegible]	bundle o	4/1266	15/18723	0.015555	0.124235	0.106201	JUP/GJA5/	4
BP	GO:19017[illegible]	positive	4/1266	15/18723	0.015555	0.124235	0.106201	CXCL9/NF[illegible]	4
BP	GO:00513[illegible]	negative	37/1266	379/1872[illegible]	0.015747	0.125583	0.107354	PLN/LRRK[illegible]	37
BP	GO:00326[illegible]	chemokin	13/1266	99/18723	0.015832	0.126077	0.107775	CD84/HAV[illegible]	13
BP	GO:000271	positive	6/1266	31/18723	0.016145	0.12768	0.109146	HLA-F/LA[illegible]	6
BP	GO:00028[illegible]	regulati	6/1266	31/18723	0.016145	0.12768	0.109146	IL18/CD8[illegible]	6
BP	GO:00705[illegible]	protein	6/1266	31/18723	0.016145	0.12768	0.109146	MC1R/PLE[illegible]	6
BP	GO:00706[illegible]	transepi	6/1266	31/18723	0.016145	0.12768	0.109146	SLC1A1/P[illegible]	6
BP	GO:00027[illegible]	negative	8/1266	49/18723	0.01615	0.12768	0.109146	CTNNB1/L[illegible]	8
BP	GO:001491	regulati	12/1266	89/18723	0.016461	0.129761	0.110925	SERPINE1/	12
BP	GO:00463[illegible]	positive	12/1266	89/18723	0.016461	0.129761	0.110925	GADD45G/[illegible]	12
BP	GO:00067[illegible]	one-carb	7/1266	40/18723	0.016599	0.130142	0.111251	CA8/ALDH1	7
BP	GO:00512[illegible]	positive	7/1266	40/18723	0.016599	0.130142	0.111251	P2RY6/CX[illegible]	7
BP	GO:00434[illegible]	regulati	20/1266	177/1872[illegible]	0.016605	0.130142	0.111251	LRRK2/PI[illegible]	20
BP	GO:00987[illegible]	import a	20/1266	177/1872[illegible]	0.016605	0.130142	0.111251	SLC1A1/W[illegible]	20
BP	GO:00442[illegible]	cellular	29/1266	283/1872[illegible]	0.01676	0.130842	0.111849	TFF3/B3G[illegible]	29
BP	GO:00302[illegible]	lipid mo	23/1266	212/1872[illegible]	0.0169	0.130842	0.111849	INPP4B/I[illegible]	23
BP	GO:00060[illegible]	proteogl	11/1266	79/18723	0.016922	0.130842	0.111849	CTNNB1/S[illegible]	11
BP	GO:00028[illegible]	negative	9/1266	59/18723	0.016929	0.130842	0.111849	HLA-F/FO[illegible]	9
BP	GO:00023[illegible]	alpha-be	5/1266	23/18723	0.016958	0.130842	0.111849	FOXP3/SL[illegible]	5

BP	GO:00069:	substrate	5/1266	23/18723	0.016958	0.130842	0.111849	FN1/ROBO1	5
BP	GO:00301!	positive	5/1266	23/18723	0.016958	0.130842	0.111849	ANO6/SERI	5
BP	GO:00433(	CD4-posit	5/1266	23/18723	0.016958	0.130842	0.111849	FOXP3/SLA	5
BP	GO:00603(	regulatic	5/1266	23/18723	0.016958	0.130842	0.111849	GJA5/KCNI	5
BP	GO:00603{	innervati	5/1266	23/18723	0.016958	0.130842	0.111849	GABRA5/V(	5
BP	GO:19000/	positive	5/1266	23/18723	0.016958	0.130842	0.111849	ANO6/SERI	5
BP	GO:00018!	placenta	17/1266	144/1872:	0.01753	0.134873	0.115295	PHLDA2/PI	17
BP	GO:00103:	membrane	17/1266	144/1872:	0.01753	0.134873	0.115295	ANO6/MEGI	17
BP	GO:00072!	JNK casca	19/1266	167/1872:	0.017989	0.138125	0.118075	LRRK2/GAI	19
BP	GO:00164{	protein p	24/1266	225/1872:	0.018003	0.138125	0.118075	LRRK2/SEI	24
BP	GO:00512]	cartilage	21/1266	190/1872:	0.01803	0.138135	0.118084	LOXL2/CTN	21
BP	GO:00226(	regulatic	31/1266	309/1872:	0.018089	0.138397	0.118308	PLXNC1/SI	31
BP	GO:00431:	regulatic	26/1266	249/1872:	0.018263	0.139533	0.119279	BST2/CTNN	26
BP	GO:00015(	ossificat	39/1266	408/1872:	0.018347	0.139978	0.119659	ANO6/TCII	39
BP	GO:00171!	regulatic	22/1266	202/1872:	0.018402	0.140202	0.119851	RPH3AL/LI	22
BP	GO:00074:	periphera	11/1266	80/18723	0.018461	0.140457	0.120068	EGR2/ADGI	11
BP	GO:00362!	response	32/1266	322/1872:	0.018689	0.141583	0.121031	PAM/LOXL2	32
BP	GO:00198:	cytolysis	6/1266	32/18723	0.018765	0.141583	0.121031	GZMH/APOI	6
BP	GO:00199:	cGMP-medi	6/1266	32/18723	0.018765	0.141583	0.121031	PDE3A/CGA	6
BP	GO:00456{	positive	6/1266	32/18723	0.018765	0.141583	0.121031	KDF1/MACI	6
BP	GO:00508!	positive	6/1266	32/18723	0.018765	0.141583	0.121031	LMCD1/P2I	6
BP	GO:20001!	regulatic	6/1266	32/18723	0.018765	0.141583	0.121031	P2RX4/SYI	6
BP	GO:00510/	positive	31/1266	310/1872:	0.018845	0.141811	0.121226	RPH3AL/RA	31
BP	GO:00327:	positive	7/1266	41/18723	0.018883	0.141811	0.121226	LTB/SYK/1	7
BP	GO:00716:	regulatic	7/1266	41/18723	0.018883	0.141811	0.121226	FOXP3/FN1	7
BP	GO:00301(	platelet	15/1266	123/1872:	0.018899	0.141811	0.121226	LYN/PRKCA	15
BP	GO:00423!	regulatic	41/1266	434/1872:	0.018977	0.142196	0.121555	PLN/LRRK2	41
BP	GO:00109]	regulatic	4/1266	16/18723	0.019652	0.145852	0.12468	P2RY6/PLI	4
BP	GO:00456!	positive	4/1266	16/18723	0.019652	0.145852	0.12468	PRKCA/HCI	4
BP	GO:00702:	positive	4/1266	16/18723	0.019652	0.145852	0.12468	IDO1/CCL5	4
BP	GO:00713(	cellular	4/1266	16/18723	0.019652	0.145852	0.12468	SPI1/CYBI	4
BP	GO:00722{	metanephr	4/1266	16/18723	0.019652	0.145852	0.12468	CTNNB1/FM	4
BP	GO:19051!	regulatic	4/1266	16/18723	0.019652	0.145852	0.12468	ANO6/CD3(	4
BP	GO:20010/	regulatic	4/1266	16/18723	0.019652	0.145852	0.12468	EMP2/BST1	4
BP	GO:00071{	adenylate	17/1266	146/1872:	0.019827	0.146753	0.125451	PLN/PRKCA	17
BP	GO:00106'	regulatic	17/1266	146/1872:	0.019827	0.146753	0.125451	TFF3/P2RY	17
BP	GO:00435{	skin deve	27/1266	263/1872:	0.020014	0.147355	0.125965	LAMA5/COI	27
BP	GO:00140:	neural cr	11/1266	81/18723	0.020102	0.147355	0.125965	LAMA5/RAI	11
BP	GO:00305]	negative	11/1266	81/18723	0.020102	0.147355	0.125965	NRROS/ASI	11
BP	GO:00443/	cellular	14/1266	113/1872:	0.020158	0.147355	0.125965	CTNNB1/SM	14
BP	GO:00380!	Fc-epsilc	5/1266	24/18723	0.020258	0.147355	0.125965	LYN/SYK/V	5
BP	GO:00433(	positive	5/1266	24/18723	0.020258	0.147355	0.125965	HLA-F/SYI	5
BP	GO:00456/	regulatic	5/1266	24/18723	0.020258	0.147355	0.125965	PRKCA/HCI	5
BP	GO:00508:	positive	5/1266	24/18723	0.020258	0.147355	0.125965	ANO6/SERI	5
BP	GO:00605(	apoptotic	5/1266	24/18723	0.020258	0.147355	0.125965	SPI1/FOX(	5
BP	GO:00987/	cell aggr	5/1266	24/18723	0.020258	0.147355	0.125965	SOX5/MPZ/	5
BP	GO:20010:	regulatic	5/1266	24/18723	0.020258	0.147355	0.125965	SMOC2/P2I	5
BP	GO:00456(	regulatic	8/1266	51/18723	0.02026	0.147355	0.125965	NMRK2/CX(	8
BP	GO:00716:	regulatic	8/1266	51/18723	0.02026	0.147355	0.125965	RARRES2/1	8
BP	GO:00345!	cellular	29/1266	288/1872:	0.020743	0.150666	0.128796	LRRK2/CTN	29
BP	GO:00328(	response	27/1266	264/1872:	0.02091	0.151683	0.129665	PLN/LPIN1	27
BP	GO:00217!	cerebral	7/1266	42/18723	0.021377	0.154659	0.132209	CTNNB1/MI	7
BP	GO:20004(	regulatic	7/1266	42/18723	0.021377	0.154659	0.132209	SPN/CXCL1	7
BP	GO:00703'	positive	23/1266	217/1872:	0.021646	0.156298	0.13361	GPR183/C(	23

BP	GO:00341	regulatio	6/1266	33/18723	0.021661	0.156298	0.13361	LYN/PRKCA	6
BP	GO:00602	anatomica	31/1266	314/1872	0.02212	0.159401	0.136263	TCIRG1/TF	31
BP	GO:00513	response	17/1266	148/1872	0.022348	0.160831	0.137485	PAM/SPARC	17
BP	GO:00430	amino aci	8/1266	52/18723	0.022571	0.162218	0.138671	SLC1A1/SF	8
BP	GO:00081	actin pol	23/1266	218/1872	0.022708	0.162479	0.138894	ARFGEF1/F	23
BP	GO:00454	response	16/1266	137/1872	0.022746	0.162479	0.138894	VCAM1/SPA	16
BP	GO:00075	excretion	9/1266	62/18723	0.022845	0.162479	0.138894	EDNRB/SLC	9
BP	GO:00199	cAMP-medi	9/1266	62/18723	0.022845	0.162479	0.138894	PDE3A/API	9
BP	GO:00326	interleuk	9/1266	62/18723	0.022845	0.162479	0.138894	HLA-B/LTE	9
BP	GO:00326	regulatio	9/1266	62/18723	0.022845	0.162479	0.138894	HLA-B/LTE	9
BP	GO:00327	positive	9/1266	62/18723	0.022845	0.162479	0.138894	CD58/SERF	9
BP	GO:00461	polyol bi	9/1266	62/18723	0.022845	0.162479	0.138894	P2RY6/QDF	9
BP	GO:00328	positive	15/1266	126/1872	0.023017	0.163488	0.139756	GADD45G/S	15
BP	GO:00165	negative	17/1266	149/1872	0.023697	0.167913	0.143539	SERPINE1/	17
BP	GO:00486	response	11/1266	83/18723	0.023702	0.167913	0.143539	SLC1A1/LY	11
BP	GO:00030	renal sys	5/1266	25/18723	0.02395	0.168579	0.144109	GJA5/F2R/	5
BP	GO:00105	positive	5/1266	25/18723	0.02395	0.168579	0.144109	ARFGEF1/C	5
BP	GO:00315	membrane	5/1266	25/18723	0.02395	0.168579	0.144109	EMP2/PTPF	5
BP	GO:00428	defense r	5/1266	25/18723	0.02395	0.168579	0.144109	GBP4/BATF	5
BP	GO:00860	membrane	5/1266	25/18723	0.02395	0.168579	0.144109	ATP1B1/KC	5
BP	GO:00160	detection	4/1266	17/18723	0.024354	0.169021	0.144486	HLA-A/HLA	4
BP	GO:00326	granulocy	4/1266	17/18723	0.024354	0.169021	0.144486	CD84/SYK/	4
BP	GO:00326	regulatio	4/1266	17/18723	0.024354	0.169021	0.144486	CD84/SYK/	4
BP	GO:00336	regulatio	4/1266	17/18723	0.024354	0.169021	0.144486	RASIP1/PI	4
BP	GO:00420	regulatio	4/1266	17/18723	0.024354	0.169021	0.144486	COMT/SNCA	4
BP	GO:00420	regulatio	4/1266	17/18723	0.024354	0.169021	0.144486	COMT/SNCA	4
BP	GO:00468	inositol	4/1266	17/18723	0.024354	0.169021	0.144486	INPP4B/SY	4
BP	GO:00551	relaxatio	4/1266	17/18723	0.024354	0.169021	0.144486	PLN/ATP1E	4
BP	GO:00907	immunolog	4/1266	17/18723	0.024354	0.169021	0.144486	ST3GAL1/I	4
BP	GO:00989	regulatio	4/1266	17/18723	0.024354	0.169021	0.144486	NPTX2/NPT	4
BP	GO:19028	negative	4/1266	17/18723	0.024354	0.169021	0.144486	PPARG/HDA	4
BP	GO:00613	renal tub	12/1266	94/18723	0.024385	0.169021	0.144486	LAMA5/IRX	12
BP	GO:00359	cellular	10/1266	73/18723	0.024583	0.169959	0.145288	GAS1/VCAM	10
BP	GO:19044	positive	10/1266	73/18723	0.024583	0.169959	0.145288	P2RY6/ATF	10
BP	GO:00024	neutrophi	6/1266	34/18723	0.024844	0.171331	0.146461	DNASE1L3/	6
BP	GO:00860	cell-cell	6/1266	34/18723	0.024844	0.171331	0.146461	GJA5/SCN1	6
BP	GO:00487	skeletal	23/1266	220/1872	0.024954	0.171871	0.146922	CTNNB1/FN	23
BP	GO:00075	embryo in	8/1266	53/18723	0.025062	0.171963	0.147001	EMP2/SPP1	8
BP	GO:00457	negative	8/1266	53/18723	0.025062	0.171963	0.147001	APLNR/NEC	8
BP	GO:00713	cellular	8/1266	53/18723	0.025062	0.171963	0.147001	AQP1/PTAF	8
BP	GO:00019	regulatio	17/1266	150/1872	0.025106	0.17205	0.147076	MMP12/PXI	17
BP	GO:00073	gastrulat	20/1266	185/1872	0.025494	0.174274	0.148977	COL5A1/CT	20
BP	GO:00434	negative	20/1266	185/1872	0.025494	0.174274	0.148977	FOXS1/FOX	20
BP	GO:00712	cellular	21/1266	197/1872	0.025814	0.174888	0.149502	LRRK2/SLC	21
BP	GO:00316	negative	3/1266	10/18723	0.025839	0.174888	0.149502	LPIN1/TNF	3
BP	GO:00363	transform	3/1266	10/18723	0.025839	0.174888	0.149502	NRROS/LTE	3
BP	GO:00607	prostate	3/1266	10/18723	0.025839	0.174888	0.149502	SOX9/ESR1	3
BP	GO:00714	manganese	3/1266	10/18723	0.025839	0.174888	0.149502	SLC11A2/T	3
BP	GO:20002	positive	3/1266	10/18723	0.025839	0.174888	0.149502	KCNA5/MEC	3
BP	GO:20012	regulatio	3/1266	10/18723	0.025839	0.174888	0.149502	PTAFR/ATF	3
BP	GO:00607	regulatio	18/1266	162/1872	0.02584	0.174888	0.149502	MMP12/PXI	18
BP	GO:00324	regulatio	12/1266	95/18723	0.026257	0.177268	0.151536	MMP12/STI	12
BP	GO:00326	type I in	12/1266	95/18723	0.026257	0.177268	0.151536	MMP12/STI	12
BP	GO:00506	regulatio	36/1266	381/1872	0.026515	0.178785	0.152832	CTNNB1/SF	36

BP	GO:20001ξ	negative	17/1266	151/1872ξ	0.026577	0.178984	0.153003	SERPINE1/	17
BP	GO:19035ξ	positive	28/1266	282/1872ξ	0.026736	0.179802	0.153702	RPH3AL/RA	28
BP	GO:004647	phosphati	10/1266	74/18723	0.026764	0.179802	0.153702	SLC44A3/F	10
BP	GO:00326	interleuk	7/1266	44/18723	0.027034	0.181165	0.154867	FOXP3/SLA	7
BP	GO:00326(	regulatic	7/1266	44/18723	0.027034	0.181165	0.154867	FOXP3/SLA	7
BP	GO:00016(	response	30/1266	307/1872ξ	0.02719	0.181992	0.155574	PAM/LOXL2	30
BP	GO:00003(	response	23/1266	222/1872ξ	0.027366	0.182942	0.156386	LRRK2/IL1	23
BP	GO:00018	negative	34/1266	357/1872ξ	0.027509	0.183361	0.156744	BST2/HLA-	34
BP	GO:00308ξ	positive	9/1266	64/18723	0.027529	0.183361	0.156744	SERPINE1/	9
BP	GO:00705	response	9/1266	64/18723	0.027529	0.183361	0.156744	TBXAS1/FA	9
BP	GO:00320ξ	positive	11/1266	85/18723	0.02775	0.184542	0.157754	LRRK2/STI	11
BP	GO:00432ξ	positive	15/1266	129/1872ξ	0.027774	0.184542	0.157754	HIP1/SLCI	15
BP	GO:00015ξ	oocyte ma	5/1266	26/18723	0.02805	0.184798	0.157973	PDE3A/CD(	5
BP	GO:00015(	response	5/1266	26/18723	0.02805	0.184798	0.157973	GBP4/BATF	5
BP	GO:00034	endochonc	5/1266	26/18723	0.02805	0.184798	0.157973	SOX9/RARE	5
BP	GO:00722	metanephr	5/1266	26/18723	0.02805	0.184798	0.157973	CTNNB1/FM	5
BP	GO:19043	negative	5/1266	26/18723	0.02805	0.184798	0.157973	LZTFL1/LY	5
BP	GO:19055	positive	5/1266	26/18723	0.02805	0.184798	0.157973	RARRES2/F	5
BP	GO:20001(	positive	5/1266	26/18723	0.02805	0.184798	0.157973	IDO1/LYN/	5
BP	GO:00107(	positive	6/1266	35/18723	0.028324	0.185705	0.158748	WNK4/ATP1	6
BP	GO:00344(	response	6/1266	35/18723	0.028324	0.185705	0.158748	P2RX4/TFF	6
BP	GO:19035	release (	6/1266	35/18723	0.028324	0.185705	0.158748	PLN/CLIC2	6
BP	GO:20004(	positive	6/1266	35/18723	0.028324	0.185705	0.158748	SPN/CCL4/	6
BP	GO:00066	phospholi	36/1266	383/1872ξ	0.028392	0.185931	0.158942	LPIN1/SL(	36
BP	GO:00508ξ	cognitior	29/1266	296/1872ξ	0.028622	0.187211	0.160036	GABRA5/NF	29
BP	GO:00613ξ	renal tul	10/1266	75/18723	0.029081	0.189983	0.162405	LAMA5/IRX	10
BP	GO:00510ξ	positive	26/1266	260/1872ξ	0.029522	0.192277	0.164366	CAMK2A/CT	26
BP	GO:00022ξ	T-helper	4/1266	18/18723	0.029679	0.192277	0.164366	SLAMF6/BA	4
BP	GO:00090(	aspartate	4/1266	18/18723	0.029679	0.192277	0.164366	ASRGL1/RI	4
BP	GO:00442	cellular	4/1266	18/18723	0.029679	0.192277	0.164366	PYGM/AOAF	4
BP	GO:00468ξ	phosphory	4/1266	18/18723	0.029679	0.192277	0.164366	INPP4B/SY	4
BP	GO:00614ξ	hematopoi	4/1266	18/18723	0.029679	0.192277	0.164366	TCIRG1/GI	4
BP	GO:19000(	positive	4/1266	18/18723	0.029679	0.192277	0.164366	IQGAP1/LF	4
BP	GO:00329	regulatic	37/1266	397/1872ξ	0.029741	0.192454	0.164517	PAM/PLN/A	37
BP	GO:00140ξ	mesenchym	11/1266	86/18723	0.02995	0.193215	0.165168	LAMA5/RAI	11
BP	GO:00341(	regulatic	11/1266	86/18723	0.02995	0.193215	0.165168	PRKCA/INF	11
BP	GO:00458(	positive	35/1266	372/1872ξ	0.029965	0.193215	0.165168	HIP1/PCOI	35
BP	GO:00704ξ	response	33/1266	347/1872ξ	0.030062	0.193608	0.165504	PAM/LOXL2	33
BP	GO:00031	heart va	9/1266	65/18723	0.030108	0.193678	0.165564	MDM2/APLN	9
BP	GO:00344	lipid oxi	13/1266	108/1872ξ	0.030299	0.194678	0.166419	ACADVL/EF	13
BP	GO:00469	carboxyli	27/1266	273/1872ξ	0.030466	0.19504	0.166728	SLC1A1/SI	27
BP	GO:00070(	plasma me	16/1266	142/1872ξ	0.03057	0.19504	0.166728	ANO6/COL5	16
BP	GO:00720(	nephron (	16/1266	142/1872ξ	0.03057	0.19504	0.166728	LAMA5/IRX	16
BP	GO:00017ξ	neural cı	8/1266	55/18723	0.030606	0.19504	0.166728	LAMA5/RAI	8
BP	GO:00032	ventricul	8/1266	55/18723	0.030606	0.19504	0.166728	CHD7/ID2/	8
BP	GO:00480	inositol	8/1266	55/18723	0.030606	0.19504	0.166728	LMCD1/TB(	8
BP	GO:19055	macrophag	8/1266	55/18723	0.030606	0.19504	0.166728	RARRES2/F	8
BP	GO:00068(	lipid tra	37/1266	398/1872ξ	0.030737	0.195646	0.167246	ANO6/APOM	37
BP	GO:00016(	ameboidal	43/1266	475/1872ξ	0.031225	0.198523	0.169706	LAMA5/LOX	43
BP	GO:00083(	regulatic	17/1266	154/1872ξ	0.031379	0.199268	0.170343	PLXNC1/LS	17
BP	GO:00717	response	14/1266	120/1872ξ	0.031941	0.202604	0.173194	CTNNB1/SM	14
BP	GO:00096ξ	response	26/1266	262/1872ξ	0.032042	0.202732	0.173303	APOM/CLI(	26
BP	GO:00104(	negative	26/1266	262/1872ξ	0.032042	0.202732	0.173303	SERPINE1/	26
BP	GO:00706	response	6/1266	36/18723	0.03211	0.202732	0.173303	DCSTAMP/I	6

BP	GO:00903:	regulatio	6/1266	36/18723	0.03211	0.202732	0.173303	SYK/PON3/	6
BP	GO:19016(	alpha-ami	11/1266	87/18723	0.032272	0.203519	0.173976	IDO1/ASR(	11
BP	GO:00091(	glycoprot	36/1266	387/1872:	0.032457	0.203972	0.174363	B3GNT7/AI	36
BP	GO:00027	negative	5/1266	27/18723	0.032568	0.203972	0.174363	BST2/HLA-	5
BP	GO:00107!	regulatio	5/1266	27/18723	0.032568	0.203972	0.174363	RARRES2/T	5
BP	GO:00421(	positive	5/1266	27/18723	0.032568	0.203972	0.174363	IL18/IL12	5
BP	GO:00424(	cellular	5/1266	27/18723	0.032568	0.203972	0.174363	IDO1/COMT	5
BP	GO:00603:	interfer	5/1266	27/18723	0.032568	0.203972	0.174363	HCK/PPAR(	5
BP	GO:00301!	regulatio	9/1266	66/18723	0.032851	0.205324	0.175519	ANO6/SERI	9
BP	GO:00621!	cellular	32/1266	337/1872:	0.032859	0.205324	0.175519	LRRK2/CTN	32
BP	GO:00427	defense r	33/1266	350/1872:	0.033386	0.207257	0.177172	GBP4/SERI	33
BP	GO:20001(	negative	7/1266	46/18723	0.033629	0.207257	0.177172	HCLS1/TS(	7
BP	GO:00319(	response	18/1266	167/1872:	0.033665	0.207257	0.177172	PAM/SPAR(	18
BP	GO:00220:	telenceph	8/1266	56/18723	0.03367	0.207257	0.177172	LRRK2/CTN	8
BP	GO:00023:	immature	3/1266	11/18723	0.033777	0.207257	0.177172	SPI1/FNII	3
BP	GO:00026	positive	3/1266	11/18723	0.033777	0.207257	0.177172	FOXP3/CD:	3
BP	GO:00027:	negative	3/1266	11/18723	0.033777	0.207257	0.177172	HLA-F/FO)	3
BP	GO:00193	lipoxyger	3/1266	11/18723	0.033777	0.207257	0.177172	ALOX12/HI	3
BP	GO:00336:	positive	3/1266	11/18723	0.033777	0.207257	0.177172	RASIP1/PI	3
BP	GO:00456!	positive	3/1266	11/18723	0.033777	0.207257	0.177172	DCSTAMP/(	3
BP	GO:00465!	positive	3/1266	11/18723	0.033777	0.207257	0.177172	CD74/LGAI	3
BP	GO:00511:	NK T cell	3/1266	11/18723	0.033777	0.207257	0.177172	IL18/CD3(	3
BP	GO:00752!	positive	3/1266	11/18723	0.033777	0.207257	0.177172	CD74/LGAI	3
BP	GO:00860	SA node (	3/1266	11/18723	0.033777	0.207257	0.177172	GJA5/KCN/	3
BP	GO:00988	synapse p	3/1266	11/18723	0.033777	0.207257	0.177172	C1QA/C1QI	3
BP	GO:19023:	regulatio	3/1266	11/18723	0.033777	0.207257	0.177172	FOXC1/TNI	3
BP	GO:00024!	humoral i	14/1266	121/1872:	0.033964	0.207937	0.177753	PTPRC/C4/	14
BP	GO:00487(	embryonic	14/1266	121/1872:	0.033964	0.207937	0.177753	CTNNB1/DN	14
BP	GO:00991	regulatio	40/1266	440/1872:	0.034655	0.211868	0.181114	LRRK2/CAN	40
BP	GO:00301(	natural k	11/1266	88/18723	0.034719	0.211868	0.181114	HLA-F/IL1	11
BP	GO:00158:	peptide t	26/1266	264/1872:	0.034722	0.211868	0.181114	TCIRG1/T/	26
BP	GO:00109!	negative	25/1266	252/1872:	0.035099	0.213929	0.182876	SERPINE1/	25
BP	GO:00107!	positive	4/1266	19/18723	0.035637	0.214098	0.183019	RARRES2/T	4
BP	GO:00320	ARF prote	4/1266	19/18723	0.035637	0.214098	0.183019	ARFGEF1/I	4
BP	GO:00320	regulatio	4/1266	19/18723	0.035637	0.214098	0.183019	ARFGEF1/I	4
BP	GO:00326	interleuk	4/1266	19/18723	0.035637	0.214098	0.183019	IL18/HLA-	4
BP	GO:00326!	regulatio	4/1266	19/18723	0.035637	0.214098	0.183019	IL18/HLA-	4
BP	GO:00436!	engulfmer	4/1266	19/18723	0.035637	0.214098	0.183019	MEGF10/TI	4
BP	GO:00482	eosinophi	4/1266	19/18723	0.035637	0.214098	0.183019	CCL4/LGAI	4
BP	GO:00715	inositol	4/1266	19/18723	0.035637	0.214098	0.183019	INPP4B/SY	4
BP	GO:00860	atrial ca	4/1266	19/18723	0.035637	0.214098	0.183019	GJA5/KCN/	4
BP	GO:00860:	atrial ca	4/1266	19/18723	0.035637	0.214098	0.183019	GJA5/KCN/	4
BP	GO:00860(	atrial ca	4/1266	19/18723	0.035637	0.214098	0.183019	GJA5/KCN/	4
BP	GO:00976:	potassium	4/1266	19/18723	0.035637	0.214098	0.183019	ANO6/KCNI	4
BP	GO:20007:	positive	4/1266	19/18723	0.035637	0.214098	0.183019	SOX5/SOX9	4
BP	GO:00156!	inorganic	19/1266	180/1872:	0.035737	0.214461	0.18333	ANO6/GABI	19
BP	GO:00066:	fatty aci	36/1266	390/1872:	0.035789	0.214536	0.183394	PAM/LPIN1	36
BP	GO:00605:	muscle ti	37/1266	403/1872:	0.036106	0.216198	0.184815	SAP30/PLN	37
BP	GO:00456	regulatio	6/1266	37/18723	0.03621	0.216351	0.184945	MACROH2A2	6
BP	GO:00603(	regulatio	6/1266	37/18723	0.03621	0.216351	0.184945	GJA5/KCNI	6
BP	GO:00022(	myeloid (	17/1266	157/1872:	0.036794	0.219595	0.187719	LYN/INPP5	17
BP	GO:00069	activatio	10/1266	78/18723	0.036876	0.219846	0.187933	HIP1/SLC1	10
BP	GO:00610(	cell dif	8/1266	57/18723	0.036937	0.219967	0.188037	CTNNB1/WW	8
BP	GO:00458(	positive	12/1266	100/1872:	0.037212	0.221367	0.189234	HIP1/ANO6	12

BP	GO:00358	epitheli	7/1266	47/18723	0.037295	0.221473	0.189324	CTNNB1/WW	7
BP	GO:00104	regulati	5/1266	28/18723	0.037514	0.221473	0.189324	CTNNB1/PI	5
BP	GO:00326	interfer	5/1266	28/18723	0.037514	0.221473	0.189324	MMP12/HAV	5
BP	GO:00326	regulati	5/1266	28/18723	0.037514	0.221473	0.189324	MMP12/HAV	5
BP	GO:00456	negative	5/1266	28/18723	0.037514	0.221473	0.189324	CTNNB1/IN	5
BP	GO:00610	myeloid	5/1266	28/18723	0.037514	0.221473	0.189324	CD74/SPON	5
BP	GO:19030	positive	5/1266	28/18723	0.037514	0.221473	0.189324	ARFGEF1/C	5
BP	GO:00425	response	16/1266	146/1872	0.038134	0.224744	0.19212	LRRK2/IL1	16
BP	GO:00464	glyceroli	36/1266	392/1872	0.038151	0.224744	0.19212	LPIN1/SLC	36
BP	GO:00066	icosanoi	14/1266	123/1872	0.038284	0.225282	0.192581	MGST2/SYK	14
BP	GO:00074	axonogen	38/1266	418/1872	0.038693	0.227446	0.19443	MAP6/LAMA	38
BP	GO:00343	protein	9/1266	68/18723	0.038844	0.22784	0.194767	CTNNB1/CD	9
BP	GO:19000	regulati	9/1266	68/18723	0.038844	0.22784	0.194767	ANO6/SERP	9
BP	GO:00508	regulati	22/1266	218/1872	0.039106	0.229053	0.195804	LRRK2/LRP	22
BP	GO:00434	positive	13/1266	112/1872	0.039134	0.229053	0.195804	LRRK2/PIK	13
BP	GO:19016	response	20/1266	194/1872	0.039379	0.230239	0.196818	PLN/P2RY6	20
BP	GO:00435	positive	25/1266	255/1872	0.039582	0.231177	0.197619	ASAP1/ARA	25
BP	GO:00424	odontoge	11/1266	90/18723	0.039999	0.233366	0.19949	TCIRG1/LA	11
BP	GO:00324	positive	8/1266	58/18723	0.040409	0.235004	0.200891	MMP12/STI	8
BP	GO:00456	regulati	8/1266	58/18723	0.040409	0.235004	0.200891	KDF1/MACF	8
BP	GO:00860	cell com	8/1266	58/18723	0.040409	0.235004	0.200891	JUP/ATP1B	8
BP	GO:00107	macropha	6/1266	38/18723	0.040632	0.235544	0.201353	PPARG/IL1	6
BP	GO:00725	T-helper	6/1266	38/18723	0.040632	0.235544	0.201353	FOXP3/SLA	6
BP	GO:00900	foam cel	6/1266	38/18723	0.040632	0.235544	0.201353	PPARG/IL1	6
BP	GO:19016	alpha-am	20/1266	195/1872	0.041209	0.238164	0.203592	IDO1/DGLU	20
BP	GO:00429	xenobiot	7/1266	48/18723	0.041215	0.238164	0.203592	ABCB4/ABC	7
BP	GO:19030	regulati	7/1266	48/18723	0.041215	0.238164	0.203592	ANGPTL7/P	7
BP	GO:00072	I-kappaB	27/1266	281/1872	0.04148	0.238528	0.203904	BST2/CTNN	27
BP	GO:00066	glycerop	29/1266	306/1872	0.041494	0.238528	0.203904	LPIN1/SLC	29
BP	GO:00031	endothel	15/1266	136/1872	0.041646	0.238528	0.203904	CTNNB1/CO	15
BP	GO:00506	regulati	9/1266	69/18723	0.042102	0.238528	0.203904	MMP12/STI	9
BP	GO:00002	polysacc	4/1266	20/18723	0.042235	0.238528	0.203904	PYGM/AOAH	4
BP	GO:00028	regulati	4/1266	20/18723	0.042235	0.238528	0.203904	HAVCR2/MR	4
BP	GO:00028	regulati	4/1266	20/18723	0.042235	0.238528	0.203904	HAVCR2/MR	4
BP	GO:00171	plasma m	4/1266	20/18723	0.042235	0.238528	0.203904	ANO6/FASL	4
BP	GO:00316	negative	4/1266	20/18723	0.042235	0.238528	0.203904	LPIN1/TNF	4
BP	GO:00323	positive	4/1266	20/18723	0.042235	0.238528	0.203904	LOXL2/SOX	4
BP	GO:00436	linoleic	4/1266	20/18723	0.042235	0.238528	0.203904	ALOX12/CY	4
BP	GO:00602	mesenchy	4/1266	20/18723	0.042235	0.238528	0.203904	CTNNB1/FZ	4
BP	GO:00985	detectio	4/1266	20/18723	0.042235	0.238528	0.203904	HLA-A/HLA	4
BP	GO:20000	positive	4/1266	20/18723	0.042235	0.238528	0.203904	CD247/GPI	4
BP	GO:20010	positive	16/1266	148/1872	0.042386	0.238528	0.203904	HIP1/SLC1	16
BP	GO:00015	vasculog	10/1266	80/18723	0.042812	0.238528	0.203904	RASIP1/CT	10
BP	GO:00324	detectio	3/1266	12/18723	0.042823	0.238528	0.203904	TLR1/C4B/	3
BP	GO:00380	collagen-	3/1266	12/18723	0.042823	0.238528	0.203904	SYK/COL4A	3
BP	GO:00427	regulati	3/1266	12/18723	0.042823	0.238528	0.203904	COMT/SNCA	3
BP	GO:00433	regulati	3/1266	12/18723	0.042823	0.238528	0.203904	IL12RB1/H	3
BP	GO:00463	negative	3/1266	12/18723	0.042823	0.238528	0.203904	ACADVL/SC	3
BP	GO:00609	positive	3/1266	12/18723	0.042823	0.238528	0.203904	CD74/SPON	3
BP	GO:00711	response	3/1266	12/18723	0.042823	0.238528	0.203904	HDAC4/GJA	3
BP	GO:00716	negative	3/1266	12/18723	0.042823	0.238528	0.203904	FN1/LAPTM	3
BP	GO:00721	regulati	3/1266	12/18723	0.042823	0.238528	0.203904	CTNNB1/WW	3
BP	GO:19047	regulati	3/1266	12/18723	0.042823	0.238528	0.203904	FOXC1/TNF	3
BP	GO:00140	neural c	11/1266	91/18723	0.042838	0.238528	0.203904	LAMA5/RAI	11

BP	GO:00720	nephron t	11/1266	91/18723	0.042838	0.238528	0.203904	LAMA5/IRX	11
BP	GO:00356	multicell	17/1266	160/1872	0.042859	0.238528	0.203904	PLN/JUP/A	17
BP	GO:00028	negative	5/1266	29/18723	0.042896	0.238528	0.203904	LYN/HCK/S	5
BP	GO:00093	amine cat	5/1266	29/18723	0.042896	0.238528	0.203904	IDO1/COMT	5
BP	GO:00216	cranial r	5/1266	29/18723	0.042896	0.238528	0.203904	EGR2/MAFB	5
BP	GO:00217	olfactory	5/1266	29/18723	0.042896	0.238528	0.203904	LRRK2/ROB	5
BP	GO:00482	response	5/1266	29/18723	0.042896	0.238528	0.203904	P2RX4/EDN	5
BP	GO:00725	T-helper	5/1266	29/18723	0.042896	0.238528	0.203904	FOXP3/SLA	5
BP	GO:00988	bone grow	5/1266	29/18723	0.042896	0.238528	0.203904	SOX9/RARB	5
BP	GO:20004	positive	5/1266	29/18723	0.042896	0.238528	0.203904	SPN/CXCL1	5
BP	GO:00325	regulatio	35/1266	383/1872	0.042947	0.23857	0.20394	ANO6/ARFG	35
BP	GO:00308	regulatio	18/1266	172/1872	0.043113	0.239246	0.204517	ARFGEF1/F	18
BP	GO:00108	lipid loc	40/1266	448/1872	0.043912	0.243434	0.208097	ANO6/APOM	40
BP	GO:00316	response	42/1266	474/1872	0.044034	0.243693	0.208319	LRRK2/DAB	42
BP	GO:00218	forebrain	8/1266	59/18723	0.044093	0.243693	0.208319	LRRK2/CTN	8
BP	GO:00435	regulatio	8/1266	59/18723	0.044093	0.243693	0.208319	PIK3R6/SO	8
BP	GO:00147	striated	35/1266	384/1872	0.044309	0.24464	0.209128	SAP30/PLN	35
BP	GO:00108	regulatio	22/1266	221/1872	0.044394	0.244861	0.209318	SERPINE1/	22
BP	GO:00316	cellular	24/1266	246/1872	0.045132	0.248681	0.212582	LRRK2/DAB	24
BP	GO:00027	negative	6/1266	39/18723	0.04538	0.248859	0.212735	BST2/HLA-	6
BP	GO:00456	regulatio	6/1266	39/18723	0.04538	0.248859	0.212735	FOXP3/IL1	6
BP	GO:19027	apoptotic	6/1266	39/18723	0.04538	0.248859	0.212735	SPI1/MEGF	6
BP	GO:00140	response	7/1266	49/18723	0.045392	0.248859	0.212735	SLC1A1/RF	7
BP	GO:19020	regulatio	7/1266	49/18723	0.045392	0.248859	0.212735	SERPINE1/	7
BP	GO:00620	regulatio	31/1266	334/1872	0.045659	0.250071	0.213771	TFF3/ATPS	31
BP	GO:00351	appendage	15/1266	138/1872	0.046384	0.25353	0.216727	CTNNB1/FM	15
BP	GO:00351	limb morp	15/1266	138/1872	0.046384	0.25353	0.216727	CTNNB1/FM	15
BP	GO:00310	stress-ac	24/1266	247/1872	0.046923	0.25622	0.219027	LRRK2/GAD	24
BP	GO:00072	activatio	5/1266	30/18723	0.048717	0.264072	0.225739	P2RY6/S1P	5
BP	GO:00082	androgen	5/1266	30/18723	0.048717	0.264072	0.225739	ESR1/SPP1	5
BP	GO:00090	aromatic	5/1266	30/18723	0.048717	0.264072	0.225739	IDO1/QDPR	5
BP	GO:00171	stem cell	5/1266	30/18723	0.048717	0.264072	0.225739	NAP1L2/EV	5
BP	GO:00482	sperm cap	5/1266	30/18723	0.048717	0.264072	0.225739	CABYR/PAF	5
BP	GO:00511	regulatio	5/1266	30/18723	0.048717	0.264072	0.225739	FMN1/WAS/	5
BP	GO:00508	regulatio	21/1266	211/1872	0.048722	0.264072	0.225739	LRRK2/LRF	21
BP	GO:00192	regulatio	11/1266	93/18723	0.048924	0.264072	0.225739	ACADVL/PP	11
BP	GO:00603	bone morp	11/1266	93/18723	0.048924	0.264072	0.225739	TMEM119/S	11
BP	GO:00609	kidney mo	11/1266	93/18723	0.048924	0.264072	0.225739	LRRK2/LAM	11
BP	GO:00508	modulatio	39/1266	439/1872	0.049147	0.264072	0.225739	LRRK2/CAM	39
BP	GO:00335	multicell	9/1266	71/18723	0.049158	0.264072	0.225739	GABRA5/SI	9
BP	GO:00435	regulatio	9/1266	71/18723	0.049158	0.264072	0.225739	EEF1A2/PI	9
BP	GO:00508	regulatio	9/1266	71/18723	0.049158	0.264072	0.225739	ANO6/SERP	9
BP	GO:00015	cartilage	4/1266	21/18723	0.049474	0.264072	0.225739	SOX5/SOX9	4
BP	GO:00061	mitochond	4/1266	21/18723	0.049474	0.264072	0.225739	COX4I2/CO	4
BP	GO:00193	leukotrie	4/1266	21/18723	0.049474	0.264072	0.225739	MGST2/SYK	4
BP	GO:00343	diol bios	4/1266	21/18723	0.049474	0.264072	0.225739	QDPR/SPHK	4
BP	GO:00456	regulatio	4/1266	21/18723	0.049474	0.264072	0.225739	INPP5D/DC	4
BP	GO:00508	negative	4/1266	21/18723	0.049474	0.264072	0.225739	TBC1D10C/	4
BP	GO:00610	positive	4/1266	21/18723	0.049474	0.264072	0.225739	CD74/SPON	4
BP	GO:20002	regulatio	4/1266	21/18723	0.049474	0.264072	0.225739	KCNA5/MEG	4
BP	GO:20007	positive	4/1266	21/18723	0.049474	0.264072	0.225739	CGAS/ARG2	4
BP	GO:00509	sensory p	18/1266	175/1872	0.049623	0.264609	0.226199	GABRA5/SO	18
BP	GO:00019	positive	7/1266	50/18723	0.049832	0.265201	0.226705	MMP12/CD7	7
BP	GO:00026	positive	7/1266	50/18723	0.049832	0.265201	0.226705	PTPRC/HLA	7

CC	GO:00426	MHC prote	21/1323	25/19550	2.30E-21	1.36E-18	1.12E-18	HLA-A/HLA	21
CC	GO:00426	MHC class	14/1323	17/19550	2.22E-14	6.57E-12	5.40E-12	HLA-DMA/H	14
CC	GO:00098	external	72/1323	421/1955(	2.36E-13	3.34E-11	2.75E-11	ITGA1/CXC	72
CC	GO:00715	integral	17/1323	29/19550	2.82E-13	3.34E-11	2.75E-11	HLA-A/HLA	17
CC	GO:00985	lumenal s	17/1323	29/19550	2.82E-13	3.34E-11	2.75E-11	HLA-A/HLA	17
CC	GO:00985	lumenal s	17/1323	36/19550	2.97E-11	2.93E-09	2.40E-09	HLA-A/HLA	17
CC	GO:00620	collagen-	67/1323	425/1955(	6.91E-11	5.84E-09	4.80E-09	LAMA5/LOX	67
CC	GO:00451	membrane	56/1323	335/1955(	2.73E-10	1.80E-08	1.48E-08	LRRK2/ITC	56
CC	GO:00988	membrane	56/1323	335/1955(	2.73E-10	1.80E-08	1.48E-08	LRRK2/ITC	56
CC	GO:00306	secretory	52/1323	311/1955(	1.18E-09	6.96E-08	5.72E-08	ANO6/RPH3	52
CC	GO:00306	endocytic	35/1323	193/1955(	7.75E-08	4.12E-06	3.39E-06	TCIRG1/CA	35
CC	GO:00125	ER to Gol	18/1323	62/19550	8.36E-08	4.12E-06	3.39E-06	HLA-A/HLA	18
CC	GO:00058	trans-Gol	42/1323	259/1955(	1.11E-07	5.06E-06	4.16E-06	PAM/LRRK2	42
CC	GO:00017	immunolog	14/1323	44/19550	6.61E-07	2.80E-05	2.30E-05	HAVCR2/CI	14
CC	GO:00987	Golgi app	52/1323	384/1955(	1.32E-06	5.22E-05	4.29E-05	PAM/LRRK2	52
CC	GO:00453	phagocyti	26/1323	137/1955(	1.46E-06	5.39E-05	4.43E-05	TCIRG1/HI	26
CC	GO:00301	endocytic	47/1323	336/1955(	1.71E-06	5.94E-05	4.88E-05	TCIRG1/CA	47
CC	GO:00306	phagocyti	18/1323	76/19550	2.29E-06	7.52E-05	6.18E-05	TCIRG1/HI	18
CC	GO:00306	transport	33/1323	212/1955(	6.14E-06	0.000191	0.000157	RPH3AL/PA	33
CC	GO:00325	trans-Gol	20/1323	100/1955(	1.02E-05	0.000303	0.000249	CLIP3/HLA	20
CC	GO:00301	integral	27/1323	162/1955(	1.18E-05	0.000334	0.000274	HLA-A/EMC	27
CC	GO:00301	COPII-coa	19/1323	94/19550	1.44E-05	0.000389	0.000319	HLA-A/HLA	19
CC	GO:00988	plasma me	41/1323	306/1955(	2.18E-05	0.000562	0.000462	ITGA1/LYN	41
CC	GO:00312	intrinsic	27/1323	170/1955(	2.89E-05	0.000686	0.000563	HLA-A/EMC	27
CC	GO:00059	cell-cell	58/1323	494/1955(	2.90E-05	0.000686	0.000563	STEAP1/RA	58
CC	GO:00018	phagocyti	9/1323	28/19550	6.16E-05	0.001404	0.001153	MYO1G/MEC	9
CC	GO:00708	tertiary	25/1323	164/1955(	0.000111	0.002441	0.002005	ANO6/TCIR	25
CC	GO:00057	endoplasm	39/1323	313/1955(	0.000167	0.003533	0.002902	COL5A1/P4	39
CC	GO:00430	NADPH oxi	6/1323	14/19550	0.000178	0.003627	0.00298	NCF4/CYBB	6
CC	GO:00055	collagen	16/1323	87/19550	0.000216	0.00426	0.0035	COL5A1/CO	16
CC	GO:00021	podosome	8/1323	29/19550	0.000512	0.009784	0.008037	VCAM1/ASA	8
CC	GO:00056	basement	16/1323	96/19550	0.00068	0.012374	0.010165	LAMA5/LOX	16
CC	GO:01010	ficolin-1	12/1323	61/19550	0.00069	0.012374	0.010165	TCIRG1/CI	12
CC	GO:00448	plasma me	18/1323	116/1955(	0.000782	0.013617	0.011186	LRRK2/CTN	18
CC	GO:00985	cytoplasm	26/1323	197/1955(	0.000824	0.013719	0.01127	HIP1/LRRK	26
CC	GO:00083	integrin	8/1323	31/19550	0.000834	0.013719	0.01127	ITGA1/LYN	8
CC	GO:00301	transport	45/1323	412/1955(	0.001036	0.016313	0.013401	RPH3AL/PA	45
CC	GO:00059	gap junct	8/1323	32/19550	0.001047	0.016313	0.013401	GJA5/GJB1	8
CC	GO:00708	tertiary	13/1323	73/19550	0.001122	0.017038	0.013996	ANO6/ITGA	13
CC	GO:00306	coated ve	24/1323	181/1955(	0.001201	0.017775	0.014602	HIP1/HLA-	24
CC	GO:00550	recycling	15/1323	92/19550	0.001246	0.017988	0.014776	HLA-A/SLC	15
CC	GO:00319	early end	23/1323	173/1955(	0.00145	0.02044	0.016791	KREMEN2/H	23
CC	GO:00057	vacuolar	47/1323	444/1955(	0.00156	0.021477	0.017643	TCIRG1/BS	47
CC	GO:00313	integral	42/1323	387/1955(	0.00169	0.022732	0.018674	HLA-A/EMC	42
CC	GO:00451	apical pa	46/1323	435/1955(	0.001775	0.023355	0.019185	TCIRG1/BS	46
CC	GO:00059	connexin	6/1323	21/19550	0.00213	0.026833	0.022043	GJA5/GJB1	6
CC	GO:00986	complex (	6/1323	21/19550	0.00213	0.026833	0.022043	COL5A1/CO	6
CC	GO:00165	sarcoplas	13/1323	79/19550	0.002358	0.028597	0.023492	PLN/FLNC/	13
CC	GO:00986	protein (	8/1323	36/19550	0.002367	0.028597	0.023492	ITGA1/LYN	8
CC	GO:00098	cytoplasm	22/1323	172/1955(	0.002964	0.035099	0.028833	HIP1/RASA	22
CC	GO:00306	clathrin-	12/1323	72/19550	0.003064	0.035568	0.029219	HLA-DPA1/	12
CC	GO:00198	extrinsic	34/1323	309/1955(	0.003563	0.040559	0.033318	HIP1/CTNN	34
CC	GO:00058	actin fil	16/1323	114/1955(	0.004231	0.045636	0.037489	FMN1/WAS/	16
CC	GO:00057	lysosomal	41/1323	395/1955(	0.00424	0.045636	0.037489	TCIRG1/BS	41

CC	GO:00988	lytic vac	41/1323	395/1955(	0.00424	0.045636	0.037489	TCIRG1/BS	41
CC	GO:00421(	T cell re	19/1323	148/1955(	0.005288	0.055901	0.045922	TRBV4-2/(	19
CC	GO:00347(	chloride	9/1323	50/19550	0.005833	0.060586	0.04977	ANO6/GABI	9
CC	GO:00313(	intrinsic	42/1323	417/1955(	0.00646	0.064603	0.05307	HLA-A/EM(	42
CC	GO:00550	recycling	23/1323	195/1955(	0.006617	0.064603	0.05307	HLA-A/RAI	23
CC	GO:00055	fibrillar	4/1323	12/19550	0.006657	0.064603	0.05307	COL5A1/C(	4
CC	GO:00986	banded co	4/1323	12/19550	0.006657	0.064603	0.05307	COL5A1/C(	4
CC	GO:00057(	early enc	40/1323	394/1955(	0.006788	0.064816	0.053245	KREMEN2/I	40
CC	GO:00165	sarcoplas	11/1323	70/19550	0.007071	0.06644	0.054579	PLN/THBS1	11
CC	GO:00602(	cytoplasm	34/1323	325/1955(	0.007703	0.071252	0.058532	SERPINE1/	34
CC	GO:00310	platelet	13/1323	91/19550	0.008108	0.073849	0.060665	SERPINE1/	13
CC	GO:00319	vesicle l	34/1323	327/1955(	0.008428	0.075235	0.061804	SERPINE1/	34
CC	GO:00300	cell-subs	42/1323	425/1955(	0.008865	0.075235	0.061804	ITGA1/CTN	42
CC	GO:00019	uropod	4/1323	13/19550	0.009106	0.075235	0.061804	SPN/PSTPI	4
CC	GO:00312	cell trai	4/1323	13/19550	0.009106	0.075235	0.061804	SPN/PSTPI	4
CC	GO:00343(	low-densi	4/1323	13/19550	0.009106	0.075235	0.061804	APOM/MSR1	4
CC	GO:00017	ruffle	21/1323	178/1955(	0.009153	0.075235	0.061804	FRMD4B/AI	21
CC	GO:00057	mitochonc	5/1323	20/19550	0.009277	0.075235	0.061804	COX4I2/C(	5
CC	GO:00164	vacuolar	5/1323	20/19550	0.009277	0.075235	0.061804	TCIRG1/AT	5
CC	GO:00163	apical pl	37/1323	367/1955(	0.009966	0.079728	0.065495	TCIRG1/BS	37
CC	GO:00057	multivesi	10/1323	64/19550	0.010387	0.081986	0.06735	LRRK2/BST	10
CC	GO:00059(	caveola	12/1323	84/19550	0.010725	0.083373	0.068489	LRRK2/CTN	12
CC	GO:00059	focal adh	41/1323	418/1955(	0.010844	0.083373	0.068489	ITGA1/CTN	41
CC	GO:00347	secretory	33/1323	322/1955(	0.011443	0.085599	0.070318	SERPINE1/	33
CC	GO:00428	platelet	5/1323	21/19550	0.011511	0.085599	0.070318	RARRES2/A	5
CC	GO:00306(	clathrin-	15/1323	116/1955(	0.011567	0.085599	0.070318	HIP1/HLA-	15
CC	GO:00310	platelet	4/1323	14/19550	0.012076	0.088258	0.072502	RARRES2/(	4
CC	GO:00431	dendritic	21/1323	183/1955(	0.012333	0.08904	0.073144	CAMK2A/SI	21
CC	GO:00443(	neuron sp	21/1323	184/1955(	0.013064	0.092075	0.075638	CAMK2A/SI	21
CC	GO:00301	filopodiu	14/1323	107/1955(	0.013065	0.092075	0.075638	VCAM1/MY(	14
CC	GO:00300	Z disc	16/1323	130/1955(	0.014574	0.101507	0.083386	CTNNB1/FI	16
CC	GO:00057	late endc	29/1323	282/1955(	0.016233	0.110462	0.090742	TCIRG1/LI	29
CC	GO:00347(	ion chanr	29/1323	282/1955(	0.016233	0.110462	0.090742	ANO6/GABI	29
CC	GO:00347(	potassium	12/1323	89/19550	0.016567	0.110897	0.091099	KCNA5/KCN	12
CC	GO:00330	sarcoplas	7/1323	40/19550	0.016672	0.110897	0.091099	PLN/ITPR2	7
CC	GO:00080	voltage-g	11/1323	79/19550	0.017025	0.111986	0.091995	KCNA5/KCN	11
CC	GO:00147(	intercala	8/1323	50/19550	0.018209	0.118456	0.097309	CTNNB1/JU	8
CC	GO:00300	lamellipo	22/1323	202/1955(	0.01858	0.11956	0.098216	CTNNB1/AI	22
CC	GO:00453	clathrin-	12/1323	91/19550	0.019478	0.122811	0.100886	HLA-DPA1/	12
CC	GO:00312	cell leac	40/1323	422/1955(	0.019612	0.122811	0.100886	GABRA5/CT	40
CC	GO:00325	multivesi	4/1323	16/19550	0.019708	0.122811	0.100886	ABCA3/LAI	4
CC	GO:00312	anchored	19/1323	170/1955(	0.021477	0.132443	0.108799	BST2/GAS1	19
CC	GO:00425	specific	18/1323	160/1955(	0.023317	0.142307	0.116902	ANO6/JUP/	18
CC	GO:00310	platelet	4/1323	17/19550	0.024422	0.14753	0.121193	SPARC/PE(	4
CC	GO:00466	anchored	9/1323	63/19550	0.025236	0.150301	0.123469	GAS1/MDGA	9
CC	GO:01010(	ficolin-l	20/1323	185/1955(	0.025715	0.150301	0.123469	TCIRG1/CI	20
CC	GO:00056	nuclear e	3/1323	10/19550	0.025896	0.150301	0.123469	BCHE/PLAA	3
CC	GO:00059	fascia ac	3/1323	10/19550	0.025896	0.150301	0.123469	CTNNB1/JU	3
CC	GO:00988	actin-bas	23/1323	221/1955(	0.026386	0.151653	0.12458	LRRK2/CTN	23
CC	GO:00331	proton-tr	5/1323	26/19550	0.028141	0.160189	0.131592	TCIRG1/AT	5
CC	GO:00316	I band	16/1323	141/1955(	0.029076	0.163933	0.134668	CTNNB1/FI	16
CC	GO:00432(	lysosomal	12/1323	97/19550	0.030493	0.170302	0.1399	HYAL1/GP(	12
CC	GO:00343	plasma li	6/1323	36/19550	0.032228	0.174645	0.143468	APOM/APOI	6
CC	GO:00431	dendritic	6/1323	36/19550	0.032228	0.174645	0.143468	SLC1A1/MA	6

CC	GO:199077	lipoprote	6/1323	36/19550	0.032228	0.174645	0.143468	APOM/APOI	6
CC	GO:003013	coated ve	29/1323	299/19550	0.032451	0.174645	0.143468	HIP1/HLA-	29
CC	GO:001645	myosin co	8/1323	56/19550	0.033821	0.175783	0.144402	MYO1G/MYO	8
CC	GO:000579	Golgi med	3/1323	11/19550	0.03385	0.175783	0.144402	HLA-A/MAN	3
CC	GO:001977	proteasom	3/1323	11/19550	0.03385	0.175783	0.144402	PSMB8/PSM	3
CC	GO:003109	platelet	3/1323	11/19550	0.03385	0.175783	0.144402	F2R/ITPR2	3
CC	GO:003258	ruffle me	12/1323	99/19550	0.035004	0.180192	0.148024	PSD2/FAP/	12
CC	GO:003109	platelet	9/1323	67/19550	0.035933	0.18338	0.150643	SERPINE1/	9
CC	GO:004527	respirato	5/1323	28/19550	0.037633	0.190416	0.156423	COX4I2/CO	5
CC	GO:003190	late endo	16/1323	146/19550	0.0384	0.191033	0.15693	SLC1A1/HI	16
CC	GO:007256	blood mic	16/1323	146/19550	0.0384	0.191033	0.15693	BCHE/FN1/	16
CC	GO:003123	extrinsic	12/1323	101/19550	0.039968	0.197177	0.161976	HIP1/LYN/	12
CC	GO:003152	brush bor	8/1323	58/19550	0.040585	0.198565	0.163117	SLC11A2/A	8
CC	GO:000583	proteasom	4/1323	20/19550	0.042347	0.200753	0.164914	PSMB8/PSM	4
CC	GO:003436	very-low-	4/1323	20/19550	0.042347	0.200753	0.164914	APOM/APOI	4
CC	GO:003438	triglycer	4/1323	20/19550	0.042347	0.200753	0.164914	APOM/APOI	4
CC	GO:004325	laminin c	3/1323	12/19550	0.042915	0.200753	0.164914	LAMA5/LAM	3
CC	GO:000590	microvill	11/1323	91/19550	0.043067	0.200753	0.164914	LRRK2/CTN	11
CC	GO:003557	specific	11/1323	91/19550	0.043067	0.200753	0.164914	ANO6/LAIR	11
CC	GO:003299	protein-l	6/1323	39/19550	0.045539	0.210619	0.173019	APOM/APOI	6
CC	GO:004238	sarcolemm	15/1323	138/19550	0.046684	0.214238	0.175992	VCAM1/AHN	15
CC	GO:000577	vacuolar	18/1323	174/19550	0.047724	0.217329	0.178531	FABP5/HYA	18
MF	GO:002302	MHC prote	19/1275	36/18368	2.35E-13	2.41E-10	2.14E-10	CYRIB/HLA	19
MF	GO:002302	MHC class	15/1275	27/18368	3.04E-11	1.50E-08	1.34E-08	HLA-DMA/A	15
MF	GO:004260	peptide a	17/1275	36/18368	4.41E-11	1.50E-08	1.34E-08	HLA-A/TAP	17
MF	GO:003239	MHC class	8/1275	10/18368	2.09E-08	5.35E-06	4.76E-06	HLA-DPA1/	8
MF	GO:004260	T cell re	8/1275	11/18368	7.21E-08	1.47E-05	1.31E-05	HLA-A/CD3	8
MF	GO:005084	extracell	17/1275	56/18368	1.33E-07	2.26E-05	2.01E-05	SPARC/SMO	17
MF	GO:014037	immune re	29/1275	144/18368	1.66E-07	2.41E-05	2.14E-05	CXCR6/CCR	29
MF	GO:003802	cargo rec	20/1275	77/18368	1.88E-07	2.41E-05	2.14E-05	LOXL2/CD1	20
MF	GO:000504	scavenger	14/1275	47/18368	2.19E-06	0.000248	0.000221	LOXL2/CD1	14
MF	GO:000551	collagen	17/1275	69/18368	3.41E-06	0.000349	0.00031	PCOLCE2/1	17
MF	GO:000517	integrin	26/1275	144/18368	6.03E-06	0.000561	0.000499	LAMA5/COL	26
MF	GO:000520	extracell	29/1275	172/18368	7.32E-06	0.000624	0.000555	LAMA5/COL	29
MF	GO:001995	cytokine	25/1275	139/18368	9.70E-06	0.000756	0.000672	CXCR6/CCR	25
MF	GO:004323	laminin b	10/1275	28/18368	1.03E-05	0.000756	0.000672	THBS1/GPC	10
MF	GO:001617	superoxic	6/1275	10/18368	1.82E-05	0.001242	0.001104	NCF4/NCF1	6
MF	GO:001983	growth fa	24/1275	141/18368	3.73E-05	0.002382	0.002118	COL5A1/IG	24
MF	GO:004216	SH2 domai	11/1275	40/18368	6.12E-05	0.003683	0.003274	SYK/AFAP1	11
MF	GO:000552	insulin-l	9/1275	29/18368	0.000102	0.005807	0.005162	IGFBPL1/I	9
MF	GO:000382	antigen b	26/1275	171/18368	0.000128	0.006917	0.006149	HLA-A/IGI	26
MF	GO:003321	amide bir	48/1275	400/18368	0.000146	0.007482	0.006652	HLA-A/TAP	48
MF	GO:000377	actin bir	51/1275	441/18368	0.00023	0.010563	0.009391	HIP1/LRRK	51
MF	GO:004227	peptide b	40/1275	321/18368	0.000233	0.010563	0.009391	HLA-A/TAP	40
MF	GO:001712	SH3 domai	21/1275	130/18368	0.000237	0.010563	0.009391	FMN1/WAS/	21
MF	GO:000503	tumor nec	5/1275	10/18368	0.0003	0.012273	0.010911	TNFRSF18/	5
MF	GO:004320	glycosphi	5/1275	10/18368	0.0003	0.012273	0.010911	CLIP3/LYN	5
MF	GO:000495	icosanoic	6/1275	15/18368	0.000321	0.012614	0.011214	PTGER1/PT	6
MF	GO:003002	extracell	10/1275	41/18368	0.000386	0.014621	0.012999	COL5A1/CO	10
MF	GO:000820	heparin b	24/1275	166/18368	0.000491	0.01795	0.015958	PCOLCE2/C	24
MF	GO:002288	serine tr	5/1275	11/18368	0.000518	0.018289	0.01626	SLC38A2/S	5
MF	GO:001526	channel a	53/1275	480/18368	0.000541	0.018445	0.016398	STEAP1/AN	53
MF	GO:002280	passive t	53/1275	481/18368	0.000568	0.018745	0.016665	STEAP1/AN	53
MF	GO:001517	amino aci	15/1275	84/18368	0.000609	0.019155	0.01703	SLC38A8/S	15

MF	GO:00433	proteogly	9/1275	36/18368	0.000618	0.019155	0.01703	COL5A1/FN	9
MF	GO:00228	wide pore	8/1275	30/18368	0.000777	0.023392	0.020796	GJA5/GJB	8
MF	GO:00151	neutral a	9/1275	38/18368	0.000944	0.027583	0.024522	SLC1A1/SL	9
MF	GO:00051	tumor nec	8/1275	31/18368	0.000985	0.027985	0.024879	CD70/LTB/	8
MF	GO:00510	actin fil	28/1275	217/1836	0.001115	0.030842	0.027419	HIP1/RCSI	28
MF	GO:00050	death rec	5/1275	13/18368	0.001284	0.034577	0.03074	TNFRSF18/	5
MF	GO:00055	glycosami	29/1275	230/1836	0.001322	0.034683	0.030834	PCOLCE2/C	29
MF	GO:00422	MHC prote	9/1275	40/18368	0.001397	0.035717	0.031753	CYRIB/TAF	9
MF	GO:00162	antioxida	14/1275	86/18368	0.002293	0.055098	0.048983	APOM/CLIC	14
MF	GO:00055	phospholi	49/1275	466/1836	0.002406	0.055098	0.048983	HIP1/APOM	49
MF	GO:00016	purinergi	6/1275	21/18368	0.002424	0.055098	0.048983	GPR34/P2R	6
MF	GO:00052	gap junct	6/1275	21/18368	0.002424	0.055098	0.048983	GJA5/GJB	6
MF	GO:00165	nucleotic	6/1275	21/18368	0.002424	0.055098	0.048983	GPR34/P2R	6
MF	GO:00327	GTPase ac	5/1275	15/18368	0.002666	0.059298	0.052717	PLCD1/GNA	5
MF	GO:00048	cytokine	15/1275	97/18368	0.002734	0.059515	0.05291	CXCR6/CCR	15
MF	GO:00017	phosphoty	9/1275	44/18368	0.00282	0.060101	0.053432	HCK/SYK/V	9
MF	GO:00433	phosphati	7/1275	29/18368	0.003077	0.062948	0.055962	HIP1/ARAF	7
MF	GO:00466	sphingoli	7/1275	29/18368	0.003077	0.062948	0.055962	CLIP3/LYN	7
MF	GO:00049	prostagla	4/1275	10/18368	0.003456	0.069329	0.061636	PTGER1/PT	4
MF	GO:00166	oxidoredu	5/1275	16/18368	0.003659	0.071981	0.063993	LOXL2/VCA	5
MF	GO:00150	proton tr	18/1275	130/1836	0.003802	0.073395	0.06525	TCIRG1/AT	18
MF	GO:01403	intrameml	8/1275	38/18368	0.003961	0.075042	0.066714	ANO6/ABCE	8
MF	GO:00080	microtubu	31/1275	271/1836	0.004242	0.078903	0.070147	LRRK2/MAF	31
MF	GO:00228	gated cha	37/1275	340/1836	0.004432	0.080969	0.071983	ANO6/GABF	37
MF	GO:00001	microfila	7/1275	31/18368	0.004591	0.082389	0.073246	MYO1G/MYC	7
MF	GO:00052	chloride	12/1275	74/18368	0.004731	0.08345	0.07419	ANO6/GABF	12
MF	GO:00049	prostanoi	4/1275	11/18368	0.005135	0.088673	0.078832	PTGER1/PT	4
MF	GO:00150	corecepto	9/1275	48/18368	0.005201	0.088673	0.078832	CXCR6/ACK	9
MF	GO:19016	sulfur co	30/1275	265/1836	0.005626	0.094345	0.083875	PCOLCE2/C	30
MF	GO:00080	chemokine	9/1275	49/18368	0.005987	0.098791	0.087828	CXCL11/CC	9
MF	GO:00051	Notch bin	6/1275	25/18368	0.006232	0.09961	0.088556	MEGF10/DL	6
MF	GO:00302	polysacch	6/1275	25/18368	0.006232	0.09961	0.088556	ENPP2/PPF	6
MF	GO:00302	lipoprote	5/1275	18/18368	0.00639	0.100567	0.089406	LRP8/VLDL	5
MF	GO:00085	anion tra	34/1275	314/1836	0.006674	0.102792	0.091385	ANO6/GABF	34
MF	GO:00052	anion cha	13/1275	87/18368	0.006827	0.102792	0.091385	ANO6/GABF	13
MF	GO:00052	ion chann	44/1275	432/1836	0.006858	0.102792	0.091385	ANO6/GABF	44
MF	GO:00082	serine-ty	23/1275	191/1836	0.006933	0.102792	0.091385	MMP12/GZM	23
MF	GO:00512	phosphopr	13/1275	88/18368	0.007516	0.107703	0.09575	LYN/HCK/S	13
MF	GO:00996	ligand-ga	6/1275	26/18368	0.007634	0.107703	0.09575	RASA3/P2R	6
MF	GO:00302	carbohydr	30/1275	271/1836	0.007686	0.107703	0.09575	PAM/LOXL2	30
MF	GO:00350	phosphati	30/1275	271/1836	0.007686	0.107703	0.09575	HIP1/PLCE	30
MF	GO:00425	phosphori	38/1275	366/1836	0.008481	0.117246	0.104235	LPIN1/LRF	38
MF	GO:00151	L-amino a	10/1275	61/18368	0.008807	0.119013	0.105806	SLC1A1/SL	10
MF	GO:00171	serine hy	23/1275	195/1836	0.008842	0.119013	0.105806	MMP12/GZM	23
MF	GO:00042	serine-ty	21/1275	174/1836	0.00935	0.122516	0.10892	MMP12/GZM	21
MF	GO:00085	organic a	22/1275	185/1836	0.009389	0.122516	0.10892	SLC1A1/SL	22
MF	GO:00042	metalloen	15/1275	111/1836	0.009739	0.122516	0.10892	MMP12/TRA	15
MF	GO:01060	phosphati	4/1275	13/18368	0.00995	0.122516	0.10892	INPP4B/IN	4
MF	GO:01403	glyceroph	4/1275	13/18368	0.00995	0.122516	0.10892	ABCB4/ATF	4
MF	GO:00506	NADP binc	9/1275	53/18368	0.010081	0.122516	0.10892	CBR3/ME1/	9
MF	GO:00152	ligand-ga	18/1275	143/1836	0.0102	0.122516	0.10892	GABRA5/RA	18
MF	GO:00228	ligand-ga	18/1275	143/1836	0.0102	0.122516	0.10892	GABRA5/RA	18
MF	GO:00166	oxidoredu	5/1275	20/18368	0.010299	0.122516	0.10892	LOXL2/VCA	5
MF	GO:00422	MHC class	5/1275	20/18368	0.010299	0.122516	0.10892	TAP1/CD8A	5

MF	GO:00990	ligand-ga	15/1275	113/1836	0.011404	0.134096	0.119215	RASA3/P2F	15
MF	GO:00046	phosphol	14/1275	104/1836	0.012652	0.146676	0.130399	PLCD1/CCF	14
MF	GO:00018	complemer	5/1275	21/18368	0.012761	0.146676	0.130399	MEGF10/C4	5
MF	GO:00015	amyloid-b	12/1275	84/18368	0.012925	0.146918	0.130614	BCHE/PFDN	12
MF	GO:00450	G protein	4/1275	14/18368	0.013176	0.148122	0.131684	GPR34/P2F	4
MF	GO:00051	cytokine	29/1275	271/1836	0.013459	0.149657	0.133049	CD70/SOCS	29
MF	GO:00453	protein p	9/1275	56/18368	0.014321	0.157528	0.140046	HCK/SYK/V	9
MF	GO:00052	intracell	6/1275	30/18368	0.015542	0.169146	0.150375	RASA3/AQF	6
MF	GO:00167	phosphata	29/1275	275/1836	0.016174	0.174168	0.15484	LPIN1/LRF	29
MF	GO:00055	phosphati	7/1275	39/18368	0.016573	0.174694	0.155307	ARHGAP9/A	7
MF	GO:00018	opsonin b	4/1275	15/18368	0.016998	0.174694	0.155307	MEGF10/C4	4
MF	GO:00050	low-densi	4/1275	15/18368	0.016998	0.174694	0.155307	LRP8/VLDL	4
MF	GO:00860	voltage-g	4/1275	15/18368	0.016998	0.174694	0.155307	KCNE4/KCN	4
MF	GO:19019	phosphati	20/1275	173/1836	0.017077	0.174694	0.155307	HIP1/PLCD	20
MF	GO:00469	carboxyli	19/1275	163/1836	0.018236	0.184703	0.164206	SLC38A8/S	19
MF	GO:00328	tumor nec	8/1275	49/18368	0.01864	0.186819	0.166087	CD70/LTB/	8
MF	GO:00164	C-C chemo	5/1275	23/18368	0.01881	0.186819	0.166087	CXCR6/CCF	5
MF	GO:00053	organic a	19/1275	164/1836	0.019331	0.19015	0.169049	SLC38A8/S	19
MF	GO:00050	GTPase ac	44/1275	462/1836	0.020477	0.199299	0.177182	LRRK2/ARF	44
MF	GO:00480	receptor	46/1275	487/1836	0.020651	0.199299	0.177182	CD70/ASIF	46
MF	GO:01403	flippase	4/1275	16/18368	0.021444	0.205021	0.182269	ABCB4/ATF	4
MF	GO:00443	transmemb	16/1275	133/1836	0.021992	0.206515	0.183597	LRRK2/CTN	16
MF	GO:00051	cytokine	25/1275	235/1836	0.022004	0.206515	0.183597	CD70/CXCL	25
MF	GO:00152	solute:pr	5/1275	24/18368	0.022437	0.206784	0.183836	SLC11A2/M	5
MF	GO:00199	C-C chemo	5/1275	24/18368	0.022437	0.206784	0.183836	CXCR6/CCF	5
MF	GO:00046	peroxidas	8/1275	51/18368	0.023311	0.211063	0.187641	CLIC2/MGS	8
MF	GO:00082	metallope	21/1275	190/1836	0.023314	0.211063	0.187641	MMP12/TRA	21
MF	GO:00055	copper io	9/1275	61/18368	0.02411	0.21435	0.190562	PAM/LOXL2	9
MF	GO:00041	endopepti	41/1275	430/1836	0.024187	0.21435	0.190562	MMP12/TRA	41
MF	GO:00015	lipopolys	6/1275	33/18368	0.024306	0.21435	0.190562	TRIL/PTAF	6
MF	GO:00037	cytoskele	14/1275	114/1836	0.026326	0.220713	0.19622	MYO1G/KIF	14
MF	GO:00423	chemokine	10/1275	72/18368	0.026441	0.220713	0.19622	CXCL11/CC	10
MF	GO:00305	signaling	46/1275	495/1836	0.026525	0.220713	0.19622	CD70/ASIF	46
MF	GO:00085	phosphati	4/1275	17/18368	0.026537	0.220713	0.19622	ABCB4/ATF	4
MF	GO:00151	basic ami	4/1275	17/18368	0.026537	0.220713	0.19622	SLC38A5/S	4
MF	GO:00152	calcium-r	4/1275	17/18368	0.026537	0.220713	0.19622	RASA3/TRF	4
MF	GO:00323	MHC class	4/1275	17/18368	0.026537	0.220713	0.19622	MR1/LILRF	4
MF	GO:00053	manganese	3/1275	10/18368	0.027685	0.223006	0.198258	SLC11A2/T	3
MF	GO:00161	superoxid	3/1275	10/18368	0.027685	0.223006	0.198258	CYBB/NCF1	3
MF	GO:00345	phosphati	3/1275	10/18368	0.027685	0.223006	0.198258	INPP4B/PI	3
MF	GO:00527	1-acyl-2-	3/1275	10/18368	0.027685	0.223006	0.198258	PLAAT1/PL	3
MF	GO:00156	tubulin b	36/1275	375/1836	0.030216	0.241489	0.21469	LRRK2/MAF	36
MF	GO:00016	G protein	5/1275	26/18368	0.030977	0.242845	0.215895	CXCR6/CCF	5
MF	GO:00049	chemokine	5/1275	26/18368	0.030977	0.242845	0.215895	CXCR6/CCF	5
MF	GO:00306	GTPase re	45/1275	488/1836	0.031335	0.242845	0.215895	LRRK2/ARF	45
MF	GO:00605	nucleosid	45/1275	488/1836	0.031335	0.242845	0.215895	LRRK2/ARF	45
MF	GO:00168	hydro-lya	9/1275	64/18368	0.03187	0.242916	0.215958	DGLUCY/CA	9
MF	GO:00052	intracell	4/1275	18/18368	0.032294	0.242916	0.215958	ANO6/ANO1	4
MF	GO:00452	CXCR chem	4/1275	18/18368	0.032294	0.242916	0.215958	CXCL11/CX	4
MF	GO:00617	intracell	4/1275	18/18368	0.032294	0.242916	0.215958	ANO6/ANO1	4
MF	GO:00047	non-membr	7/1275	45/18368	0.034177	0.255206	0.226885	MATK/LYN/	7
MF	GO:00166	oxidoredu	8/1275	55/18368	0.035003	0.256753	0.22826	CLIC2/MGS	8
MF	GO:00513	alpha-act	5/1275	27/18368	0.035915	0.256753	0.22826	PDLIM1/PF	5
MF	GO:01403	ATPase-co	5/1275	27/18368	0.035915	0.256753	0.22826	ABCB4/ATF	5

MF	GO:00052[illegible]	voltage-[illegible]	3/1275	11/18368	0.036141	0.256753	0.22826	ANO6/ANO[illegible]	3
MF	GO:00171[illegible]	phosphol[illegible]	3/1275	11/18368	0.036141	0.256753	0.22826	ANO6/PLS[illegible]	3
MF	GO:00198[illegible]	IgG bind[illegible]	3/1275	11/18368	0.036141	0.256753	0.22826	FCGR1A/F[illegible]	3
MF	GO:00527[illegible]	phosphat[illegible]	3/1275	11/18368	0.036141	0.256753	0.22826	PLAAT1/P[illegible]	3
MF	GO:00052[illegible]	sodium c[illegible]	7/1275	46/18368	0.037988	0.268012	0.23827	SLC4A11/[illegible]	7
MF	GO:00512[illegible]	NAD bind[illegible]	8/1275	56/18368	0.03845	0.269412	0.239514	ME1/QDPR/	8
MF	GO:00053[illegible]	lipid tr[illegible]	17/1275	154/1836[illegible]	0.038777	0.269855	0.239908	ANO6/APO[illegible]	17
MF	GO:00518[illegible]	glycolip[illegible]	5/1275	28/18368	0.041311	0.285546	0.253858	CLIP3/LY[illegible]	5
MF	GO:00055[illegible]	phosphol[illegible]	8/1275	57/18368	0.042117	0.289163	0.257074	ANO6/ABC[illegible]	8
MF	GO:00228[illegible]	alanine [illegible]	3/1275	12/18368	0.04576	0.298618	0.265479	SLC1A4/S[illegible]	3
MF	GO:00323[illegible]	inhibito[illegible]	3/1275	12/18368	0.04576	0.298618	0.265479	LILRB5/L[illegible]	3
MF	GO:00353[illegible]	Toll-lik[illegible]	3/1275	12/18368	0.04576	0.298618	0.265479	SYK/TLR1/	3
MF	GO:19022[illegible]	voltage-[illegible]	3/1275	12/18368	0.04576	0.298618	0.265479	KCNE4/KC[illegible]	3
MF	GO:00305[illegible]	ankyrin [illegible]	4/1275	20/18368	0.045829	0.298618	0.265479	FLNC/PTP[illegible]	4
MF	GO:00316[illegible]	G-protei[illegible]	4/1275	20/18368	0.045829	0.298618	0.265479	RGS11/F2[illegible]	4
MF	GO:00350[illegible]	phosphat[illegible]	4/1275	20/18368	0.045829	0.298618	0.265479	PIK3R6/S[illegible]	4
MF	GO:00970[illegible]	ceramide [illegible]	4/1275	20/18368	0.045829	0.298618	0.265479	CLIP3/CD[illegible]	4
MF	GO:00480[illegible]	CCR chem[illegible]	7/1275	48/18368	0.04642	0.300552	0.267199	CCL18/CC[illegible]	7
MF	GO:00611[illegible]	peptidas[illegible]	23/1275	230/1836[illegible]	0.049561	0.318872	0.283485	PCOLCE2/[illegible]	23
