# Supplementary material for: Identification of Immune-Related lncRNAs for Predicting Prognosis and Immune Landscape Characteristics of Uveal Melanoma
Source: J Oncol. 2022 Aug 29;2022:7680657. doi: 10.1155/2022/7680657 (PMC9668462; doi:10.1155/2022/7680657)
Supplement: Supplementary Materials — Figure S1: (a) Kaplan–Meier survival curve, the expression of the 3 prognostic irlncRNAs, patterns of survival outcome, and distribution of risk score for patients between different groups in the training set. (b) Kaplan–Meier survival curve, the expression of the 3 prognostic irlncRNAs, patterns of survival outcome, and distribution of risk score for patients between different groups in the testing set. Figure S2: (a, b): the ROC curves demonstrated the high sensitivity and specificity of the signature for survival prediction, and the one-, three-, and five-year AUC values, respectively, were 0.967, 0.886, and 0.964 in the testing set and 0.974, 0.924, and 0.939 in the training set. (c) The calibration plot of the nomogram predicting the probability of the one-, three-, and five-year prognosis. Figure S3: identification of potential drugs targeting the model (P < 0.05). Table S1: identified 409 prognostic irlncRNAs. Table S2: the baseline features of these datasets, demonstrating no statistically significant variations in clinical features (p > 0.05). Table S3: original data of GO. Table S4: original data of KEGG. . [file 7680657.f1.zip › 7680657.f1/Supplementary Table4.pdf]

ID	Descripti	GeneRatic	BgRatio	pvalue	p.adjust	qvalue	geneID	Count
hsa05330	Allograft	21/619	38/8157	1.85E-14	5.89E-12	4.93E-12	HLA-A/HLA	21
hsa04514	Cell adhe	42/619	157/8157	1.74E-13	2.75E-11	2.30E-11	CD58/HLA-	42
hsa05332	Graft-ver	21/619	42/8157	2.59E-13	2.75E-11	2.30E-11	HLA-A/HLA	21
hsa04940	Type I di	21/619	43/8157	4.71E-13	3.74E-11	3.14E-11	HLA-A/HLA	21
hsa05416	Viral myc	24/619	60/8157	2.30E-12	1.46E-10	1.22E-10	HLA-A/HLA	24
hsa04145	Phagosome	39/619	152/8157	5.46E-12	2.89E-10	2.42E-10	TCIRG1/HL	39
hsa05320	Autoimmur	21/619	53/8157	6.88E-11	3.13E-09	2.62E-09	HLA-A/HLA	21
hsa04658	Th1 and T	27/619	92/8157	4.16E-10	1.65E-08	1.38E-08	HLA-DMA/H	27
hsa05150	Staphyloc	26/619	96/8157	5.87E-09	2.08E-07	1.74E-07	HLA-DMA/H	26
hsa04659	Th17 cell	27/619	108/8157	1.96E-08	6.25E-07	5.23E-07	HLA-DMA/H	27
hsa04612	Antigen p	22/619	78/8157	3.94E-08	1.14E-06	9.54E-07	HLA-A/TAP	22
hsa05323	Rheumatoi	24/619	93/8157	6.30E-08	1.67E-06	1.40E-06	TCIRG1/AT	24
hsa05140	Leishmani	21/619	77/8157	1.52E-07	3.71E-06	3.11E-06	EEF1A2/HL	21
hsa05152	Tuberculo	35/619	180/8157	1.64E-07	3.73E-06	3.12E-06	TCIRG1/CA	35
hsa04640	Hematopoi	24/619	99/8157	2.27E-07	4.80E-06	4.02E-06	ITGA1/HLA	24
hsa05321	Inflammat	18/619	65/8157	9.20E-07	1.83E-05	1.53E-05	HLA-DMA/H	18
hsa05169	Epstein-B	36/619	202/8157	1.02E-06	1.90E-05	1.59E-05	SAP30/CD5	36
hsa05310	Asthma	12/619	31/8157	1.21E-06	2.14E-05	1.79E-05	HLA-DMA/H	12
hsa04650	Natural k	27/619	131/8157	1.33E-06	2.22E-05	1.86E-05	HLA-A/HLA	27
hsa04060	Cytokine-	46/619	295/8157	1.73E-06	2.75E-05	2.30E-05	CXCR6/CD7	46
hsa04672	Intestina	15/619	49/8157	1.86E-06	2.82E-05	2.36E-05	HLA-DMA/H	15
hsa04061	Viral pro	21/619	100/8157	1.47E-05	0.000212	0.000178	CCR1/ACKR	21
hsa05145	Toxoplasm	22/619	112/8157	2.80E-05	0.000387	0.000324	LAMA5/PIK	22
hsa05322	Systemic	24/619	136/8157	7.64E-05	0.001012	0.000847	HLA-DMA/H	24
hsa05166	Human T-c	33/619	222/8157	0.000134	0.001698	0.001422	HLA-A/EGR	33
hsa05163	Human cyt	33/619	225/8157	0.000173	0.002118	0.001773	CTNNB1/CC	33
hsa05170	Human imm	31/619	212/8157	0.000286	0.003365	0.002818	BST2/HLA-	31
hsa05133	Pertussis	15/619	76/8157	0.000491	0.005581	0.004674	C4A/C1QA/	15
hsa04062	Chemokine	28/619	192/8157	0.00058	0.006361	0.005327	CXCR6/CCR	28
hsa04512	ECM-recep	16/619	88/8157	0.000836	0.008858	0.007419	ITGA1/LAM	16
hsa04670	Leukocyte	19/619	114/8157	0.00087	0.008921	0.007471	RASSF5/CT	19
hsa05164	Influenza	25/619	171/8157	0.001083	0.010761	0.009012	PRKCA/HLA	25
hsa04610	Complemen	15/619	85/8157	0.001646	0.015862	0.013284	SERPINE1/	15
hsa05340	Primary i	9/619	38/8157	0.00174	0.016195	0.013563	TAP1/CD8A	9
hsa04974	Protein d	17/619	103/8157	0.001782	0.016195	0.013563	COL5A1/SL	17
hsa05165	Human pap	40/619	331/8157	0.002084	0.01841	0.015417	TCIRG1/IT	40
hsa04666	Fc gamma	16/619	97/8157	0.002419	0.020794	0.017414	WAS/ASAP1	16
hsa04662	B cell re	14/619	82/8157	0.003194	0.026517	0.022207	LYN/INPP5	14
hsa00480	Glutathio	11/619	57/8157	0.003252	0.026517	0.022207	RRM2B/LAP	11
hsa04380	Osteoclas	19/619	128/8157	0.003474	0.027619	0.02313	NCF4/SPI1	19
hsa04620	Toll-like	16/619	104/8157	0.004938	0.036521	0.030585	MAP3K8/CX	16
hsa04625	C-type le	16/619	104/8157	0.004938	0.036521	0.030585	EGR2/LSP1	16
hsa04660	T cell re	16/619	104/8157	0.004938	0.036521	0.030585	MAP3K8/CD	16
hsa05205	Proteogly	26/619	205/8157	0.006439	0.046534	0.038971	CAMK2A/CT	26
hsa03320	PPAR sign	12/619	75/8157	0.010297	0.072765	0.060939	ACSBG1/PP	12
hsa05167	Kaposi sa	24/619	194/8157	0.011621	0.080333	0.067277	CTNNB1/CC	24
hsa04510	Focal adh	24/619	201/8157	0.017418	0.115583	0.096798	ITGA1/LAM	24
hsa05418	Fluid she	18/619	139/8157	0.017447	0.115583	0.096798	CTNNB1/VC	18
hsa04623	Cytosolic	10/619	63/8157	0.019327	0.123976	0.103826	STING1/CC	10
hsa05143	African t	7/619	37/8157	0.019493	0.123976	0.103826	IDO1/VCAM	7

hsa05417	Lipid anc	25/619	215/8157	0.020883	0.127922	0.107131	CAMK2A/VC	25
hsa04115	p53 signa	11/619	73/8157	0.020918	0.127922	0.107131	SERPINE1/	11
hsa05142	Chagas di	14/619	102/8157	0.02141	0.128457	0.107579	SERPINE1/	14
hsa04064	NF-kappa	14/619	104/8157	0.024928	0.146799	0.12294	GADD45G/V	14
hsa04015	Rap1 sigr	24/619	210/8157	0.02798	0.161774	0.135481	RASSF5/CT	24
hsa05412	Arrhythmo	11/619	77/8157	0.02992	0.169903	0.142289	ITGA1/CTN	11
hsa04070	Phosphati	13/619	97/8157	0.030987	0.172002	0.144047	PLCD1/INF	13
hsa04664	Fc epsilo	10/619	68/8157	0.031371	0.172002	0.144047	LYN/PRKCA	10
hsa05144	Malaria	8/619	50/8157	0.033112	0.178469	0.149463	VCAM1/PEC	8
hsa00920	Sulfur me	3/619	10/8157	0.03488	0.183627	0.153782	PAPSS2/SG	3
hsa00910	Nitrogen	4/619	17/8157	0.035302	0.183627	0.153782	CA8/CA12/	4
hsa05235	PD-L1 exp	12/619	89/8157	0.035801	0.183627	0.153782	CD247/BAT	12
hsa00590	Arachidor	9/619	61/8157	0.039275	0.197996	0.165816	CBR3/TBXA	9
hsa04919	Thyroid h	15/619	121/8157	0.039848	0.197996	0.165816	PLN/PLCD1	15
hsa05171	Coronavir	25/619	232/8157	0.046611	0.228037	0.190974	RPL24/PRK	25
